# SARS-CoV-2 primed platelets–derived microRNAs enhance NETs formation by extracellular vesicle transmission and TLR7/8 activation

**DOI:** 10.1186/s12964-023-01345-4

**Published:** 2023-10-30

**Authors:** Tsai-Ling Liao, Hung-Jen Liu, Der-Yuan Chen, Kuo-Tung Tang, Yi-Ming Chen, Po-Yu Liu

**Affiliations:** 1 Department of Medical Research, Taichung Veterans General Hospital, No.1650, Sec.4, Taiwan Boulevard, Xitun Dist, Taichung City, 407 Taiwan; 2 Rong Hsing Translational Medicine Research Center, National Chung Hsing University, Taichung, 402 Taiwan; 3 Doctoral Program in Translational Medicine, National Chung Hsing University, Taichung, 402 Taiwan; 4 Institute of Molecular Biology, National Chung Hsing University, Taichung, 402 Taiwan; 5 The iEGG and Animal Biotechnology Center, National Chung Hsing University, Taichung, 402 Taiwan; 6 Rheumatology and Immunology Center, China Medical University Hospital, Taichung, 404 Taiwan; 7 Translational Medicine Laboratory, Rheumatology and Immunology Center, China Medical University Hospital, Taichung, 404 Taiwan; 8 College of Medicine, China Medical University, Taichung, 404 Taiwan; 9 Institute of Medicine, Chung Shan Medical University Hospital, Taichung, 402 Taiwan; 10 Division of Allergy, Immunology and Rheumatology, Taichung Veterans General Hospital, Taichung, 407 Taiwan; 11 Division of Infection, Department of Internal Medicine, Taichung Veterans General Hospital, No.1650, Sec.4, Taiwan Boulevard, Xitun Dist, Taichung City, 407 Taiwan

**Keywords:** COVID-19, extracellular vesicles, Platelets, NETs, microRNAs, TLR8

## Abstract

**Background:**

Hyperactive neutrophil extracellular traps (NETs) formation plays a key role in the pathogenesis of severe COVID-19. Extracellular vesicles (EVs) are vehicles which carry cellular components for intercellular communication. The association between COVID-19 patients-derived EVs and NETs formation remains elusive.

**Methods:**

We explored the roles of EVs in NETs formation from 40 COVID-19 patients with different disease severities as well as 30 healthy subjects. The EVs-carried microRNAs profile was analyzed using next generation sequencing approach which was validated by quantitative reverse transcription PCR. The regulatory mechanism of EVs on NETs formation was investigated by using an in vitro cell-based assay, including immunofluorescence assay, flow cytometry, and immunoblotting.

**Results:**

COVID-19 patient–derived EVs induced NETs formation by endocytosis uptake. SARS-CoV-2 spike protein-triggered NETs formation was significantly enhanced in the presence of platelet–derived EVs (pEVs) and this effect was Toll-like receptor (TLR) 7/8- and NADPH oxidase-dependent. Increased levels of miR-21/let-7b were revealed in EVs from COVID-19 patients and were associated with disease severity. We demonstrated that the spike protein activated platelets directly, followed by the subsequent intracellular miR-21/let-7b upregulation and then were loaded into pEVs. The pEVs-carried miR-21 interacted with TLR7/8 to prime p47phox phosphorylation in neutrophils, resulting in NADPH oxidase activation to promote ROS production and NETs enhancement. In addition, miR-21 modulates NF-κB activation and IL-1β/TNFα/IL-8 upregulation in neutrophils upon TLR7/8 engagement. The miR-21 inhibitor and TLR8 antagonist could suppress efficiently spike protein-induced NETs formation and pEVs primed NETs enhancement.

**Conclusions:**

We identified SARS-CoV-2 triggered platelets–derived GU-enriched miRNAs (e.g., miR-21/let-7b) as a TLR7/8 ligand that could activate neutrophils through EVs transmission. The miR-21-TLR8 axis could be used as a potential predisposing factor or therapeutic target for severe COVID-19.

**Supplementary Information:**

The online version contains supplementary material available at 10.1186/s12964-023-01345-4.

## Abstract (highlights)

COVID-19 poses a crucial threat to global health. Excessive NETs contribute to microthrombi through platelet-neutrophil interactions, resulting in acute respiratory distress syndrome in severe COVID-19 cases. EVs play a crucial role in intercellular communication during infection. Herein, we showed that the viral spike protein activates platelets directly, followed by intracellular miR21/let7b upregulation, which is loaded into pEVs. The pEV-carried miR-21/let-7b could interact with TLR7/8 in neutrophils, followed by activation of the downstream signaling pathway, including p47phox-NOX2-ROS, which causes NETs enhancement, while NF-κB promotes the expression of proinflammatory cytokines. The miR-21 inhibitor and TLR8 antagonist could efficiently suppress spike protein-induced NETs formation and pEVs primed NETs enhancement. This study offers new molecular machinery to explain the association between platelets–derived miRNAs, NETs formation and SARS-CoV-2 infection. Host-directed therapies are a relatively new and promising approach to COVID-19 treatment. The miR-21-TLR8 axis may be a potential therapeutic target for severe COVID-19.

Video Abstract

**Supplementary Information:**

The online version contains supplementary material available at 10.1186/s12964-023-01345-4.

## Background

Coronavirus disease-19 (COVID-19) is caused by respiratory tract infection with severe acute respiratory syndrome coronavirus 2 (SARS-CoV-2) [1]. The clinical spectrum of COVID-19 ranges from asymptomatic to severe disease [2]. Patients with severe COVID-19 disease show a dysregulated hyperactivation of the immune system that can cause an abnormal cytokine immune response [3].

Neutrophils have a variety of important biological functions in both innate and adaptive immunities, thus playing a key role in infection. Neutrophil extracellular traps (NETs) are extracellular webs of chromatin, microbicidal proteins and oxidant enzymes that are released by neutrophils to contribute to pathogen clearance [4]. When neutrophils interact directly with platelets and plasma coagulation factors causing coagulopathy and thrombosis, this is known as immunothrombosis, which promotes the defense against pathogens [5]. Aberrant, or uncontrolled activation of immunothrombosis may be damaging to the host [6]. Accumulating evidence has shown that SARS-CoV-2-induced hyperactive NETs formation can trigger uncontrolled immunothrombosis and play a crucial role in COVID-19 pathology [7–9]. However, what triggers the imbalance in the dysregulated NETs formation and coagulation system in severe COVID-19 is currently poorly understood.

Extracellular vesicles (EVs) are small membrane-bound vesicles released by cells to carry proteins and nucleic acids to contribute to intercellular communication and regulate biological functions [10]. A recent study showed that severe COVID-19 patient–derived EVs could promote neutrophil adhesion and induce NETs production [11]. Platelets are primary producing source of circulating EVs [12] which are implicated in thrombosis [13]. In addition, platelets can interact with viruses and are an important source of inflammatory mediators [14]. Zaid et al. demonstrated that platelets are associated with SARS-CoV-2 RNA and are hyperactivated in COVID-19 [15]. Accumulating evidence showed that elevated levels of platelet–derived EVs (pEVs) were revealed in the blood of patients with COVID-19 [11, 15]. We hypothesized that SARS-CoV-2 induced platelets to produce specific components (e.g., miRNAs) that may regulate NETs formation by pEV transmission, resulting in a key mode of intercellular communication against SARS-CoV-2 infection. The aim of this study was to explore the roles of pEVs in hyperactive NETs formation in patients with severe COVID-19.

## Methods

### Subjects

This prospective study was conducted from 2020 to 2022. A total of 40 patients with COVID-19 and 30 healthy subjects were analyzed in this study. Of these, 20 COVID-19 patients and 30 healthy subjects were enrolled from Taichung Veterans General Hospital (TCVGH) in Taiwan. The other 20 COVID-19 samples were purchased from the National Health Research Institute Biobank in Taiwan. COVID-19 patients were confirmed by using RT-PCR to detect SARS-CoV-2 RNA. The exclusion criteria in this study were: (1) individuals with autoimmune diseases, cancer, history of bleeding disorders or anticoagulant therapy; (2) current treatment with immune-modulating drugs; and (3) pregnancy.

Patients were classified according to their severity grade during the evolution of COVID-19 as follows: (1) Severe: intensive care unit (ICU) admission, invasive mechanical ventilation, or the presence of bilateral pulmonary infiltrates and mechanical ventilation; (2) Moderate: the remaining patients admitted to hospital who did not fulfil severe COVID-19 criteria; (3) Mild/Asymptomatic: individuals with minor or no COVID-19 symptoms. This study was conducted in compliance with the Declaration of Helsinki and has been approved by the Institutional Review Board of Taichung Veterans General Hospital (CE21403B). The study methods were carried out in accordance with the approved guidelines and written consent was obtained from all participants.

### Cell culture

The human promyelocytic leukemia cell lines HL-60 cells (ATCC CCL-240) were grown in RPMI medium 1640 (Thermo Fisher Scientific, USA) supplemented with 10% fetal bovine serum (FBS), 1 × nonessential amino acids, 100 units/ml penicillin, 100 units/ml streptomycin, in an incubator containing 5% CO_2_ at 37 °C. To readily induce differentiation into neutrophil-like cells (dHL-60), HL-60 cells were grown in media and treated with 1.3% DMSO (Sigma-Aldrich, USA) for 72 h. The HEK hTLR7/8 stable cell line was purchased from InvivoGen (USA) and cultured in Dulbecco’s modified Eagle’s medium (DMEM) (Thermo Fisher Scientific, USA), according to the manufacturer’s protocol. For EVs study, the cells were cultured in media supplemented with 10% exosome-depleted FBS (Thermo Fisher Scientific, USA), in an incubator containing 5% CO_2_ at 37 °C.

### Neutrophils and platelets isolation

Neutrophils were immediately isolated from venous blood by using Polymorphprep (Axis-Shield, USA), according to the manufacturer’s instructions. After centrifugation at 500 × g for 30 min at 25 °C with low brake, neutrophils were sunk to the middle polymorphonuclear leukocyte (PMN) layer of the solution. The PMN layer was transferred to a clean tube and then the ammonium-chloride-potassium (ACK) lysing buffer (Thermo Fisher Scientific, USA) was added and mixed gently. After 5 min, the mixtures were centrifuged at 500 × g for 5 min at 25 °C. The pellets were suspended in 10 ml Hank's balanced salt solution (HBSS, Sigma-Aldrich, USA) and dispersed gently. After centrifugation at 500 × g for 5 min at 25 °C, the neutrophil pellets were obtained and suspended in 1 ml HBSS.

Platelets were harvested from peripheral blood by centrifugation at 230 × g for 15 min at 24 °C, followed by centrifugation at 1000 × g for 10 min at 24 °C. Pellets were resuspended with Tyrode’s buffer (Sigma-Aldrich, USA) containing 1/6 volumes of acid citrate dextrose (ACD) anticoagulant (Sigma-Aldrich, USA) and 1 μM Prostaglandin I2 (PGI2) (Sigma-Aldrich, USA) and then centrifuged at 1000 × g for 10 min at 24 °C before being resuspended in Tyrode’s buffer containing 1 μM PGI2 and 0.04 U/ml apyrase (Sigma-Aldrich, USA) and placed on the shaker at 24 °C. Before use, samples were subjected to centrifugation at 1000 × g for 10 min at 24 °C and were resuspended in Tyrode’s buffer.

To explore the effects of SARS-CoV-2 spike protein on platelet–derived EVs production, recombinant spike protein (2 μg, MyBioSource, USA) was added to platelets for the indicated time, culture medium was collected and EVs were isolated and quantified.

### EVs isolation and quantification

Samples were centrifuged at 350 × g for 10 min at 4 °C to remove cell debris, then filtered through a 0.22 μm filter. For EVs characteristics analysis and functional assays, 2.5 ml of samples were diluted with 7.5 ml of PBS and concentrated using Amicon ultra-0.5 centrifugal filter devices (Millipore, Amicon Ultra 100 K device) at 3000 × g for 30 min at 4 °C. One hundred µl retentate was diluted with 1.4 ml of PBS and subjected to centrifugation at 10,000 × g for 30 min at 4 °C. The pellets were resuspended in 1.5 ml PBS and ultracentrifuged at 120,000 × g for 90 min at 4 °C. The pellets were resuspended in 50 µl PBS and stored at -80 °C. For quantitative reverse transcription PCR (QRT-PCR) validation analysis, the plasma–derived EVs were exacted by ExoQuick exosome precipitation solution (System Biosciences, USA) according to the manufacturer’s instructions. The purified EVs were confirmed using transmission electron microscope images analysis, the nanoparticle tracking assay, immunoblotting and flow cytometry, respectively. The EVs were quantified using a direct ELISA-based method to quantify the EVs surface marker CD63 according to the manufacturer’s instructions (System Biosciences, USA).

### EVs-carried microRNAs quantitative PCR

The total EV-carried miRNAs were extracted from the EVs using Total Exosome RNA & Protein Isolation Kit (Thermo Fisher Scientific, USA) and purified by RNeasy MinElute® Cleanup Kit (QIAGEN, Germany) according to the manufacturer’s instructions. Twenty-five femtomoles of synthetic *Caenorhabditis elegans* miRNA (cel-miR-39, Thermo Fisher Scientific, USA) were added to each sample as the internal control. The purified miRNAs were quantified at OD260 and 280 nm by using an ND-1000 spectrophotometer (Thermo Fisher Scientific, USA). The miRNA expression was quantified using the TaqMan MicroRNA assays kit (Thermo Fisher Scientific, USA) according to the manufacturer’s protocol. QRT-PCR analysis were performed on the StepOnePlus Real-Time PCR System (Thermo Fisher Scientific, USA), using a standard protocol.

### Loading of miRNAs mimic, or control into EVs

Electroporation was used in loading miR-21/let-7b mimic, or control into human platelet–derived EVs. In brief, 0.1 µmole of miR-21/let-7b mimic, or control (Thermo Fisher Scientific, USA) was added to 20 µl of platelet–derived EV samples (approximately 5 × 10^8^ particles). The mixtures were electroporated at 500 pulse voltage/10 pulse width (ms)/3 pulses using a Neon Transfection system (Thermo Fisher Scientific). After electroporation, the mixture was immediately treated with one unit of RNase A (QIAGEN, Germany) for 30 min, followed by the addition of 2 µl RNase inhibitor. MiR-21/let-7b mimic- or control-loaded EVs were extracted using the ExoQuick exosome precipitation solution (System Biosciences, USA) according to the manufacturer’s instructions.

### Immunofluorescence assay (IFA)

Human neutrophils or dHL-60 cells with individual treatment were fixed with 4% paraformaldehyde at room temperature for 10 min and then washed three times with PBS. Cells were permeabilized in PBS containing 1% BSA and 0.2% saponin and then blocked for 1 h in PBS containing 2% BSA. For NETs formation analysis, cells were incubated with the primary antibodies [mouse myeloperoxidase (MPO) antibody (Santa Cruz, USA) at 1:200] followed by a secondary antibody for MPO detection. DNA was stained with Hoechst 33342 (Thermo Fisher Scientific, USA) and extracellular NETs DNA was detected with a cell impermeable, extracellular DNA dye Sytox Green (5 μM, Thermo Fisher Scientific) for 10 min after fixation and permeabilization. The staining procedures were carried out at room temperature by protecting the samples from direct light. Coverslips were mounted onto glass slides with Slow-Fade mounting medium (Thermo Fisher Scientific, USA) and images were observed and recorded on an Olympus FV1000 laser scanning confocal microscope. Images were analyzed using FV10-ASW version 4.2 software.

### Quantification of NETs DNA

Briefly, 100 μl of plasma or neutrophil culture supernatant was incubated overnight in a 96-well plate precoated with anti-MPO antibody (Santa Cruz, USA). The DNA bound to MPO was quantified using the Quant-iT PicoGreen kit (Thermo Fisher Scientific, USA) according to the manufacturer’s instructions.

### Immunoblotting

The cells with different treatments were lysed in RIPA buffer (25 mM Tris–HCl pH 7.6, 150 mM NaCl, 1% NP-40, 1% sodium deoxycholate and 0.1% SDS) containing a protease inhibitor cocktail (Complete, Roche, Germany). Twenty micrograms of total protein from exosome lysate were loaded and separated on a standard sodium dodecyl sulfate (SDS)-polyacrylamide gel electrophoresis (PAGE) gel and transferred to a polyvinylidene difluoride (PVDF) membrane (Millipore, USA). The membranes were incubated with primary antibodies, followed by peroxidase-conjugated secondary antibodies. The results were detected using a charge-coupled device (CCD) camera-based imager (GE Healthcare Life Sciences, USA) after membrane incubation with enhanced chemiluminescence (ECL) substrates (Millipore, USA). The levels of specific protein were normalized to β-actin. The ImageJ software was used for image acquisition and densitometric analysis of the immunoblots. All results were obtained in three independent experiments and the data are presented as the mean ± SD. An unpaired, two-tailed Student’s t-test was performed for between-group comparisons using GraphPad Prism software version 8. All results of densitometric analysis are presented in Additional file 2.

### Flow cytometry

Platelet- or EV-specific surface marker staining for EVs was performed using the Exo-Flow exosome capture kit (System Biosciences, USA) according to the manufacturer’s instructions. Briefly, purified EVs were mixed with immune-magnetic beads coated with anti-human CD63 monoclonal antibody (BioLegend, USA) and incubated overnight at 4 °C. After incubation, the beads were washed using Bead Wash buffer (System Biosciences, USA). The EVs were incubated with the Exo-FITC exosome stain (System Biosciences, USA) for exosome detection, AF647-conjugated anti-CD41 monoclonal antibody for platelet-surface marker detection (BD Biosciences, USA), respectively; then, they were examined by flow cytometry (FACSCanto II, BD Biosciences, USA). AF647-conjugated IgG1 (BD Biosciences, USA) was used as an isotype control. All data were analyzed using the CellQuest (BD Biosciences) or FlowJo software.

### Quantification of ROS production

The levels of cytosolic ROS were analyzed by using fluorescent dye dihydrorhodamine (DHR) 123 (Thermo Fisher Scientific) staining and quantified by flow cytometry. Data of flow cytometry were analyzed by the CellQuest software and expressed as the mean fluorescence intensity (MFI) of cytosolic ROS.

### Statistics

The results are presented as the mean ± standard deviation (SD). An unpaired, two-tailed Student’s t-test was used for between-group comparisons. A one-way analysis of variance (ANOVA) with the post hoc Bonferroni test was used for multiple comparisons. The correlation coefficient was calculated using Spearman’s correlation test. *P* values less than 0.05 were considered statistically significant and tests were performed using GraphPad Prism 8.

## Results

### Clinical characteristics of COVID-19 patients

A total of 70 participants, including 40 COVID-19 patients and 30 healthy subjects, were enrolled. Among the COVID-19 patients, ten were admitted to the ICU (25%) and four with severe COVID-19 required invasive mechanical ventilation (10%). Patients averaged 7.3 days from the first symptoms of COVID-19 to ICU admission and remained in the ICU for an average of 13.2 days. Thirty patients were asymptomatic or had mild symptom (75%).

### Circulating EVs induced NETs formation in COVID-19 patients

**Fig. 1:**
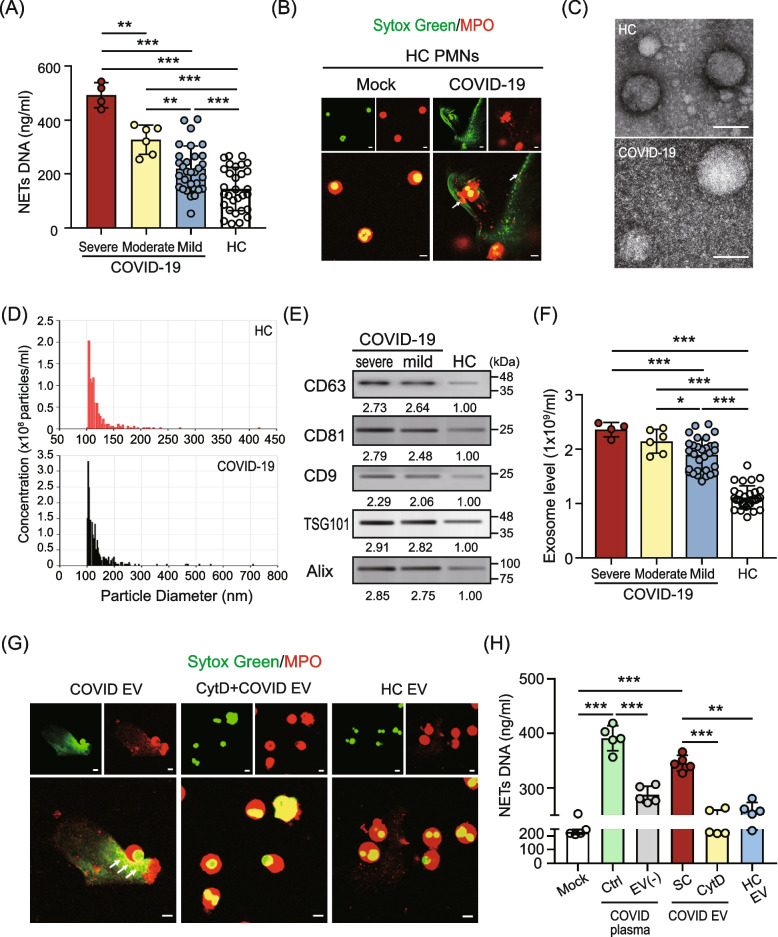
Circulating EVs induced NETs formation in COVID-19 patients. **A** Increased levels of NETs DNA were detected in the plasma from COVID-19 patients with different severities compared to those in healthy control (HC) subjects. **B** Elevated NETs DNA was released from normal human neutrophils treating plasma from COVID-19 patients. **C** Transmission electron micrographs of purified extracellular vesicles (EVs) isolated from HC (upper panel) and COVID-19 patient (lower panel). The scale bar in the image represents 100 nm. **D** The size distribution of EVs isolated from HC (upper panel) and COVID-19 patient (lower panel) were analyzed using a nanoparticle tracking assay. **E** Expression of small EV (sEV)-specific surface markers CD63, CD81, CD9, TSG101 (Tumor Susceptibility Gene 101) and Alix (ALG-2-interacting protein X) in plasma–derived EVs was analyzed using immunoblotting. **F** Comparison of plasma–derived EVs levels in COVID-19 patients with different severity and HC subjects. The plasma–derived EV levels in patients were quantified using enzyme-linked immunosorbent assay (ELISA)-based assay. **G** Increased levels of NETs DNA were detected in normal human neutrophils after treating with COVID-19 patient–derived EVs (left panel); this effect was decreased in the presence of the endocytosis inhibitor cytochalasin D (Cyt D, 10 μM) (middle panel). **H** The quantification of NETs DNA released from normal human neutrophils with different stimulation. The scale bar in the IFA image represents 5 μm. All experiments were performed in triplicate and data were presented as mean ± SD. *P < 0.05, **P < 0.01, ***P < 0.005

We analyzed the levels of NETs DNA release in COVID-19 patients with different disease severities and HC subjects. Elevated levels of NETs DNA were detected in COVID-19 patients with different severities (severe: *n* = 4, 492.1 ± 46.1 ng/ml; moderate: *n* = 6, 327.2 ± 54.4 ng/ml,* P* < 0.005; mild: *n* = 30, 219.3 ± 84.6 ng/ml, *P* < 0.005; Fig. 1A), compared to those in HC subjects (*n* = 30, 143.9 ± 80.2 ng/ml). In addition, our data showed that the level of NETs DNA is associated with disease severity, which is consistent with other reports [7–9].

To explore the regulatory mechanism of NETs enhancement in severe COVID-19 patients, we stimulated normal human neutrophils with the plasma of severe COVID-19 patients for 4 h. The levels of extracellular NETs were detected with a cell impermeable, extracellular DNA dye called Sytox Green. Dramatically elevated NETs release was observed by using confocal microscopy analysis (Fig. 1B), suggesting that the components in the plasma from COVID-19 patients may contribute to NETs formation.

EVs can circulate through various bodily fluids (e.g., plasma) and play a key role in intercellular communication during infection [10]. We hypothesized that SARS-CoV-2-induced cellular components may be carried by EVs to mediate NETs formation. Initially, we analyzed the particle size of EVs in the plasma of patients with COVID-19 by using transmission electron microscope images analysis (Fig. 1C) and a nanoparticle tracking assay (Fig. 1D), respectively. The results show that the average particle size of EVs from COVID-19 patient is 135.80 nm, which is consistent with the size of typical small EVs (sEVs, < 200 nm) [16]. In addition, the EVs size of COVID-19 patient is slightly larger than that of HC subject (119.0 nm). We detected sEV-specific (CD63, CD81, CD9, TSG101, and Alix) surface markers using immunoblotting. The results showed that COVID-19 patient–derived EVs with the expression of specific sEV markers and their levels are higher than those in HC subjects (Fig. 1E).

We further compared the levels of EVs in the plasma of COVID-19 patients with different disease severities by using an ELISA-based assay (Fig. 1F). Significantly increased levels of circulating EVs were observed in the plasma of COVID-19 patients compared with those in HC subjects and are associated with disease severity (severe: 2.36 ± 0.13 × 10^9^ EV particles/ml; moderate: 2.15 ± 0.22 × 10^9^ EV particles/ml; mild: 1.90 ± 0.30 × 10^9^ EV particles/ml versus HC: 1.12 ± 0.21 × 10^9^ EV particles/ml, *P* < 0.005).

To explore whether the EV is a key factor in the regulation of NETs formation during SARS-CoV-2 infection, normal human neutrophils were co-cultured with COVID-19 patient– or HC–derived EVs for 4 h, respectively. The levels of NETs formed were observed (Fig. 1G) and quantified (Fig. 1H). Dramatically elevated NETs formation was shown in normal neutrophils co-cultured with plasma from COVID-19 patients (*n* = 5, 391.32 ± 22.94 ng/ml versus 229.60 ± 18.36 ng/ml, *P* < 0.005), but this effect was decreased in those co-cultured with EVs-depleted plasma (287.51 ± 15.50 ng/ml, *P* < 0.005), suggesting that EVs may be a major inducer in the plasma for NETs formation. Significantly increased NETs formation was shown in neutrophils after treatment with COVID-19 patient–derived EVs (*n* = 5, 345.30 ± 14.40 ng/ml), compared with those in HC–derived EVs (*n* = 5, 258.12 ± 14.98 ng/ml, *P* < 0.01) or control cells (229.60 ± 18.36 ng/ml, *P* < 0.005). This effect decreased in the presence of the endocytosis inhibitor cytochalasin D (237.50 ± 21.48 ng/ml, *P* < 0.005), suggesting that COVID-19 patient–derived EVs induced NETs formation by uptake.

### COVID-19 patient–derived EVs induced NETs formation through TLR7/8 activation and NADPH oxidase-dependent ROS production

**Fig. 2:**
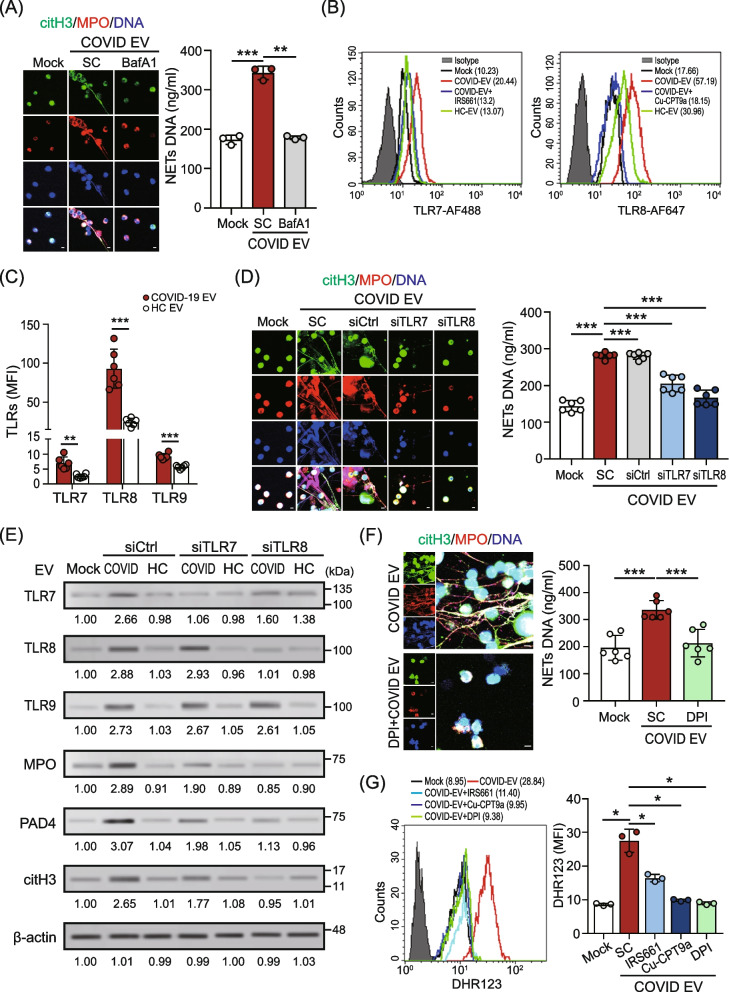
COVID-19 patient–derived EVs induced NETs formation through TLR7/8 activation and NADPH oxidase-dependent ROS production. **A** COVID-19 patient–derived EV-induced NETs formation was inhibited in the presence of the vacuolar type H^+^-ATPase inhibitor bafilomycin A1 (BafA1, 100 nM). The levels of NETs formation were observed (left panel) and quantified (right panel). SC, solvent control. **B** Human neutrophils were treated with COVID-19 patient- or healthy control (HC) subject–derived EVs in the presence or absence of the TLR7/8 specific inhibitor (10 μM). The levels of TLR7 (left panel) or TLR8 (right panel) were analyzed using flow cytometry. IRS661, TLR7-specific inhibitor; Cu-CPT9a, TLR8-specific inhibitor. **C** The levels of intracellular TLR7, TLR8 and TLR9 in the neutrophils of COVID-19 patients and HC subjects were analyzed using flow cytometry. MFI, mean fluorescence intensity. **D** Comparison of NETs formation induced by COVID-19 patient–derived EVs in TLR7- or TLR8-knockdown neutrophils. NETs formation was observed using a confocal microscope (left panel) and quantified by the MPO-DNA PicoGreen assay (right panel). **E** Human neutrophils were transfected with control siRNA, TLR7, or TLR8 siRNA (30 nM) for 24 h. The COVID-19 patient– or HC–derived EVs were added to control cells, TLR7-, or TLR8-knockdown cells, respectively. After 24 h, the expression of intracellular TLR7/8/9 and NETs-associated proteins was analyzed by using immunoblotting. Immunoblotting bands from β-actin were densitometrically measured by ImageJ to determine the lane normalization factor for samples. The image shown is from a single experiment that is representative of at least three separate experiments. **F** Human neutrophils were treated with COVID-19 patient–derived EVs in the presence or absence of the NADPH oxidase inhibitor diphenyleneiodonium (DPI, 25 μM). NETs formation was observed using a confocal microscope (left panel) and quantified by the MPO-DNA PicoGreen assay (right panel). **G** Human neutrophils were treated with COVID-19 patient–derived EVs in the presence or absence of the TLR7-, TLR8- and NADPH oxidase inhibitors. The levels of cytosolic ROS were detected using flow cytometry with dihydrorhodamine (DHR) 123 staining. The scale bar in the IFA image represents 5 μm. All experiments were performed in triplicate and data were presented as mean ± SD. *P < 0.05, **P < 0.01, ***P < 0.005

Innate immunity-associated Toll-like receptors (TLRs) play a crucial role against viral infection. We examined whether TLRs might regulate NETs formation induced by COVID-19 patient–derived EVs. As shown in Fig. 2A, the COVID-19 patient–derived EVs-induced NETs formation was suppressed completely in the presence of the vacuolar type H^+^-ATPase inhibitor bafilomycin A1 (BafA1, 100 nM), suggesting that endolysosomal TLRs such as TLR3, TLR7, TLR8 and TLR9 mediate NETs formation. Given that mature neutrophils express all TLRs except TLR3 [17], we analyzed the expressions of TLR7, TLR8 and TLR9 in normal human neutrophils after being treated with EVs from COVID-19 patients (*n* = 6) and HC subjects (*n* = 6), respectively. Compared to TLR7 and TLR9, a dramatically elevated level of TLR8 was shown in neutrophils after treatment with COVID-19 patient–derived EVs (MFI, 92.97 ± 25.07 versus 24.48 ± 5.03, *P* < 0.005, Fig. 2C), and this effect was suppressed in the presence of the TLR8 specific inhibitor Cu-CPT9a (Fig. 2B). We further examined the effects of TLRs on COVID-19 patient–derived EV-induced NETs formation. As shown in Fig. 2D, the COVID-19 patient–derived EV-induced NETs formation was reduced in TLR7- and TLR8- knockdown cells, respectively. The inhibitory effect in TLR8 knockdown cells is better than in TLR7 knockdown cells. The immunoblotting results revealed that the levels of COVID-19 EV-induced NETs-associated proteins [e.g., myeloperoxidase (MPO), peptidylarginine deiminases 4 (PAD4), and citrullinated histone H3 (citH3)] were inhibited completely in TLR8 knockdown cells (Fig. 2E), which was consistent with IFA data.

Reactive oxygen species (ROS) are essential in the regulation of NETs formation [18]. Nicotinamide-adenine dinucleotide phosphate (NADPH) oxidase is crucial for the major production of ROS. We showed that COVID-19 patient–derived EV-induced NETs formation was inhibited in the presence of the NADPH oxidase inhibitor diphenyleneiodonium (DPI, 25 μM) (Fig. 2F). Moreover, increased cytosolic ROS levels were revealed in the neutrophils after being treated with COVID-19 patient–derived EVs (Fig. 2G) and this effect was inhibited in the presence of the NADPH oxidase inhibitor diphenyleneiodonium (DPI, 25 μM), TLR7-specific inhibitor IRS661 (10 μM), or TLR8-specific inhibitor Cu-CPT9a (10 μM). The inhibitory effect of TLR8 antagonists is better than that of TLR7 antagonists. Our results showed that COVID-19 patient–derived EVs induced NETs formation by activating TLR7/8 to promote NADPH oxidase-dependent ROS production.

### SARS-CoV-2 spike protein-triggered platelet–derived EVs enhanced virus-primed NETs formation by TLR7/8 activation

**Fig. 3:**
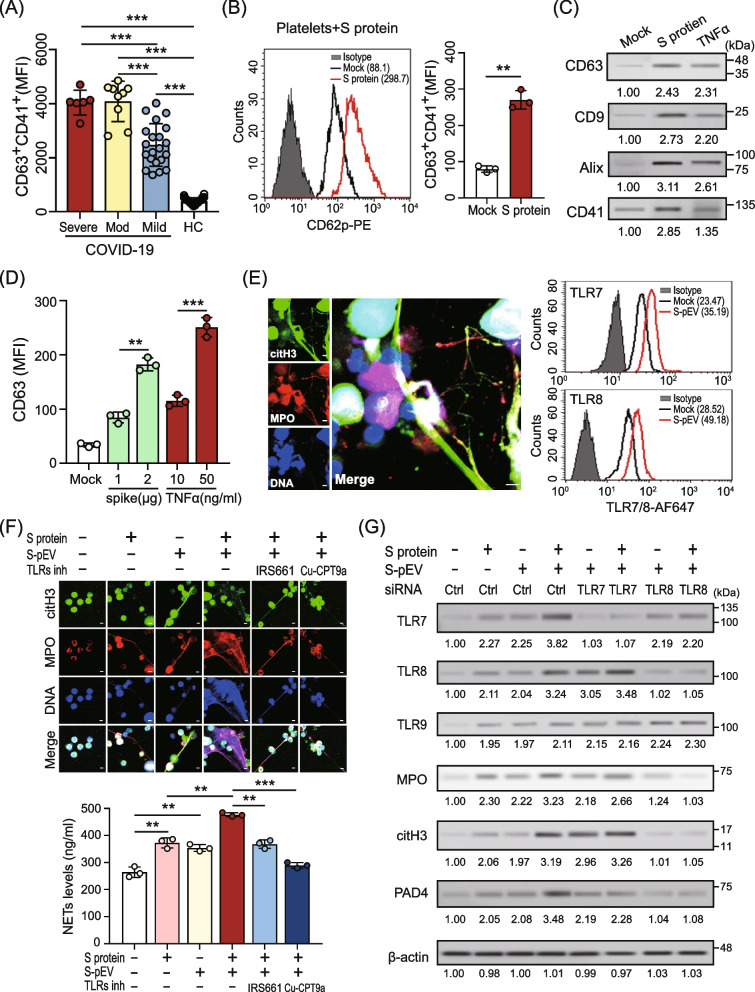
SARS-CoV-2 spike protein-triggered platelet–derived EVs enhanced virus-primed NETs formation by TLR7/8 activation. **A** Increased platelet–derived EVs (pEVs) were released in the plasma of patients with COVID-19 and are associated with disease severity. Mod, Moderate. **B** and **C** The human platelets were treated with viral spike protein (2 μg/ml) for 1 h, (**B**) the intracellular CD62p expression was analyzed and quantified using flow cytometry. **C** The levels of pEVs production were detected by immunoblotting. TNFα treatment was used as positive control. **D** Human platelets were treated with the indicated concentrations of spike protein or TNFα for 24 h. The pEVs were isolated and quantified with the CD63 antibody using flow cytometry. **E** Human neutrophils were treated with spike protein-triggered pEVs for 4 h, NETs formation was observed using confocal microscopy with the IFA assay (left panel) and intracellular TLR7/8 expression was analyzed using flow cytometry. **F** Human neutrophils were stimulated with the SARS-CoV-2 spike protein in the presence or absence of spike protein-triggered pEVs (S-pEV), TLR7-, or TLR8-specific inhibitors for 4 h; NETs formation was observed using confocal microscopy (upper panel) and quantified by the MPO-DNA PicoGreen assay (lower panel). **G** Human neutrophils were transfected with control siRNA, TLR7 siRNA, or TLR8 siRNA (30 nM) for 24 h. The SARS-CoV-2 spike protein in the absence or presence of S-pEVs was added to control cells, TLR7-, or TLR8-knockdown cells, respectively. After 24 h, the expression of intracellular TLR7/8/9 and NETs-associated proteins was analyzed by using immunoblotting. The scale bar in the IFA image represents 5 μm. All experiments were performed in triplicate and data were presented as the mean ± SD. **P < 0.01, ***P < 0.005

Platelets are the primary source of circulating EVs in the blood [19]. We observed that elevated platelet–derived EVs (pEVs) were shown in patients with COVID-19 and levels of pEVs are associated with severe disease (Fig. 3A). To validate the association between SARS-CoV-2 infection and elevated pEVs, human platelets were treated with viral spike protein (2 μg/ml) for 1 h; intracellular CD62p expression (activation marker of platelets) and the levels of pEVs production were analyzed and quantified by using flow cytometry and immunoblotting, respectively. The results showed that the SARS-CoV-2 spike protein could activate human platelets directly (MFI, 270.62 ± 25.17 versus 76.60 ± 8.82, *P* < 0.01, Fig. 3B) and was accompanied by increased pEVs release in a dose-dependent manner (Fig. 3C and D). In addition to the spike protein, TNFα has similar inductive effects on pEVs production (Fig. 3C and D). Moreover, we performed immunoblotting to confirm there is no spike protein present in spike protein-triggered pEVs (Supplementary Figure S1 (see Additional file 1)).

Next, we assessed the effects of spike protein-triggered pEVs on NETs formation. As shown in Fig. 3E, spike protein-triggered pEVs could induce NETs formation directly (left panel) and enhance TLR7/8 expression (right panel). We further assessed the effect of pEVs on SARS-CoV-2-primed NETs enhancement. A dramatically increased spike protein-primed NETs formation was shown in the presence of pEVs (Fig. 3F), which suggested that pEVs play a crucial role in hyperactive NETs formation. It should be noted that the effect of spike protein-triggered pEVs on NETs enhancement was almost completely suppressed in the presence of the TLR8-specific inhibitor Cu-CPT9a (Fig. 3F) or TLR8 knockdown cells (Supplementary Figure S2 (see Additional file 1), Fig. 3G). Our results suggested that spike protein-triggered pEVs may carry specific single-stranded RNAs (e.g., microRNAs) to activate TLR7/8 in neutrophils and the subsequent NETs formation. The contribution of TLR8 is greater than that of TLR7.

### Platelet–derived miR-21/let-7b induced NETs formation by EVs transmission

**Fig. 4:**
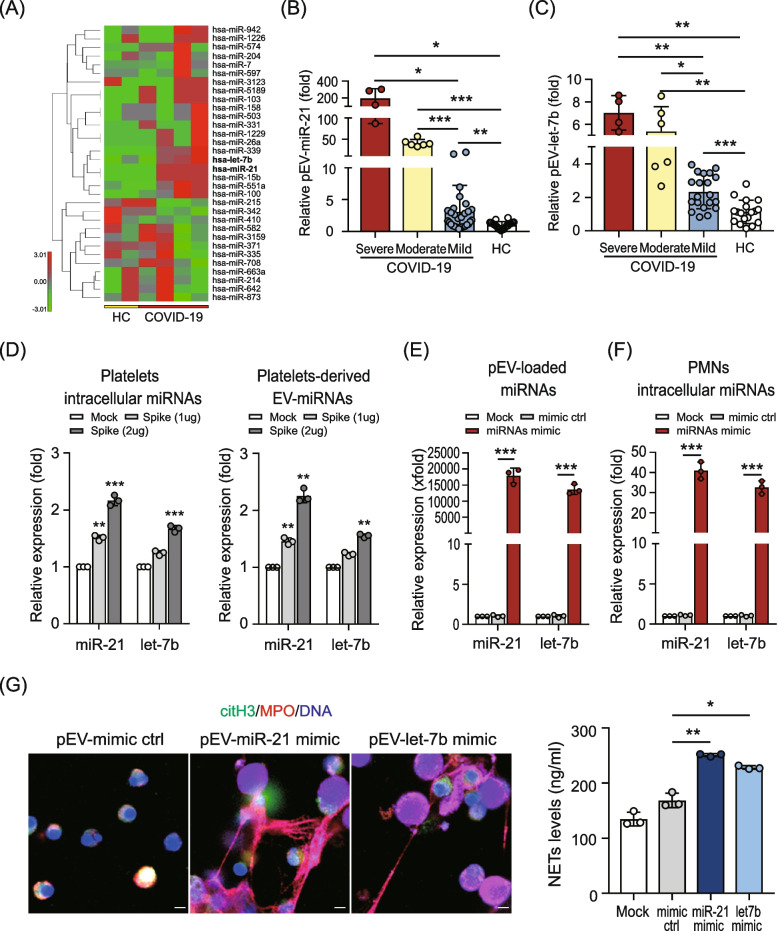
Platelet–derived miR-21/let-7b induced NETs formation by EVs transmission. **A** Hierarchical clustering of plasma–derived EV-carried microRNA (miRNA) profiles in COVID-19 patients and the healthy control (HC) group using next generation sequencing analysis. The red color represents a relative expression greater than the median expression level across all samples and the green color represents an expression level lower than the median. **B** and **C** Comparison of (**B**) exo-miR-21 and (**C**) exo-let-7b levels in COVID-19 patients and HC. **D** Human platelets were stimulated with the SARS-CoV-2 spike protein (2 μg/ml) for 1 h; the levels of intracellular miR21/let-7b (left panel) and platelet–derived EVs (pEVs)-carried miR21/let-7b (right panel) were analyzed using QRT-PCR, respectively. **E** The miR-21/let-7b mimic or mimic control (10 μM) were loaded into human pEVs using electroporation. The levels of miR-21/let-7b loaded were measured by QRT-PCR. **F** and **G** Human neutrophils were treated with pEVs that carried-different miRNAs for 24 h. **F** The intracellular miRNAs were analyzed using QRT-PCR. **G** NETs formation was observed using confocal microscopy (left panel) and quantified by the MPO-DNA PicoGreen assay (right panel). The scale bar in the IFA image represents 5 μm. All experiments were performed in triplicate and data were presented as mean ± SD. *P < 0.05, **P < 0.01, ***P < 0.005

Our results revealed that spike protein-triggered pEVs mainly induced NETs formation by TLR8 activation, suggesting that single stranded RNA (e.g., miRNAs) carried by pEVs might contribute to NETs formation. We compared the miRNA profiles in EVs from the plasma of COVID-19 patients and HC subjects by using a next-generation sequencing (NGS) approach. After normalization, we observed 32 miRNAs that were distinctively expressed in EVs from the plasma of COVID-19 patients (*n* = 4) compared to HC subjects (*n* = 2) (Fig. 4A, Supplementary Table S1 (see Additional file 1)). miR-21 and let-7b are abundantly expressed in platelets [20]; our NGS results showed that miR-21/let-7b was increased in the EVs of COVID-19 patients compared to those in HC subjects. We further validated their expression by using QRT-PCR. Elevated miR-21 (severe: 198.51 ± 111.60-fold; moderate: 41.07 ± 8.38-fold; mild: 3.10 ± 4.13-fold; HC: 1.00 ± 0.54-fold, *P* < 0.05, Fig. 4B) and let-7b (severe: 7.02 ± 1.54-fold; moderate: 5.37 ± 2.20-fold; mild: 2.33 ± 1.01-fold; HC: 1.14 ± 0.69-fold, *P* < 0.05, Fig. 4C) expression was revealed in the EVs of patients with COVID-19 and is associated with disease severity.

To confirm the association between the levels of miR-21/let-7b carried by pEVs and SARS-CoV-2 infection, human platelets were stimulated with the viral spike protein for 1 h; intracellular and pEVs-carried miR-21/let-7b expression was quantified using QRT-PCR. Elevated intracellular miR-21(2.17 ± 0.09-fold, *P* < 0.005)/let-7b (1.67 ± 0.06-fold, *P* < 0.005) was revealed in platelets after stimulating the spike protein (Fig. 4D, left panel). Moreover, increased levels of miR-21 (2.25 ± 0.12-fold, *P* < 0.01)/let-7b (1.55 ± 0.03-fold, *P* < 0.01) were shown in pEVs released from platelets with spike protein treatment, compared to those derived from control cells (1.00-fold) (Fig. 4D, right panel). The levels of intracellular- or pEVs-carried miR21/let7b induced by the spike protein were dose-dependent. To assess the effect of pEVs-carried miR-21/let-7b on NETs formation, miR-21/let-7b mimics or control mimics were loaded into human pEVs by using electroporation in accordance with our previously published report [21]. The levels of encapsulated miR-21/let-7b were measured using QRT-PCR. The increased expression of miR-21 (1.8 ± 0.2 × 10^4^-fold)/let-7b (1.4 ± 0.2 × 10^4^-fold) was observed in human pEVs after electroporation with miR-21/let-7b mimic, indicating its effective loading by electroporation (Fig. 4E). The miR-21/let-7b-loaded pEVs were then added to human neutrophils. After 24 h, significantly increased intracellular miR-21/let-7b levels (Fig. 4F), accompanied by elevated NETs formation (Fig. 4G), were observed in neutrophils with the addition of miR-21/let-7b mimic-loaded pEVs.

### pEVs-carried miR-21/let-7b enhanced NETs formation by TLR7/8 activation

**Fig. 5:**
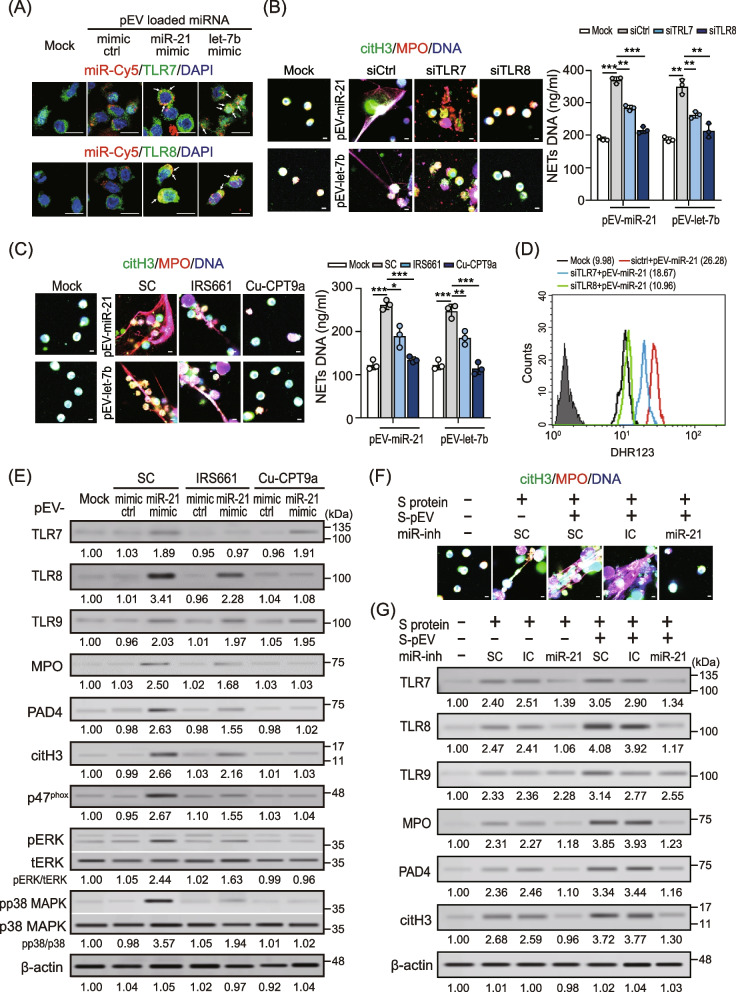
pEVs-carried miR-21/let-7b enhanced NETs formation by TLR7/8 activation. **A** Human platelet–derived EVs were loaded with Cy5-labeled miR-21/let-7b mimic (red) or mimic control (red) and then co-cultured with dHL-60 cells for 4 h. Endogenous TLR7 or TLR8 was stained with TLR7 or TLR8 antibodies (green), respectively. The miR-21/let-7b transmission and endogenous TLR7/8 in dHL-60 cells was detected by using the immunofluorescence assay. The scale bar in the image represents 10 μm. **B** Human neutrophils were transfected with control siRNA, TLR7, or TLR8 siRNA (30 nM) for 24 h; MiR-21/let-7b mimic-loaded pEVs were added to control cells, TLR7-, or TLR8-knockdown cells, respectively. After 24 h, NETs formation was observed using confocal microscopy (left panel) and quantified by MPO-DNA PicoGreen assay (right panel). **C** Human neutrophils were treated with IRS661 (TLR7 specific inhibitor), or Cu-CPT9a (TLR8-specific inhibitor) for 4 h. PBS was used as solvent control (SC). MiR-21/let-7b mimic-loaded pEVs were added to individual treating cells, respectively. After 24 h, NETs formation was observed using confocal microscopy (left panel) and quantified by MPO-DNA PicoGreen assay (right panel). **D** MiR-21 mimic-loaded pEVs were added to control cells, TLR7-, or TLR8-knockdown cells, respectively. The levels of cytosolic ROS in were detected using flow cytometry with dihydrorhodamine (DHR) 123 staining. **E** Human neutrophils were treated with miR-21 mimic-loaded pEVs in the presence of TLR7/8-specific inhibitors for 24 h. The expression of intracellular TLR7/8/9 and NETs-associated proteins was analyzed by using immunoblotting. **F** and **G** Human neutrophils were treated with SARS-CoV-2 spike protein (S protein) or/and spike protein-primed platelets–derived EVs (S-pEVs) in the presence of a miRNA inhibitor control or an miR-21 inhibitor for 24 h. **F** NETs formation was observed using confocal microscopy. **G** The expression of intracellular TLR7/8/9 and NETs-associated proteins was analyzed using immunoblotting. Immunoblotting bands from β-actin were densitometrically measured by ImageJ to determine the lane normalization factor for samples. The scale bar in the IFA image represents 5 μm. The image shown is from a single experiment that is representative of at least three separate experiments. Data are presented as the mean ± SD. *P < 0.05, **P < 0.01, ***P < 0.005. SC, solvent control. IC, microRNA inhibitor control

To assess the effect of TLR7/8 on pEVs-carried miR-21/let-7b induced NETs formation, the levels of TLR7/8 in neutrophils were analyzed using flow cytometry. Increased TLR7 and TLR8 expression were both induced in neutrophils in the presence of miR-21/let-7b-loaded pEVs (Supplementary Figure S3 (see Additional file 1)). We further examined whether platelet–derived miR-21/let-7b could bind to TLR7/8 on neutrophils through pEVs transmission using the immunofluorescence assay. The pEVs were loaded with Cy5-labeled miR-21/let-7b mimic or mimic control and then co-cultured with the differentiated HL-60 human neutrophil-like (dHL-60) cells for 4 h; the miR-21/let-7b transmission and endogenous TLR7/8 in dHL60 cells was detected using the immunofluorescence assay. As shown in Fig. 5A, the colocalization of miR-21/let-7b and TLR7/8 were revealed in dHL60 cells, respectively. This suggested that miR-21 or let-7b may bind to TLR7 and TLR8. Further experiments are needed to confirmed our observation. We further checked the effects of TLR7/8 on miR-21/let-7b-loaded pEVs-induced NETs formation by using TLR7/8 knockdown cells (Fig. 5B) and TLR7/8 specific inhibitor treatment (Fig. 5C), respectively. The results showed that the levels of miR-21/let-7b-loaded pEVs-induced NETs formation was partially reduced in TLR7 knockdown cells (miR-21: 370.13 ± 10.04 ng/ml versus 284.23 ± 8.01 ng/ml,* P* < 0.01; let-7b: 350.24 ± 22.54 ng/ml versus 263.03 ± 10.16 ng/ml, *P* < 0.01, Fig. 5B), but was almost completely suppressed in TLR8 knockdown cells (miR-21: 215.96 ± 10.36 ng/ml, *P* < 0.005; let-7b: 213.67 ± 21.73 ng/ml, *P* < 0.01). A similar phenomenon was shown in cells with TLR7/8 antagonist treatment (Fig. 5C). Given that a dramatically increased miR-21 were revealed in pEVs of severe/moderate COVID-19 patients (Fig. 4B), we focused on the miR-21. We showed that miR-21-loaded pEVs could induce ROS production dramatically (Fig. 5D). This effect was almost disappeared completely in TLR8 knockdown cells, but was only slightly decreased in TLR7 knockdown cells.

Given that we showed COVID-19 patient–derived EV-induced NETs formation by enhancing NADPH oxidase-dependent ROS production (Fig. 2G). Previous studies demonstrated that the phosphorylation of p47phox is regulated by ERK (extracellular-signal-regulated kinase) and p38 MAPK (mitogen-activated protein kinase), which is crucial in ROS production [22]. We further assessed whether the miR-21-TLR8 axis was involved in p47phox phosphorylation to modulate ROS production. Human neutrophils were treated with miR-21-loaded pEVs in the presence of the TLR8-specific inhibitor Cu-CPT9a or the TLR7-specific inhibitor IRS661, respectively. As shown in Fig. 5E, increased levels of ERK activation were revealed in cells with miR-21-loaded pEVs treatment, accompanied by elevated p47phox phosphorylation and the enhancement of NETs-associated proteins. These effects were inhibited completely in the presence of Cu-CPT9a, but there was only a slight decrease in the presence of IRS661. Our results revealed that platelet–derived miR-21 interact with TLR7/8 of neutrophils through pEVs transmission, resulting in ERK/p38 MAPK activation, and p47phox phosphorylation to promote ROS production and the enhancement of NETs formation. The effects of miR-21 mimic-loaded pEVs on TLR7/8 activation and NETs formation were suppressed in the presence of miR-21 inhibitors (Supplementary Figure S4 (see Additional file 1)) compared with those in inhibitor control cells. In addition, no matter SARS-CoV-2 spike protein-induced NETs formation or the effect of pEVs on SARS-CoV-2 spike protein-primed NETs enhancement was inhibited significantly in the presence of miR-21 inhibitors (Fig. 5F, Fig. 5G, and Supplementary Figure S5 (see Additional file 1)).

### SARS-CoV-2-primed pEVs induced IL-1β, TNF-α and IL-8 upregulation in the neutrophils through TLR7/8 and NF-κB activation

**Fig. 6:**
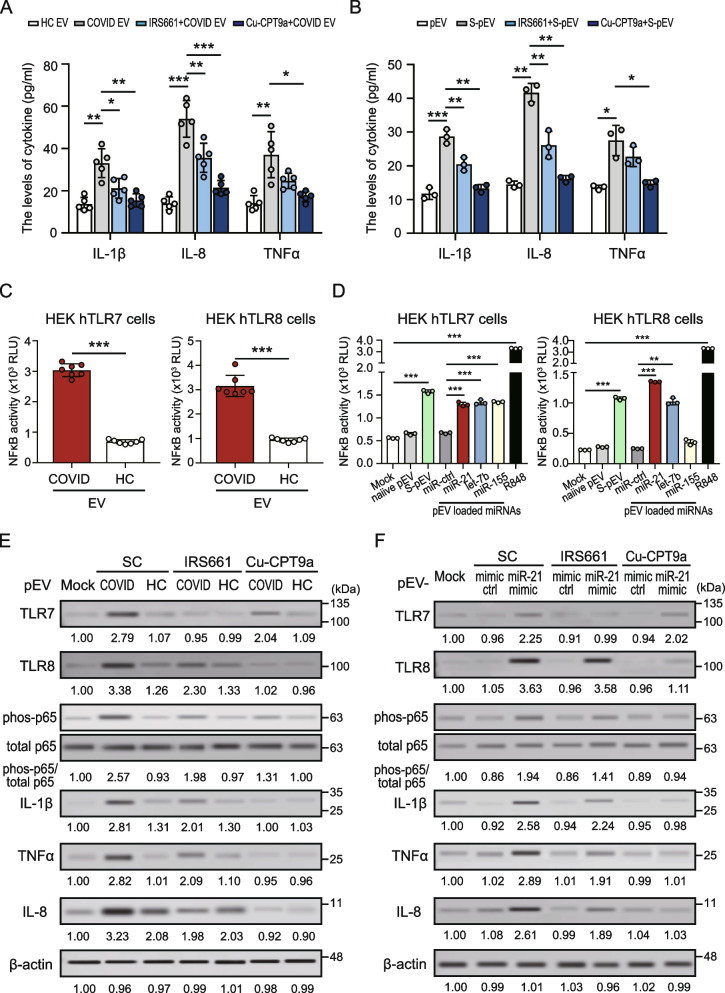
COVID-19–derived pEVs induced IL-1β/TNF-α and IL-8 upregulation in neutrophils through TLR8 and NF-κB activation. **A** and **B** Human neutrophils were treated with (**A**) plasma–derived pEVs or (**B**) SARS-CoV-2 spike protein-primed pEVs in the presence of a TLR7/8 specific inhibitor for 24 h. The supernatant was collected. The levels of IL-1β, TNF-α and IL-8 were measured by using ELISA. **C** and **D** The HEK-Blue hTLR7 (left panel) and HEK-Blue hTLR8 (right panel) cells were stimulated with (**C**) plasma–derived EVs from the indicated individuals or (**D**) indicated miRNA-loaded pEVs for 24 h. NF-κB activation was evaluated in terms of luciferase activity compared to control cells. **E** and **F** Human neutrophils were treated with (**E**) plasma–derived EVs or (**F**) miR-21-loaded pEVs in the presence of TLR7/8 specific inhibitors for 24 h. The intracellular TLR7/8, phosphorylation of p65 subunits and proinflammatory cytokines/chemokines (IL-1β/TNF-α and IL-8) were analyzed using immunoblotting. Immunoblotting bands from β-actin were densitometrically measured by ImageJ to determine the lane normalization factor for samples. All experiments were performed in triplicate and data are presented as the mean ± SD. The image shown is from a single experiment that is representative of at least three separate experiments. *P < 0.05, ** P < 0.01, *** P < 0.005. HC, healthy control

Accumulating evidence showed that increased IL-1β/TNF-α/IL-8 in the serum of patients with COVID-19, compared to those in HC subjects [3, 23]. We examined whether COVID-19-associated pEVs could modulate IL-1β/TNF-α/IL-8 expression in neutrophils. Human neutrophils were treated with plasma–derived EVs from COVID-19 patients or HC subjects for 24 h; the supernatants were collected and the levels of IL-1β/TNF-α/IL-8 were analyzed using ELISA. Increased levels of IL-1β/TNF-α/IL-8 were released from human neutrophils after being stimulated with COVID-19 patient–derived EVs, compared to those in control cells (Fig. 6A). Similar effects were also observed in viral spike protein-primed pEVs-treated cells (Fig. 6B); these effects were suppressed in the presence of TLR7/8 inhibitors, particularly in those with TLR8-specific inhibitor treatment.

To dissect the regulatory mechanism of COVID-19 patient–derived EV-induced proinflammatory cytokines/chemokines upregulation, we used the HEK-hTLR7/8 cell model (Invivogen, USA) to examine COVID-19 patient–derived EVs and miR21/let-7b-loaded pEVs modulation on NF-κB activity upon TLR7/8 engagement. As shown in Fig. 6C, increased NF-κB activity was induced in TLR7/8-expressing cells stimulated with COVID-19 patient–derived EVs, compared to those treated with HC subject–derived EVs (*P* < 0.005). Moreover, elevated NF-κB activity was also induced in TLR7/8-expressing cells stimulated with SARS-CoV-2 spike protein-primed pEVs, or miR-21/let-7b-loaded pEVs compared with control cells (*P* < 0.005, Fig. 6D). We further showed the significantly increased phosphorylation of the p65 subunits of NF-κB in cells after stimulating with COVID-19 patient–derived EVs, compared with those from healthy control subjects (2.77 ± 0.06-fold versus 1.01 ± 0.13-fold, *P* < 0.005, Fig. 6E), accompanied by elevated proinflammatory cytokine/chemokine (e.g., IL-1β, TNF-α and IL-8) levels. Consistent with this, SARS-CoV-2 spike protein-primed pEVs (Supplementary Figure S6 (see Additional file 1)) or miR-21-loaded pEVs (Fig. 6F) also had the same effects on the phosphorylation of p65 subunits, resulting in elevated proinflammatory cytokine levels. These effects were completely inhibited in the presence of the TLR8 inhibitor Cu-CPT9a.

## Discussion

Neutrophils have a variety of important biological functions in both innate and adaptive immunities, thus playing a key role in infection. The release of NETs was attributed to the capture and elimination of pathogens [24]. However, excessive NETs formation caused harmful coagulopathy and immunothrombosis in severe COVID-19 cases [7–9]. Decreased or inhibited NETs formation may reduce NETs-mediated inflammatory and thrombotic tissue damage associated with severe COVID-19 [7–9]. Therefore, it is crucial to understand how SARS-CoV-2 induces hyperactive NETs formation so that alternative therapeutic strategies can be developed.

Veras et al. demonstrated that SARS-CoV-2 induced NETs formation directly, which depended on angiotensin converting enzyme 2 (ACE2), serine protease, and PAD-4 in neutrophils [8]. A previous study demonstrated that low-density granulocytes (LDGs), a distinct pro-inflammatory neutrophil subset, exhibit enhanced NETs production in individuals with autoimmune disease [18]. Accumulating evidence found that the amounts of LDGs are upregulated in patients with severe COVID-19 and a poor clinical prognosis [25, 26]. In addition to LDGs, activated platelets have been shown to enhance NETs formation by interacting with neutrophils through toll-like receptor 4 (TLR4), platelet factor 4 (PF4) and EV-dependent processes [27, 28]. Garnier et al. observed that intubated COVID-19 patient–derived EVs cause NETosis and endothelial cell death, but the regulatory mechanism responsible for this is unclear [11]. Herein, we observed that circulating NETs DNA and EVs were both increased in the plasma of patients with COVID-19 and associated with disease severity. We showed that platelets are a major source of EVs from COVID-19 patients, which is consistent with a previous report [15]. Activated-platelets increased pEVs release [29]. Florian et al. speculated that the direct interaction with SARS-CoV-2, or excessive inflammatory responses and tissue damage may stimulate platelets to release pEVs [30]. We showed that the SARS-CoV-2 spike protein directly induced platelet activation and increased pEVs release in a dose-dependent manner. Moreover, we observed that spike protein-induced NETs formation was dramatically enhanced in the presence of spike protein-primed pEVs, suggesting that pEVs play a crucial role in hyperactive NETs formation. Although SARS-CoV-2-associated pEVs could activate both TLR7 and TLR8 in neutrophils, we showed that the pEVs-induced NETs enhancement or proinflammatory cytokine upregulation were only suppressed completely in cells with TLR8-specific inhibitor treatment, but the same was not shown for TLR7 antagonists. We confirmed that COVID-19 patient–derived pEVs and viral spike protein-primed pEVs induced NETs formation in a TLR8-dependent manner using TLR8 knockdown cells. Human neutrophils express more TLR8 than TLR7 [22] and we observed that elevated levels of TLR8 were higher than those of TLR7 in neutrophils after pEVs stimulation. In addition, we demonstrated that COVID-19 patient–derived pEVs-enhanced NETs formation is NADPH oxidase-dependent. Karama et al. demonstrated that TLR8, but not TLR7, induces the priming of NADPH oxidase activation in human neutrophils [22]. Increasing evidence showed that hyperactive neutrophils are associated with severe COVID-19 [7–9]; therefore, TLR8 may be a potential therapeutic target for severe COVID-19. More experiments are required to confirm our results.

The EVs carried diverse materials (e.g., RNA, lipids and proteins) that were transferred to different cellular recipients, which facilitates communication between cells and influences the recipient cell function [31]. Given our observation that SARS-CoV-2-primed pEVs-induced NETs formation is through uptake followed by TLR7/8 activation, this suggested that single-stranded RNA (e.g., microRNAs) carried by pEVs might contribute to NETs formation. MicroRNAs (miRNAs) are endogenous, noncoding RNAs that mediate mRNA cleavage, translational repression, or mRNA destabilization [32]. Increasing evidence has demonstrated that miRNAs exert biological functions by EVs transmission to modulate the host immune response in viral infection [33, 34]. Zhang et al. showed that the SARS-CoV-2 spike protein induced platelet activation by ACE2 binding to enhance thrombosis [35]. miR-21 and let-7b are abundant in platelets [20]. Increased levels of circulating miR-21 [36, 37] and let-7b [38] have been observed in severe COVID-19 and acute SARS-CoV-2 infection, respectively. In this study, we detected elevated levels of miR-21/let-7b loaded in EVs from the plasma of patients with SARS-CoV-2 infection. We demonstrated that the SARS-CoV-2 spike protein could activate platelets and enhance intracellular and pEVs-carried miR-21/let-7b levels in a dose-dependent manner. The levels of miR-21/let-7b carried by EVs are associated with disease severity. Based on our results, the levels of pEVs-carrying miR-21/let-7b may be potential predisposing factors for the development of severe COVID-19. However, more in-depth experiments are required to confirm our observation.

miR-21 and let-7b are GU-enriched miRNAs [20]. Accumulating studies have described GU-rich elements, or the number of U ribonucleotides in the sequence composition, to be crucial for innate immune activation by single-stranded RNAs [39, 40]. Previous studies showed that the let-7 family [41] and miR-21 [42] were TLR7 and TLR8 signaling activators, respectively. However, their roles in NETs formation remain elusive. In this study, we showed that pEVs carrying miR-21/let-7b induced NETs formation by TLR7/8 activation in neutrophils; most of the effects were contributed by TLR8. We showed that miR-21/let-7b interacts with TLR7/8 and promotes ROS production in neutrophils. Given that the levels of TLR8 expression were significantly higher than TLR7 in neutrophils and ROS production is TLR8-dependent [22], miR-21/let-7b-induced NETs formation was almost completely suppressed by using the TLR8 inhibitor. The levels of NETs formation induced by miR-21 are greater than those for let-7b. In addition, we showed that the miR-21 inhibitor could suppress spike protein-induced NETs formation and pEVs-primed NETs enhancement. Our data suggested that miR-21 inhibitors could be potential therapeutic candidates for SARS-CoV-2 infection, although more in-depth in vivo experiments are needed to validate our results.

ROS are essential components for NETs formation. We showed that NADPH oxidase inhibitors could completely suppress pEVs-induced NETs enhancement. Saitoh et al. showed that the engagement of TLR7/8 induces the production of ROS, thus triggering NETs formation [43]. Giraldo et al. revealed that HIV-1–derived GU-rich single-stranded RNA40 (ssRNA40) activates neutrophils based on TLR7/8 and ROS production [44]. Karama et al. [22] demonstrated that the TLR7/8 agonist primes N-formyl-methionyl-leucyl-phenylalanine-stimulated NADPH oxidase activation in human neutrophils through p47phox phosphorylation. We showed that COVID-19 patient–derived EVs or viral spike protein triggered-pEVs that carried GU-enriched miRNAs (e.g., miR-21/let-7b) interacted with TLR7/8 to induce p47phox phosphorylation, activate NADPH oxidase and thus promote ROS production in neutrophils to enhance NETs formation. TLR8 was shown to contribute ROS production more than TLR7.

Dysregulated proinflammatory cytokine production is a key characteristic of patients with severe COVID-19 [45]. Keshari showed that proinflammatory cytokines (e.g., IL-1β, IL-8 and TNFα) enhanced NETs formation through NADPH oxidase activation and MPO mediation [46]. Increased levels of the neutrophil-associated cytokine interleukin 8 (IL-8) were detected in patients with COVID-19, particularly in those who were critically ill [23, 47]. Therefore, IL-8 has been suggested to be a biomarker of disease prognosis [23, 47]. Kaiser et al. identified a positive feedback loop of autocrine IL-8 dysregulation, resulting in prothrombotic neutrophil activation and NETs formation [48]. They show that blocking IL-8-like signaling reduces SARS-CoV-2 spike protein-induced acute respiratory distress syndrome (ARDS) and human ACE2-dependent pulmonary microthrombosis in mice. A previous study showed that TLR8 activation could stimulate the massive production of IL-8, which was largely determined by post-transcriptional regulation [49]. Recently, Chen et al. showed that COVID‑19 plasma–derived EVs could promote proinflammatory cytokine (e.g., IL-1β, IL-8, TNF-α) production in peripheral blood mononuclear cells, but the regulatory mechanism remains elusive [50]. Herein, we demonstrated that COVID-19 patient–derived EVs or viral spike protein triggered-pEVs that carried miR-21/let-7b induced IL-1β/TNF-α/IL-8 upregulation in neutrophils by interacting with TLR7/8 to activate NF-κB. Upregulated proinflammatory cytokines (e.g., IL-1β/TNF-α/IL-8) could induce NETs formation [46] and promote platelet activation [51]. In addition to viral spike protein, we showed that TNF-α could induce pEVs release. We hypothesized that these pEVs-primed autocrine proinflammatory cytokines may play crucial roles in NETs enhancement. Further ex vivo studies are required to confirm our hypothesis.

Despite the novel findings presented in this pilot study, there are some limitations. Given that the number of severe COVID-19 patients enrolled in our study was quite limited, the number of severe COVID-19 subjects was small. Additionally, the study was cross-sectional by design and, thus, the possibility that NETs formation/EVs production/miRNA expression changed with therapeutic strategies cannot be excluded. Finally, the limitations of biosafety criteria and space in our institute meant that we were not able to perform animal experiments to validate our results. However, we validated our observations using an in vitro cell-based assay and clinical specimens from COVID-19 patients. Therefore, our results still provide valuable information.

**Fig. 7:**
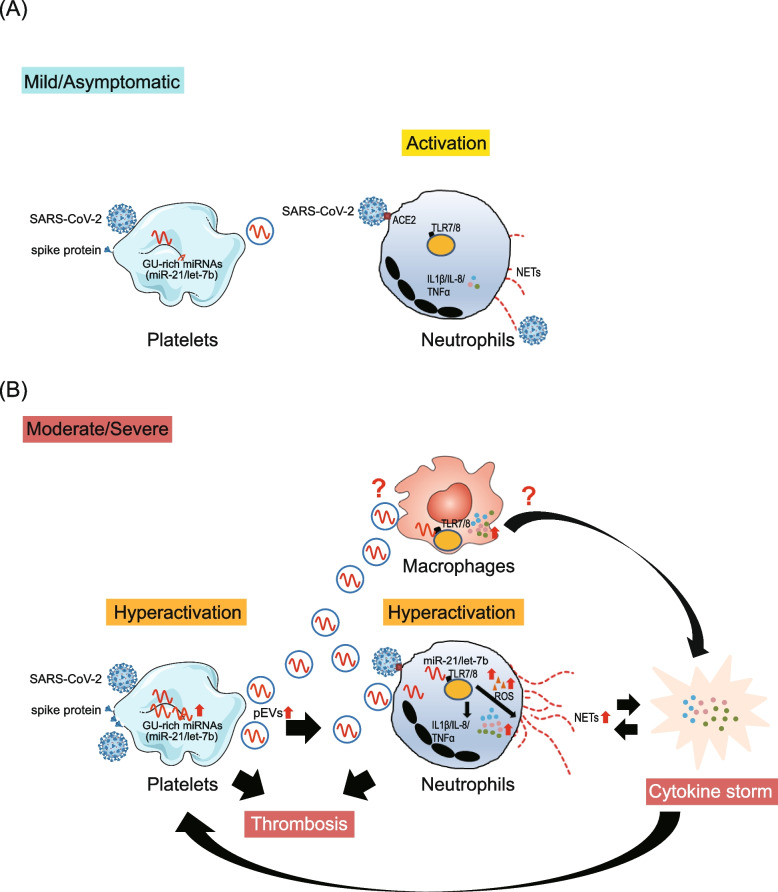
Proposed model for the biological role of platelet–derived miR-21, TLR7/8 and NETs formation in (**A**) mild COVID-19 and (**B**) severe COVID-19, based on the results of this study. **A** SARS-CoV-2 activates neutrophils and induces NETs formation during infection. **B** In severe COVID-19 cases with platelets hyperactivation, endogenous GU-enriched miRNAs (e.g., miR-21 and let-7b) were upregulated and EVs were increased. The pEVs-carried miR-21/let-7b interacts with TLR7/8 to (1) promote ROS production, thus enhancing SARS-CoV-2 primed NETs formation and (2) activate NF-κB, thus upregulating proinflammatory cytokines/chemokines (IL-1β/TNF-α and IL-8). Increased proinflammatory cytokines/chemokines (e.g., TNF-α) could enhance platelet activation via positive feedback

Neutrophils are the first line of defense against infection. In mild infection, neutrophils release NETs to contribute to pathogen clearance (Fig. 7A). Accumulating studies found that blood-borne EVs carry potential as biomarkers of COVID-19 disease severity and as predictors of outcome [30]. Increased platelet activation and hyperreactivity are implicated in the severity and mortality of COVID-19 [52, 53]. We demonstrated that the SARS-CoV-2 spike protein induced platelet activation, resulting in miR-21/let7b upregulation and which was carried by pEVs. Upregulated pEVs-carried miR-21/let-7b could interact with TLR7/8 in neutrophils to activate NADPH oxidase 2 (NOX2) and promote ROS production, causing the enhancement of NETs formation (Fig. 7B). In addition, miR-21 also interacts with TLR7/8 to modulate NF-κB activity and induce the over-expression of proinflammatory cytokines/chemokines. The dysregulated proinflammatory cytokines/chemokines may cause prothrombotic platelet/neutrophil activation and feedback to enhance NETs formation. Host-directed therapies are a relatively new and promising approach for the treatment of COVID-19 [54]. EVs are important mediators of intercellular communication to regulate a diverse range of biological processes, highlighting their potential as novel targets for therapeutic intervention [55].

## Conclusions

This study offers new molecular machinery to explain the association between platelets–derived miRNAs, NETs formation and SARS-CoV-2 infection. We showed that TLR8 antagonists, NOX2 suppressors, or miR-21 inhibitors effectively suppressed pEVs-induced NETs enhancement and IL-1β/IL-8/TNFα production in neutrophils. Our results suggested that the miR-21-TLR8 axis may be a potential therapeutic strategy against severe COVID-19.

### Supplementary Information

**Additional file 1. **

**Additional file 2. **

**Additional file 3. **

## Data Availability

All data generated or analyzed during this study are included in this published article and its supplementary information files. Raw data generated by miRNA sequencing have been deposited to National Center for Biotechnology Information (NCBI) Sequence Read Archive (SRA) under the accession code PRJNA1010965 (https://www.ncbi.nlm.nih.gov/sra/PRJNA1010965).

## Abbreviations

ACE2: Angiotensin converting enzyme 2
ANOVA: A one-way analysis of variance
BafA1: Bafilomycin A1
citH3: Citrullinated histone H3
COVID-19: Coronavirus disease-19
DPI: Diphenyleneiodonium
ERK: Extracellular-signal-regulated kinase
EVs: Extracellular vesicles
HBSS: Hank's balanced salt solution
HC: Healthy control
ICU: Intensive care unit
IFA: Immunofluorescence assay
LDGs: Low-density granulocytes
MAPK: Mitogen-activated protein kinase
MFI: Mean fluorescence intensity
miR: MicroRNA
MPO: Myeloperoxidase
NADPH: Nicotinamide-adenine dinucleotide phosphate
NETs: Neutrophil extracellular traps
NGS: Next-generation sequencing
NOX2: NADPH oxidase 2
pEVs: Platelet–derived EVs
PAD4: Peptidylarginine deiminases 4
PF4: Platelet factor 4
PMN: Polymorphonuclear leukocyte
QRT-PCR: Quantitative reverse transcription PCR
ROS: Reactive oxygen species
SARS-CoV-2: Severe acute respiratory syndrome coronavirus 2
TLR: Toll-like receptor
