# Supplementary material for: SARS-CoV-2 primed platelets–derived microRNAs enhance NETs formation by extracellular vesicle transmission and TLR7/8 activation
Source: Cell Commun Signal. 2023 Oct 30;21:304. doi: 10.1186/s12964-023-01345-4 (PMC10614402; doi:10.1186/s12964-023-01345-4)
Supplement: Supplementary file 2 — Additional file 1. [file 12964_2023_1345_MOESM1_ESM.pdf]

Supplementary information

SARS-CoV-2 primed platelets–derived microRNAs enhance NETs formation by extracellular vesicle transmission and TLR7/8 activation

Tsai-Ling Liao[1-3*], Hung-Jen Liu[2-5], Der-Yuan Chen[6-9], Kuo-Tung Tang[10], Yi-Ming Chen[1,2,3,10], Po-Yu Liu[2,3,11*]

[1]Department of Medical Research, Taichung Veterans General Hospital, Taichung 407, Taiwan

[2]Rong Hsing Research Center for Translational Medicine, National Chung Hsing University, Taichung 402, Taiwan

[3]Ph.D. Program in Translational Medicine, National Chung Hsing University, Taichung 402, Taiwan

[4]Institute of Molecular Biology, National Chung Hsing University, Taichung 402, Taiwan

[5]The iEGG and Animal Biotechnology Center, National Chung Hsing University, Taichung 402, Taiwan

[6]Rheumatology and Immunology Center, China Medical University Hospital, Taichung 404, Taiwan

[7]Translational Medicine Laboratory, Rheumatology and Immunology Center, China Medical University Hospital, Taichung 404, Taiwan

[8]College of Medicine, China Medical University, Taichung 404, Taiwan

[9]Institute of Medicine, Chung Shan Medical University Hospital, Taichung 402, Taiwan

[10]Division of Allergy, Immunology and Rheumatology, Taichung Veterans General Hospital, Taichung 407, Taiwa

[11]Division of Infection, Department of Internal Medicine, Taichung Veterans General Hospital, Taichung, Taiwan

Corresponding author and address reprint requests:

Dr. Tsai-Ling Liao, Department of Medical Research, Taichung Veterans General Hospital, Taichung, Taiwan

Address: No.1650, Sec.4, Taiwan Boulevard, Xitun Dist., Taichung City 40705, Taiwan

Tel.: 886-4-23592525, extension 4020; Fax: 886-4-23592705

Email: tlliao@vghtc.gov.tw

Supplementary Materials

Reagent or Resource	Source	Identifier (Cat No.)
Antibodies		
Mouse anti-Alix antibody	Santa Cruz	sc-53540
Mouse anti-CD9 antibodies	Abcam	ab58989
Mouse anti-CD63 antibodies	Abcam	ab68418
Mouse anti-CD81 antibodies	Abcam	ab79559
Mouse anti-citH3 antibodies	Abcam	ab5103
Rabbit anti-Erk1/2 antibodies	Cell Signaling Technology	#4695
Mouse anti-IL-1β antibodies	Santa Cruz	sc-52012
Mouse anti-IL-8 antibodies	Santa Cruz	sc-8427
Mouse anti-MPO/Myeloperoxidase antibodies (WB)	Santa Cruz	sc-52707
Mouse anti-MPO/Myeloperoxidase antibodies (ELISA)	Bio-Rad	MCA1757
Mouse anti-NFκB p65 antibodies	Santa Cruz	sc-8008
Mouse anti-p-NFκB p65 antibodies	Santa Cruz	sc-136548
Rabbit anti-p38 MAPK Antibody	Cell Signaling Technology	#9212
Rabbit anti-phospho p38 MAPK Antibody	Cell Signaling Technology	#9211
Mouse anti-p47phox antibodies	Santa Cruz	sc-17845
Rabbit anti-SARS-CoV-2 spike protein	Cell Signaling Technology	#99423
Mouse anti-TLR7 antibodies	Santa Cruz	sc-57463
Mouse anti-TLR8 antibodies	Santa Cruz	sc-373760
Mouse anti-TNFα antibodies	Santa Cruz	sc-52746
Mouse anti-TSG101 antibodies	Santa Cruz	sc-7964
Rabbit anti-CD41 antibodies	Cell Signaling	#13807

	Technology	
Rabbit anti-PAD4 antibodies	Abcam	ab96758
Rabbit anti-TLR7 antibodies	Novus Biologicals	NBP2-24906
Rabbit anti-TLR8 antibodies	Abcam	ab24185
Cell lines		
HEK-Blue^{TM} hTLR7 cells	InvivoGen	hkb-htlr7
HEK-Blue^{TM} hTLR8 cells	InvivoGen	hkb-htlr8
Chemicals and Assay kits		
Apyrase	Sigma-Aldrich	A6535
Bafilomycin A1	Sigma-Aldrich	B1793
CellTiter 96® AQueous One Solution Cell Proliferation Assay	Promega	G3580
Citrate-dextrose solution	Sigma-Aldrich	C3821
Cytochalasin D	Sigma-Aldrich	C8273
CU-CPT9a	InvivoGen	inh-cc9a
Dihydrorhodamine 123	Thermo Fisher Scientific	D23806
Diphenyleneiodonium chloride	Sigma-Aldrich	D2926
Hoechst 33342	Thermo Fisher Scientific	H3570
Polymorphprep	Axis-Shield	1895
Prostaglandin I2	Sigma-Aldrich	P6188
Tyrode's buffer	Sigma-Aldrich	T2397
Resiquimod	Sigma-Aldrich	SI-SML0196
SYTOX Green	Thermo Fisher Scientific	S7020
ExoQuick exosome precipitation solution	System Biosciences	EXOQ5A-1S
ExoQuick-TC	System Biosciences	EXOTC10A-1
Ficoll®-Paque Premium	GE Healthcare Biosciences	GE17-5442-02
QIAamp DNA Blood Mini Kit	QIAGEN	51106
Recombinant COVID 19 Spike Protein	MyBioSource	MBS434283
RNeasy MinElute® Cleanup Kit	QIAGEN	74204

Total Exosome RNA & Protein Isolation Kit	Thermo Fisher Scientific	4478545
Trizol	Thermo Fisher Scientific	15596018
hsa-miR-21 miRNA mimic	Thermo Fisher Scientific	MC10206
hsa-miR-21 miRNA inhibitor	Thermo Fisher Scientific	MH10206
hsa-let-7b miRNA mimic	Thermo Fisher Scientific	MC11050
mirVana miRNA mimic Negative Control	Thermo Fisher Scientific	4464058
mirVana miRNA inhibitor Negative Control	Thermo Fisher Scientific	4464076
hsa-miR-21 TaqMan MicroRNA Assays	Thermo Fisher Scientific	000397
hsa-let-7b TaqMan MicroRNA Assays	Thermo Fisher Scientific	002619
On TARGETplus SMARTpool siTLR7	Dharmacon	L-004714-00-0005
On TARGETplus SMARTpool siTLR8	Dharmacon	L-004715-00-0005
Human IL-1 beta/IL-1F2 Quantikine ELISA Kit	R&D	DLB50
Human IL-8 Quantikine ELISA Kit	R&D	D8000C
ExoELISA-ULTRA Complete Kit	System Biosciences	EXEL-ULTRA-CD63-1

Supplementary Methods

MicroRNA next generation sequencing (NGS) analysis

The small RNA library was prepared using a Total RNA-Seq kit v2.0 (Thermo Fisher Scientific, USA). Template preparation was carried out with the Ion PGM Template OT2 200 kit (Thermo Fisher Scientific, USA), according to the manufacturer's protocol; the Ion PGM^{TM} Sequencing 200 kit (Thermo Fisher Scientific, USA) and 318 chip were used with the Ion PGM sequencer, as described in the Ion PGM^{TM} Sequencing Kit User Guide. Data alignment to the hg19 human reference genome and base calling were done using the built-in Torrent Suite software v4.0 (Thermo Fisher Scientific, USA). The differential expression analysis was carried out using Partek Genomic Suite 6.6 (Partek).

Supplementary Table S1 Differentially expressed miRNAs in plasma–derived extracellular vesicles from patients with SARS-CoV-2 infection compared with those in healthy control (HC), identified by miRNA NGS analysis*.

Up-regulated miRNAs	Fold change§ (median value)	Down-regulated miRNAs	Fold change§ (median value)
hsa-miR-21	3.01	hsa-miR-215	0.32
hsa-miR-15b	2.96	hsa-miR-342	0.32
hsa-miR-551a	2.95	hsa-miR-335	0.31
hsa-miR-100	2.88	hsa-miR-410	0.30
hsa-miR-158	2.78	hsa-miR-582	0.27
hsa-miR-339	2.78	hsa-miR-708	0.23
hsa-let-7b	2.75	hsa-miR-3159	0.22
hsa-miR-1229	2.58	hsa-miR-371	0.22
hsa-miR-26a	2.57	hsa-miR-873	0.21
hsa-miR-5189	2.46	hsa-miR-214	0.18
hsa-miR-331	2.45	hsa-miR-663a	0.15
hsa-miR-103	2.37	hsa-miR-642	0.15
hsa-miR-503	2.35		
hsa-miR-942	2.32		
hsa-miR-204	2.29		
hsa-miR-597	2.28		
hsa-miR-7	2.28		
hsa-miR-3123	2.25		
hsa-miR-574	2.23		
hsa-miR-1226	2.18		

*miRNA, microRNA; NGS, next generation sequencing.

§Fold change: if the number >2.00 or <0.33, the difference is considered significant.

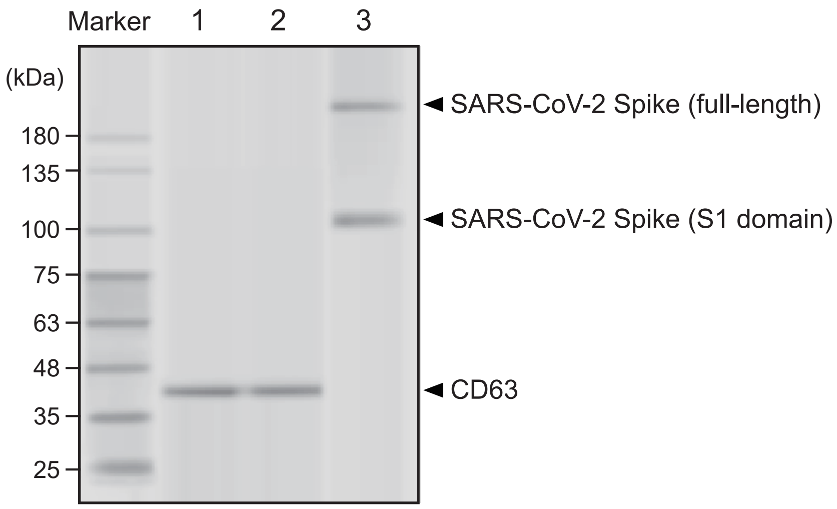

Supplementary Figure S1 There is no spike protein present in spike protein-triggered pEVs. Western blot analysis of naiive platelets-derived extracellular vesicles (pEVs) (lane 1), recombinant SARS-CoV-2 spike protein-triggered pEVs (S-pEVs, lane 2) by using specific anti-CD63 antibody (for EVs detection) and anti-SARS-CoV-2 spike protein antibody simultaneously. Recombinant SARS-CoV-2 spike protein (lane 3) is used as positive control.

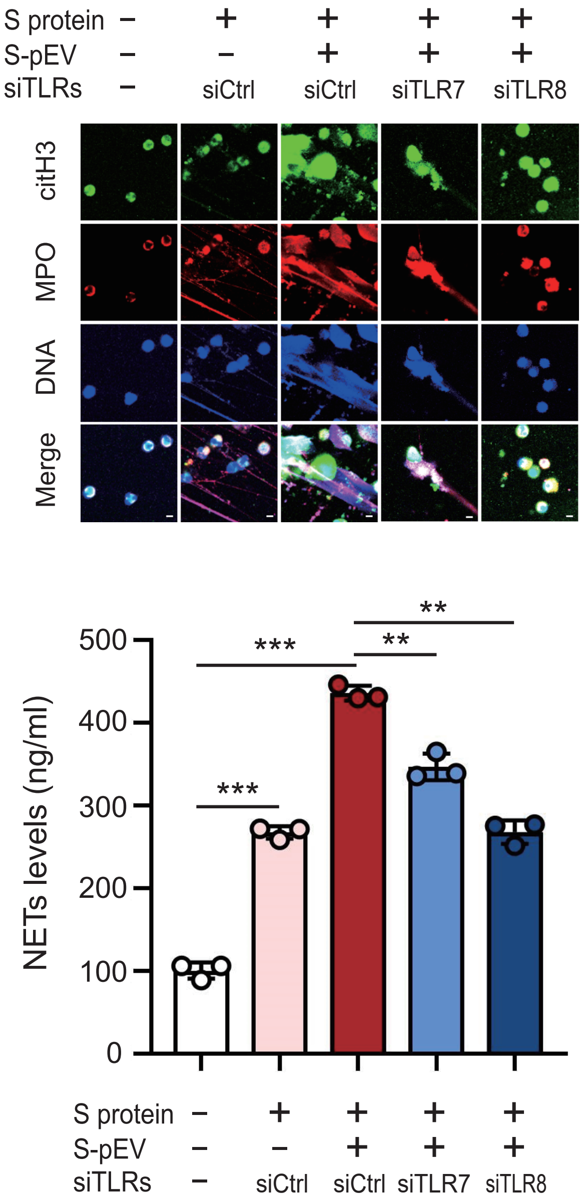

Supplementary Figure S2 Human neutrophils were transfected with control siRNA, TLR7, or TLR8 siRNA (30nM) for 24 h. The SARS-CoV-2 spike protein in the absence or presence of S-pEV was added to control cells, TLR7-, or TLR8-knockdown cells, respectively. After 24h, NETs formation was observed using confocal microscopy (upper panel) and quantified by the MPO-DNA PicoGreen assay (lower panel). The scale bar in the IFA image represents 5 µm. ***P*<0.01, ****P*<0.005.

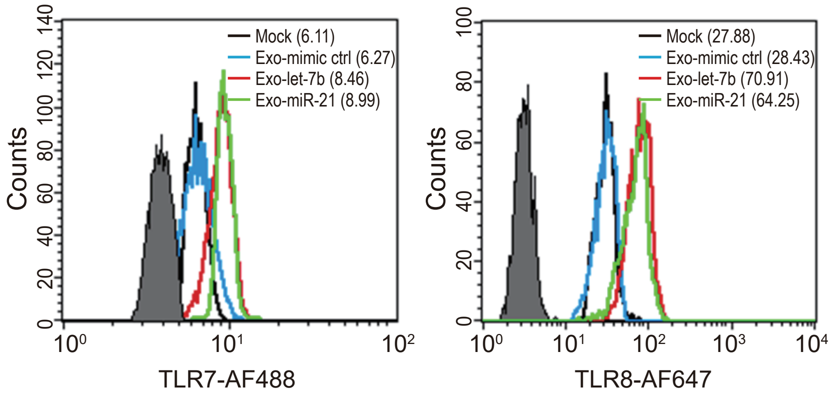

Supplementary Figure S3 pEVs-carried miR-21/let-7b induced TLR7 and TLR8 activation. Human neutrophils were treated with miR-21/let-7b mimic-loaded pEVs. After 4h, the levels of TLR7 (left panel) or TLR8 (right panel) were analyzed using flow cytometry.

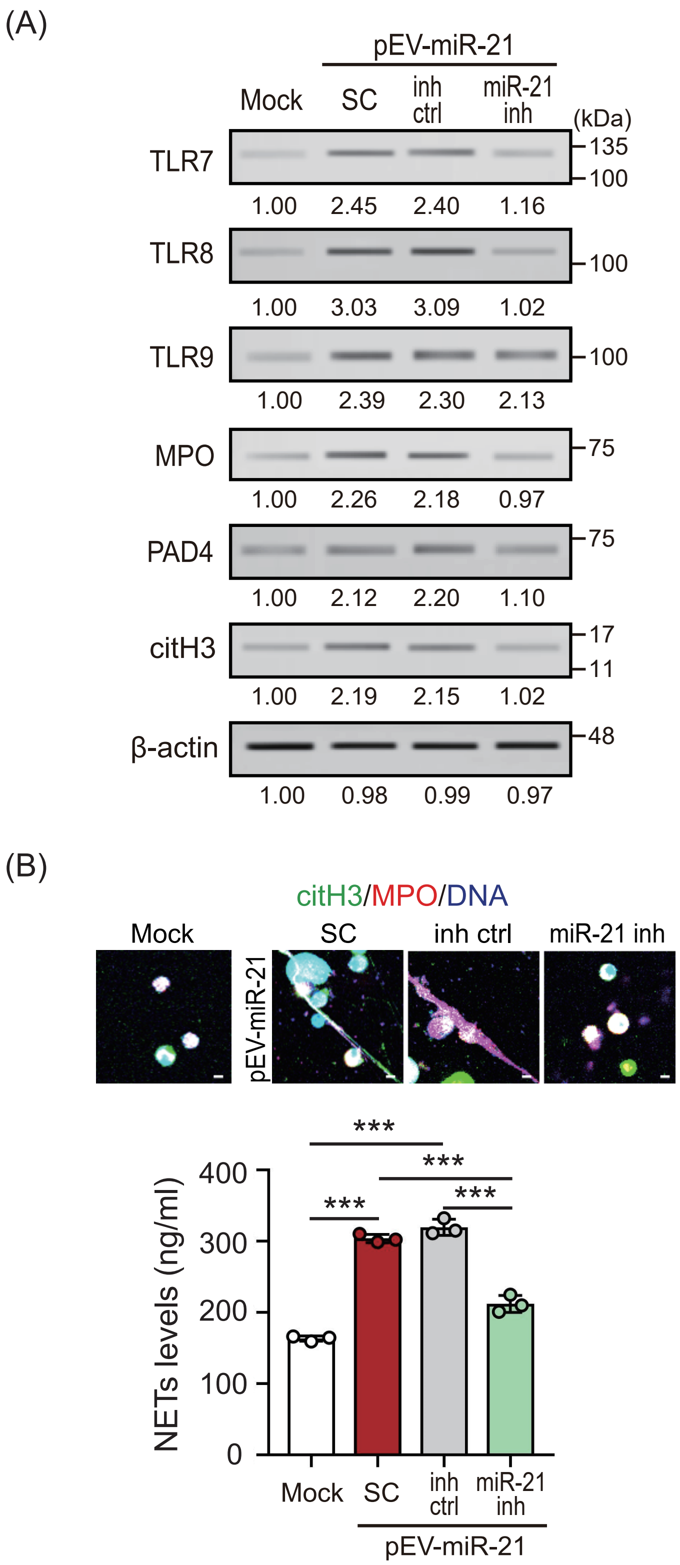

Supplementary Figure S4 Human neutrophils were treated with miR-21 mimic-loaded pEVs in the presence of an miR-21 inhibitor for 24h. (B) The expression of intracellular TLR7/8/9 and NETs-associated proteins was analyzed using immunoblotting. Immunoblotting bands from β-actin were densitometrically measured by ImageJ to determine the lane normalization factor for samples. (B) NETs formation was observed using confocal microscopy (upper panel) and quantified by the MPO-DNA PicoGreen assay (lower panel). The scale bar in the IFA image represents 5μm. The image shown is from a single experiment that is representative of at least three separate experiments. **P*<0.05, ***P*<0.01, ****P*<0.005. SC, solvent control.

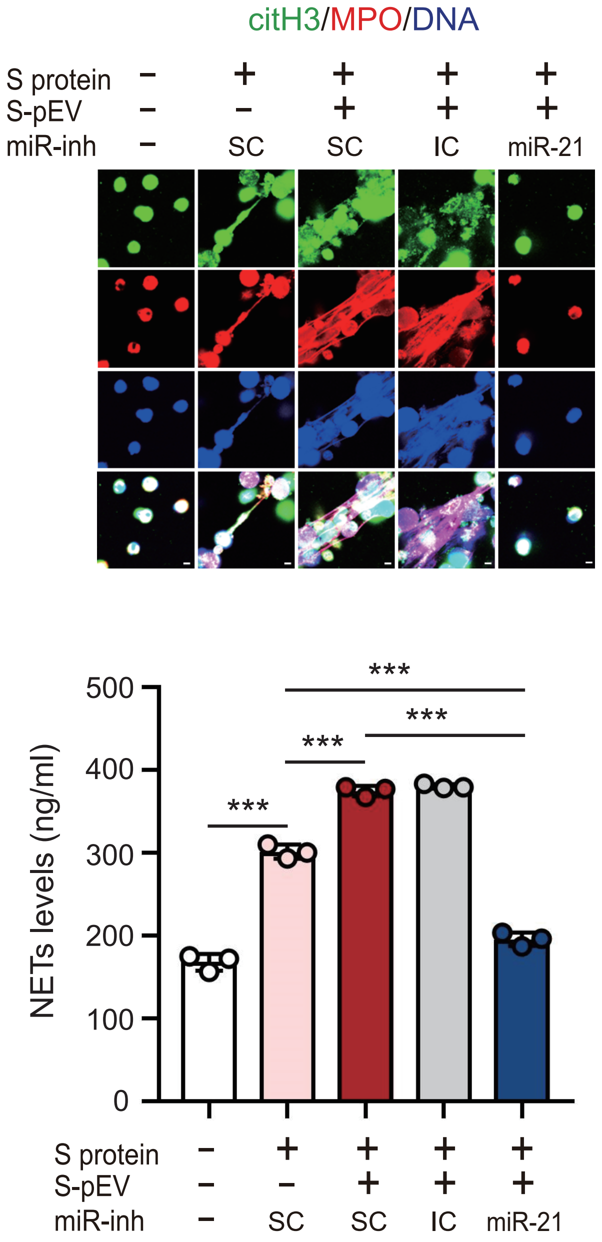

Supplementary Figure S5 Human neutrophils were treated with SARS-CoV-2 spike protein (S protein) or/and spike protein-primed platelets–derived EVs (S-pEV) in the presence of a miRNA inhibitor control or an miR-21 inhibitor for 24h. NETs formation was observed using confocal microscopy (upper panel) and quantified by the MPO-DNA PicoGreen assay (lower panel). The scale bar in the IFA image represents 5μm. The image shown is from a single experiment that is representative of at least three separate experiments. Data are presented as the mean ± SD. **P*<0.05, ***P*<0.01, ****P*<0.005. SC, solvent control. IC, microRNA inhibitor control.

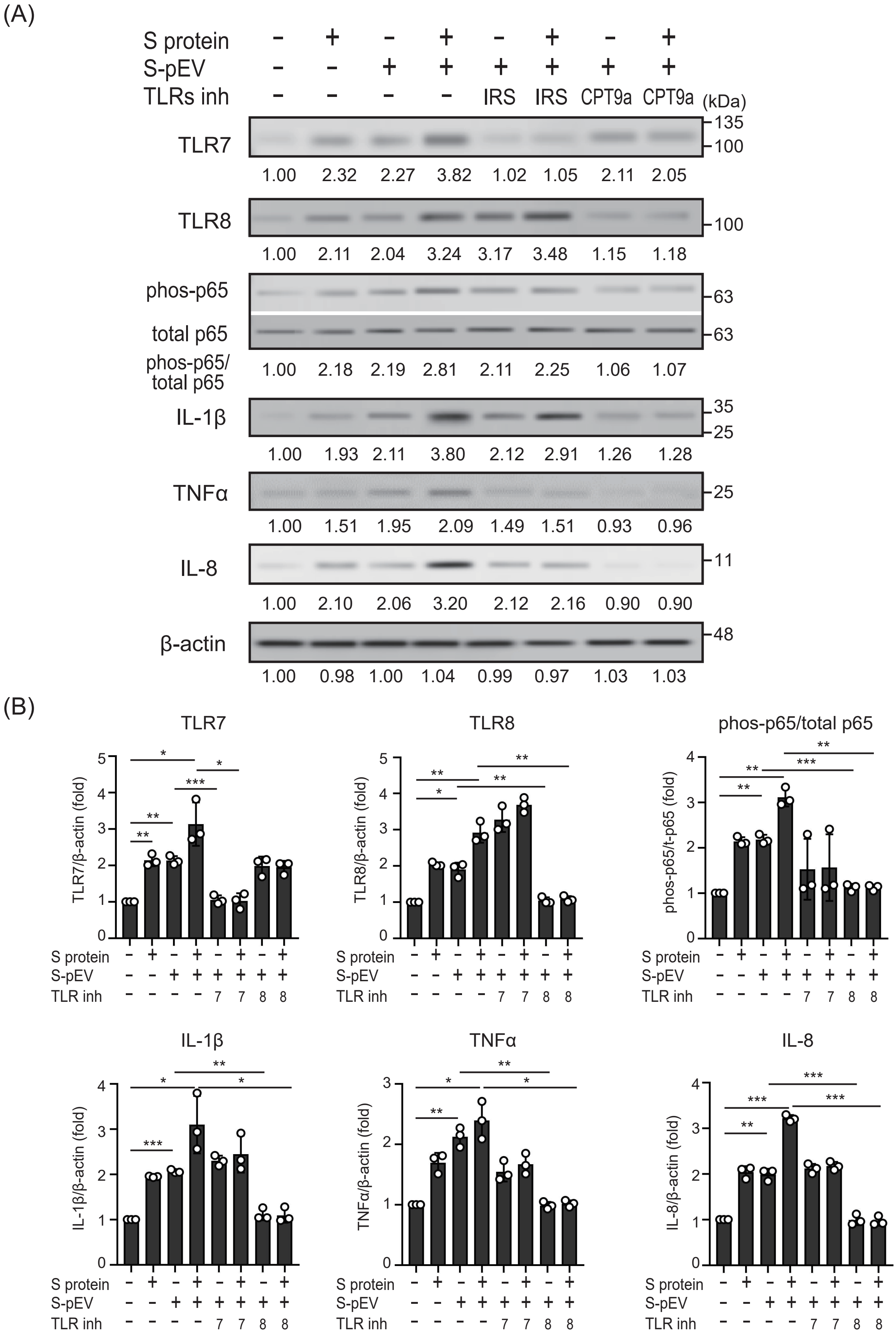

Supplementary Figure S6 Human neutrophils were treated with SARS-CoV-2 spike protein (S protein) or/and spike protein-primed platelets–derived EVs (S-pEV) in the presence of TLR7/8 specific inhibitors for 24h. The intracellular TLR7/8, phosphorylation of p65 subunits and proinflammatory cytokines/chemokines (IL-1β/TNF-α and IL-8) were analyzed using immunoblotting. Immunoblotting bands from β-actin were densitometrically measured by ImageJ to determine the lane normalization factor for samples. All experiments were performed in triplicate and data are presented as the mean±SD. The image shown is from a single experiment that is representative of at least three separate experiments. **P*<0.05, ***P*<0.01, ****P*<0.005.
