# Supplementary material for: SARS-CoV-2 primed platelets–derived microRNAs enhance NETs formation by extracellular vesicle transmission and TLR7/8 activation
Source: Cell Commun Signal. 2023 Oct 30;21:304. doi: 10.1186/s12964-023-01345-4 (PMC10614402; doi:10.1186/s12964-023-01345-4)
Supplement: Supplementary file 3 — Additional file 2. [file 12964_2023_1345_MOESM2_ESM.pdf]

Additional file 2. Densitometric analysis of immunoblot results presented in this study. Immunoblots were quantitated by densitometric analysis using ImageJ software and normalized to β-actin. Numbers below each lane are relative fold of the control level of a specific protein in mock-treated cells. All results were obtained in three independent experiments, and the data is presented as the mean±SD. *$P<0.05$, ** $P<0.01$, *** $P<0.005$.

Fig. 1E

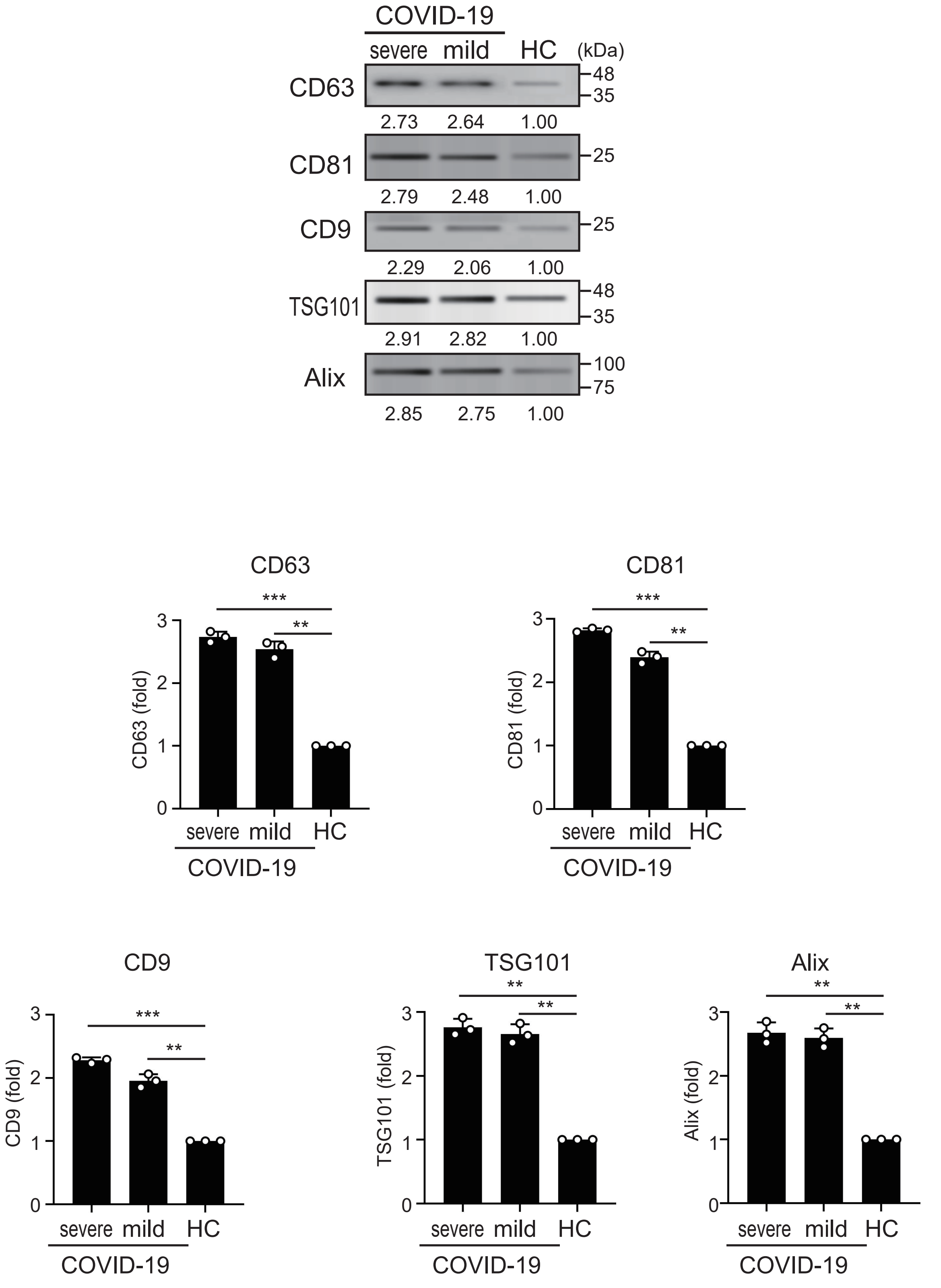
COVID-19
severe mild HC (kDa)
CD63
48
35
2.73 2.64 1.00
CD81
25
2.79 2.48 1.00
CD9
25
2.29 2.06 1.00
TSG101
48
35
2.91 2.82 1.00
Alix
100
75
2.85 2.75 1.00
CD63
CD63 (fold)
***
**
CD81
CD81 (fold)
***
**
CD9
CD9 (fold)
***
**
TSG101
TSG101 (fold)
**
**
Alix
Alix (fold)
**
**
0
1
2
3
severe mild HC
COVID-19

Fig. 2E

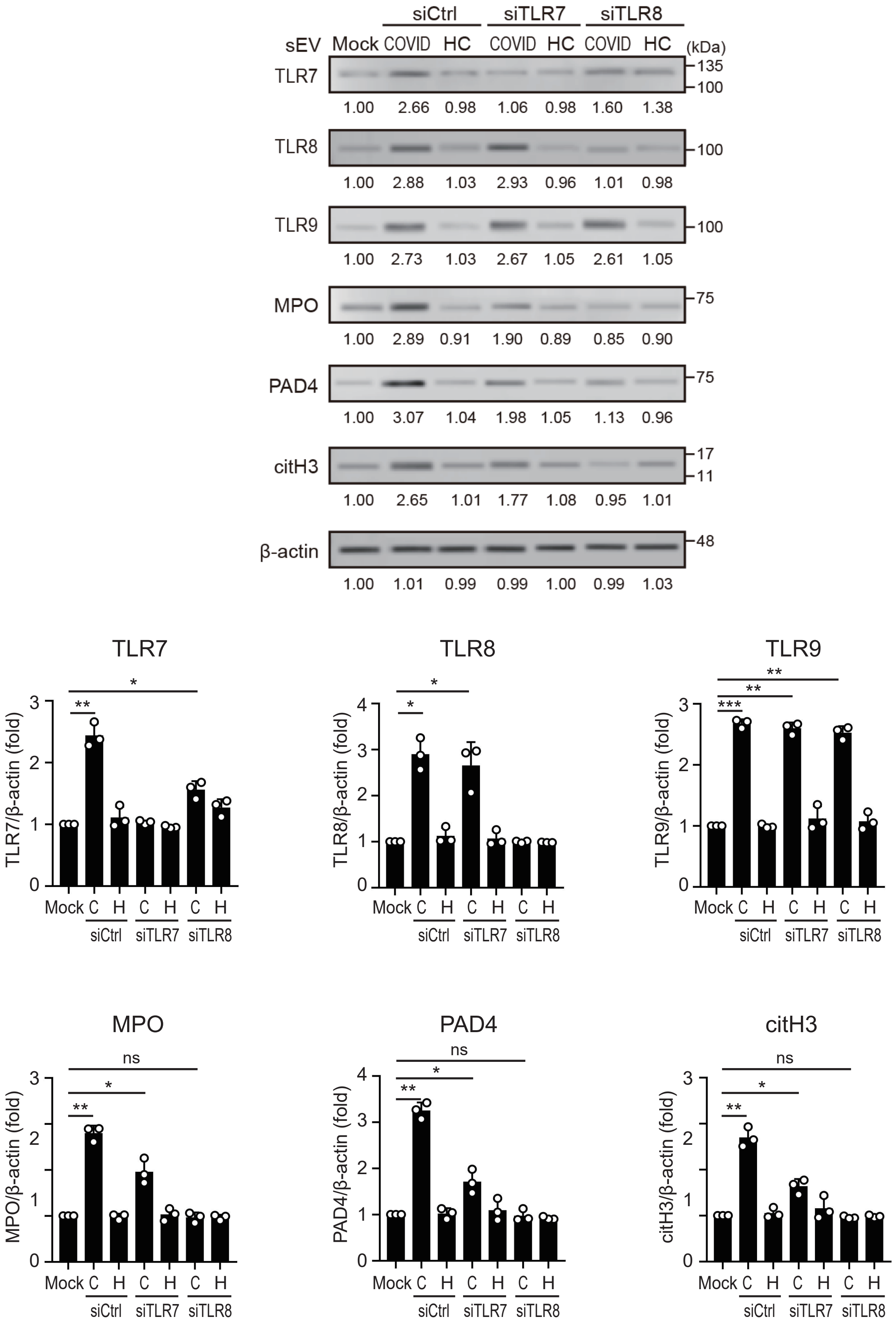

siCtrl
siTLR7
siTLR8
sEV
Mock
COVID
HC
(kDa)
TLR7
135
100
1.00 2.66 0.98 1.06 0.98 1.60 1.38
TLR8
100
1.00 2.88 1.03 2.93 0.96 1.01 0.98
TLR9
100
1.00 2.73 1.03 2.67 1.05 2.61 1.05
MPO
75
1.00 2.89 0.91 1.90 0.89 0.85 0.90
PAD4
75
1.00 3.07 1.04 1.98 1.05 1.13 0.96
citH3
17
11
1.00 2.65 1.01 1.77 1.08 0.95 1.01
β-actin
48
1.00 1.01 0.99 0.99 1.00 0.99 1.03
TLR7
TLR7/β-actin (fold)
TLR8
TLR8/β-actin (fold)
TLR9
TLR9/β-actin (fold)
MPO
MPO/β-actin (fold)
PAD4
PAD4/β-actin (fold)
citH3
citH3/β-actin (fold)
ns
Mock C H C H C H
siCtrl siTLR7 siTLR8

Fig. 3C

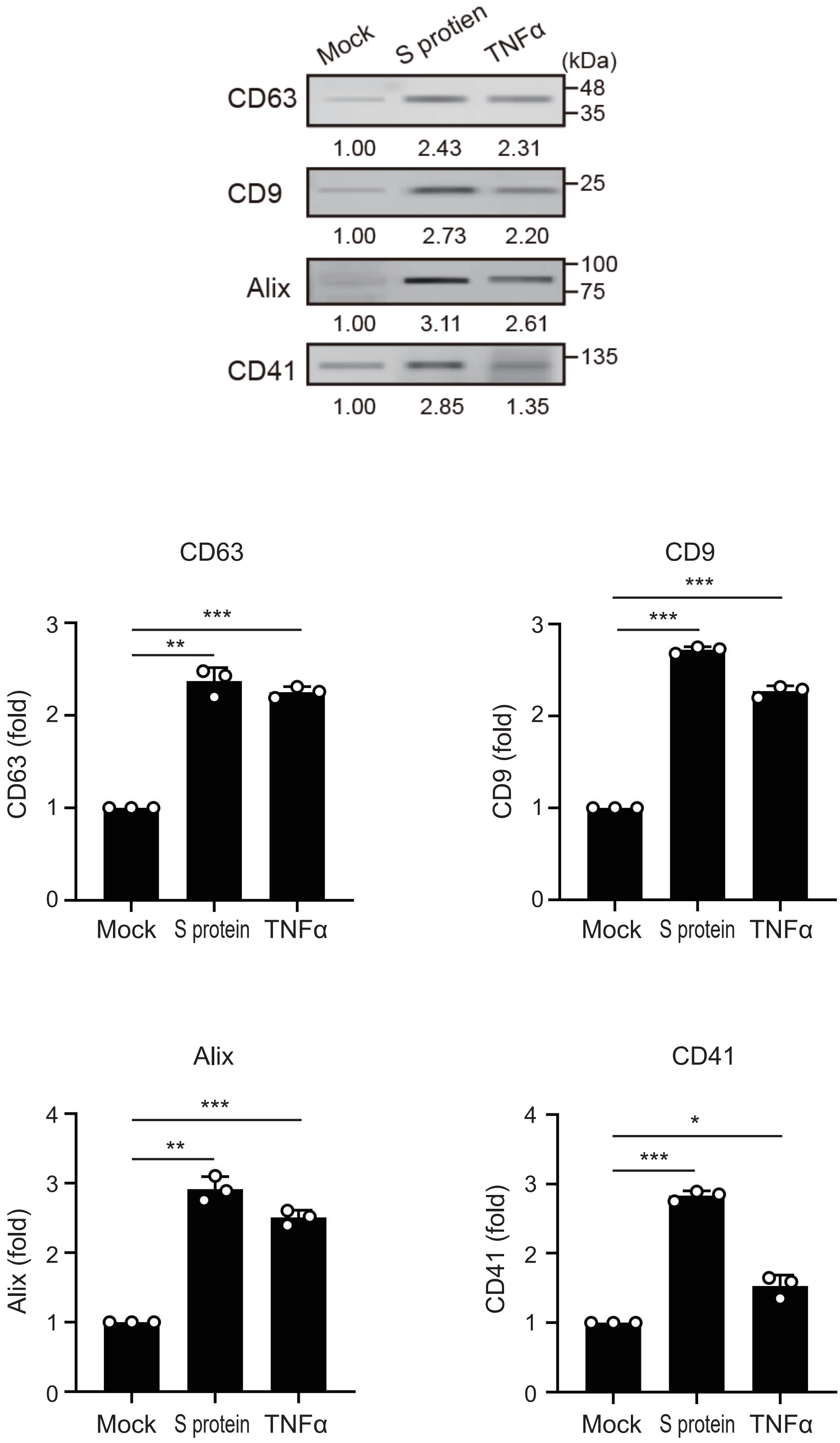
Mock
S protien
TNFα
(kDa)
CD63
48
35
1.00 2.43 2.31
CD9
25
1.00 2.73 2.20
Alix
100
75
1.00 3.11 2.61
CD41
135
1.00 2.85 1.35
CD63
CD63 (fold)
***
**
Mock S protein TNFα
CD9
CD9 (fold)
***
***
Mock S protein TNFα
Alix
Alix (fold)
***
**
Mock S protein TNFα
CD41
CD41 (fold)
*
***
Mock S protein TNFα

Fig. 3G

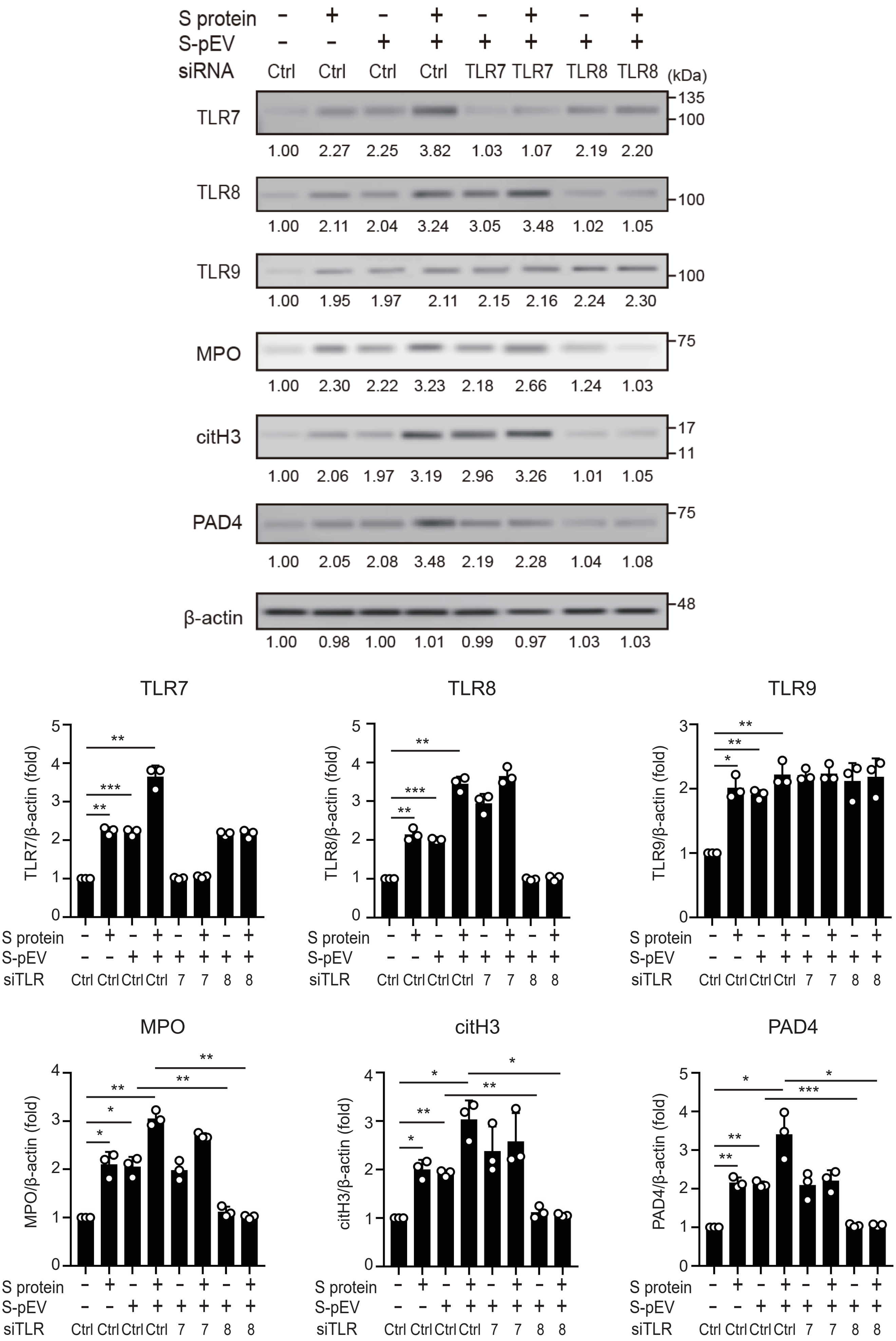
S protein
S-pEV
siRNA
Ctrl Ctrl Ctrl Ctrl TLR7 TLR7 TLR8 TLR8
(kDa)
TLR7
1.00 2.27 2.25 3.82 1.03 1.07 2.19 2.20
135
100
TLR8
1.00 2.11 2.04 3.24 3.05 3.48 1.02 1.05
TLR9
1.00 1.95 1.97 2.11 2.15 2.16 2.24 2.30
MPO
1.00 2.30 2.22 3.23 2.18 2.66 1.24 1.03
75
citH3
1.00 2.06 1.97 3.19 2.96 3.26 1.01 1.05
17
11
PAD4
1.00 2.05 2.08 3.48 2.19 2.28 1.04 1.08
β-actin
1.00 0.98 1.00 1.01 0.99 0.97 1.03 1.03
48
TLR7
TLR7/β-actin (fold)
TLR8
TLR8/β-actin (fold)
TLR9
TLR9/β-actin (fold)
MPO
MPO/β-actin (fold)
citH3
citH3/β-actin (fold)
PAD4
PAD4/β-actin (fold)
siTLR
Ctrl Ctrl Ctrl Ctrl 7 7 8 8

Fig. 5E

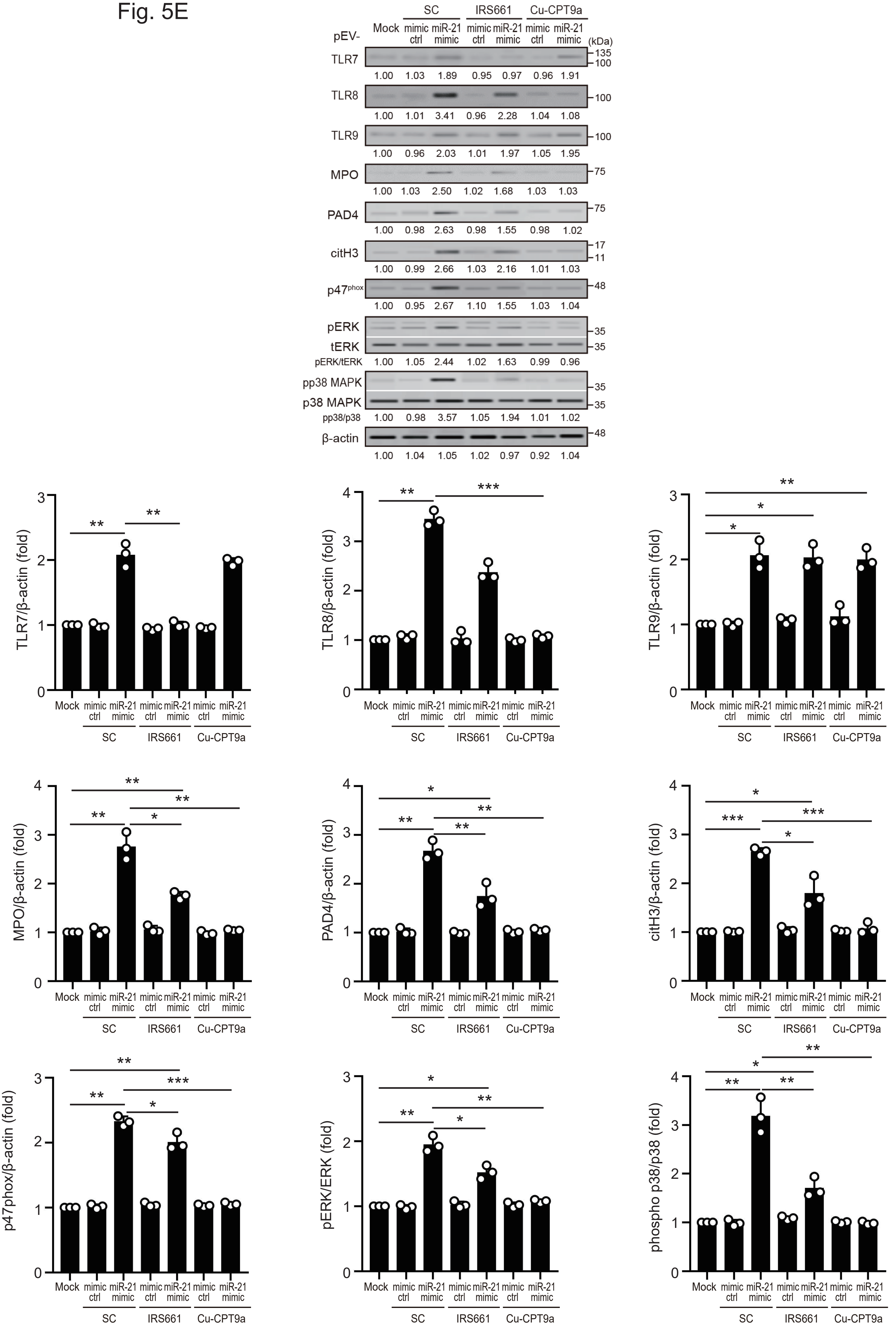

SC
IRS661
Cu-CPT9a
pEV-
Mock
mimic ctrl
miR-21 mimic
(kDa)
TLR7
135
100
1.00 1.03 1.89 0.95 0.97 0.96 1.91
TLR8
100
1.00 1.01 3.41 0.96 2.28 1.04 1.08
TLR9
100
1.00 0.96 2.03 1.01 1.97 1.05 1.95
MPO
75
1.00 1.03 2.50 1.02 1.68 1.03 1.03
PAD4
75
1.00 0.98 2.63 0.98 1.55 0.98 1.02
citH3
17
11
1.00 0.99 2.66 1.03 2.16 1.01 1.03
p47phox
48
1.00 0.95 2.67 1.10 1.55 1.03 1.04
pERK
tERK
35
35
pERK/tERK 1.00 1.05 2.44 1.02 1.63 0.99 0.96
pp38 MAPK
p38 MAPK
35
35
pp38/p38 1.00 0.98 3.57 1.05 1.94 1.01 1.02
β-actin
48
1.00 1.04 1.05 1.02 0.97 0.92 1.04
TLR7/β-actin (fold)
TLR8/β-actin (fold)
TLR9/β-actin (fold)
MPO/β-actin (fold)
PAD4/β-actin (fold)
citH3/β-actin (fold)
p47phox/β-actin (fold)
pERK/ERK (fold)
phospho p38/p38 (fold)

Fig. 5G

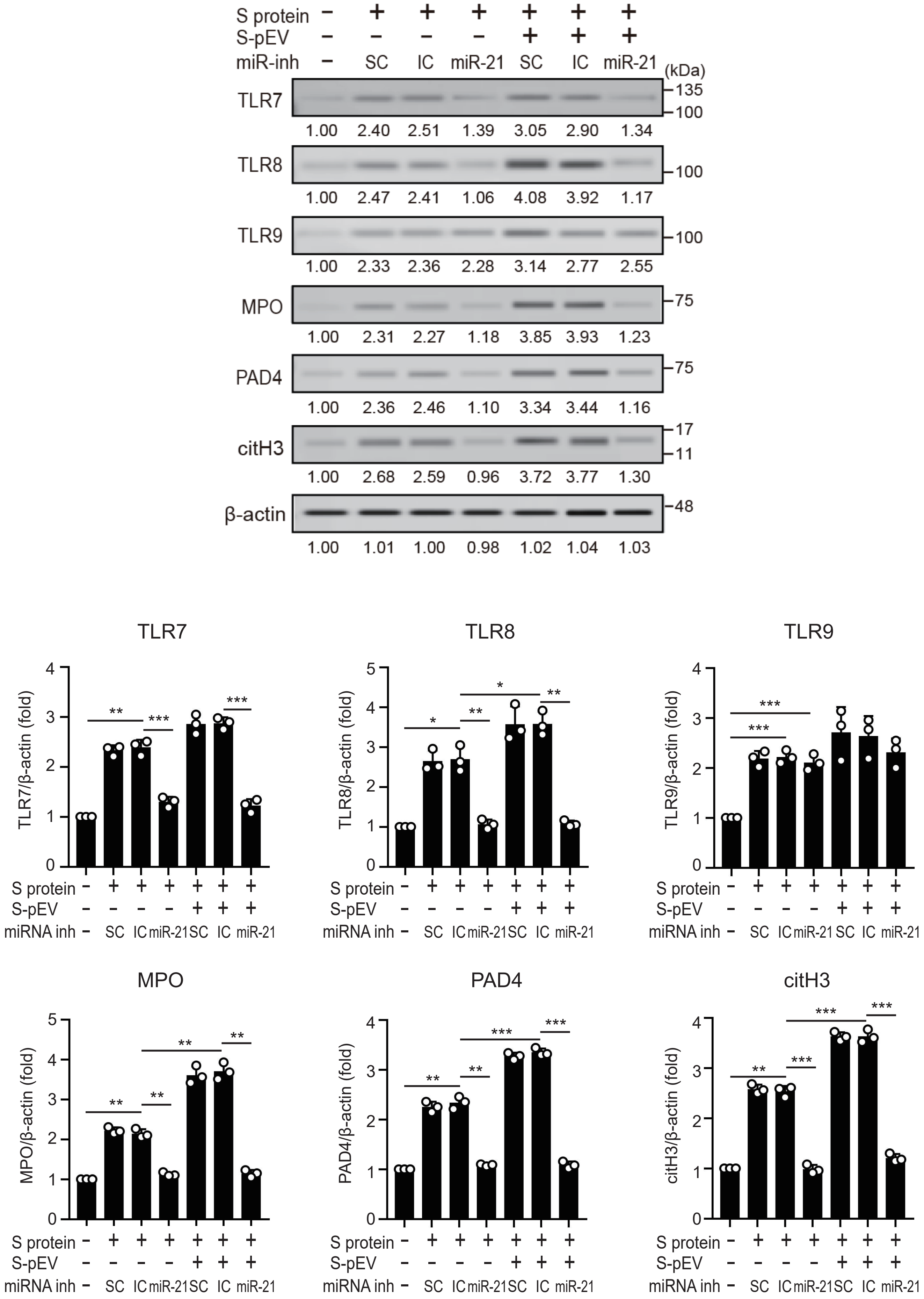
S protein − + + + + + +
S-pEV − − − − + + +
miR-inh − SC IC miR-21 SC IC miR-21
(kDa)
TLR7
135
100
1.00 2.40 2.51 1.39 3.05 2.90 1.34
TLR8
100
1.00 2.47 2.41 1.06 4.08 3.92 1.17
TLR9
100
1.00 2.33 2.36 2.28 3.14 2.77 2.55
MPO
75
1.00 2.31 2.27 1.18 3.85 3.93 1.23
PAD4
75
1.00 2.36 2.46 1.10 3.34 3.44 1.16
citH3
17
11
1.00 2.68 2.59 0.96 3.72 3.77 1.30
β-actin
48
1.00 1.01 1.00 0.98 1.02 1.04 1.03
TLR7
TLR7/β-actin (fold)
TLR8
TLR8/β-actin (fold)
TLR9
TLR9/β-actin (fold)
MPO
MPO/β-actin (fold)
PAD4
PAD4/β-actin (fold)
citH3
citH3/β-actin (fold)
S protein - + + + + + +
S-pEV - - - - + + +
miRNA inh - SC IC miR-21 SC IC miR-21

Fig. 6E

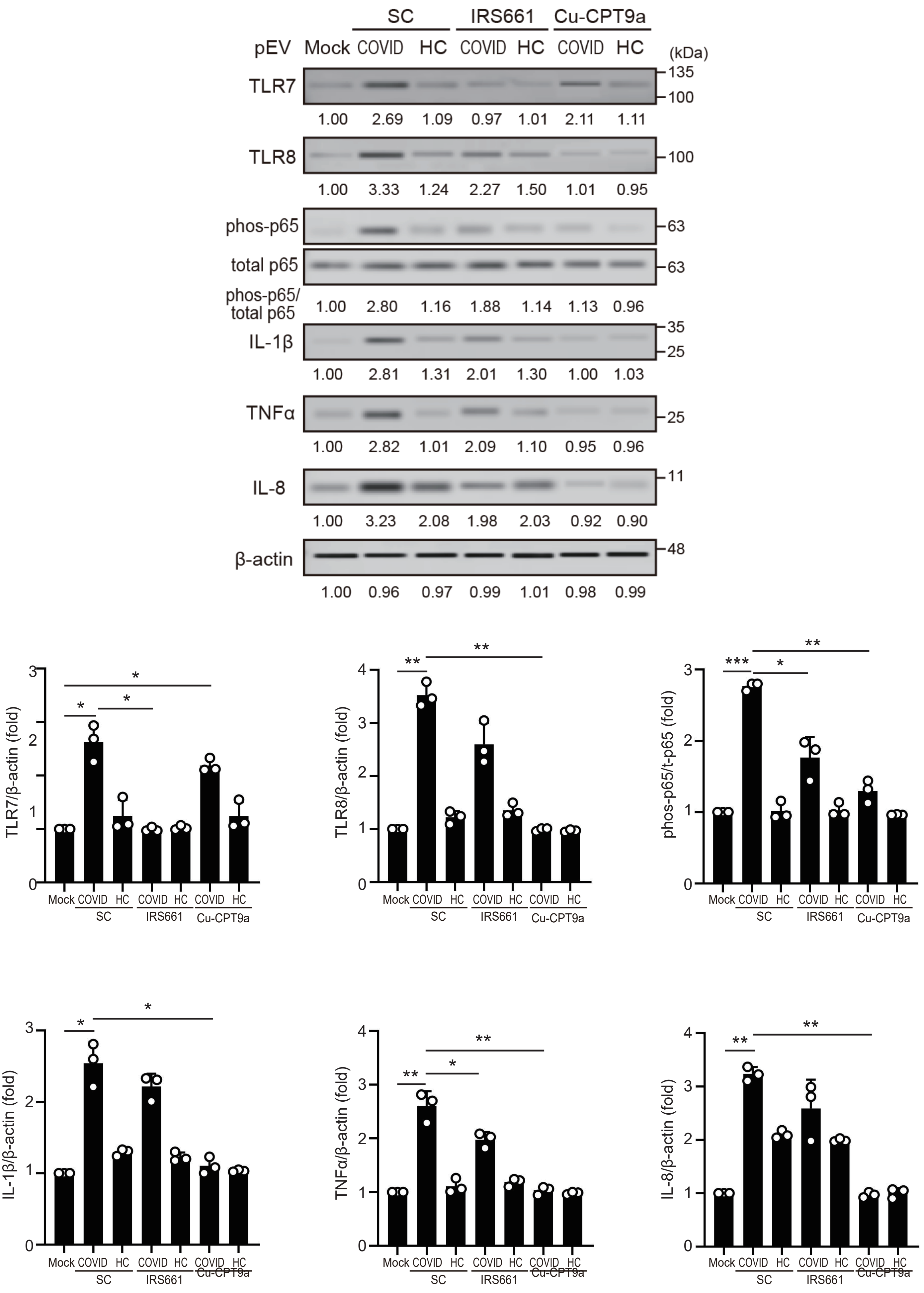

SC
IRS661
Cu-CPT9a
pEV Mock COVID HC COVID HC COVID HC
(kDa)
TLR7
135
100
1.00 2.69 1.09 0.97 1.01 2.11 1.11
TLR8
100
1.00 3.33 1.24 2.27 1.50 1.01 0.95
phos-p65
63
total p65
63
phos-p65/ total p65
1.00 2.80 1.16 1.88 1.14 1.13 0.96
IL-1β
35
25
1.00 2.81 1.31 2.01 1.30 1.00 1.03
TNFα
25
1.00 2.82 1.01 2.09 1.10 0.95 0.96
IL-8
11
1.00 3.23 2.08 1.98 2.03 0.92 0.90
β-actin
48
1.00 0.96 0.97 0.99 1.01 0.98 0.99
TLR7/β-actin (fold)
TLR8/β-actin (fold)
phos-p65/t-p65 (fold)
IL-1β/β-actin (fold)
TNFα/β-actin (fold)
IL-8/β-actin (fold)
Mock COVID HC COVID HC COVID HC
SC IRS661 Cu-CPT9a

Fig. 6F

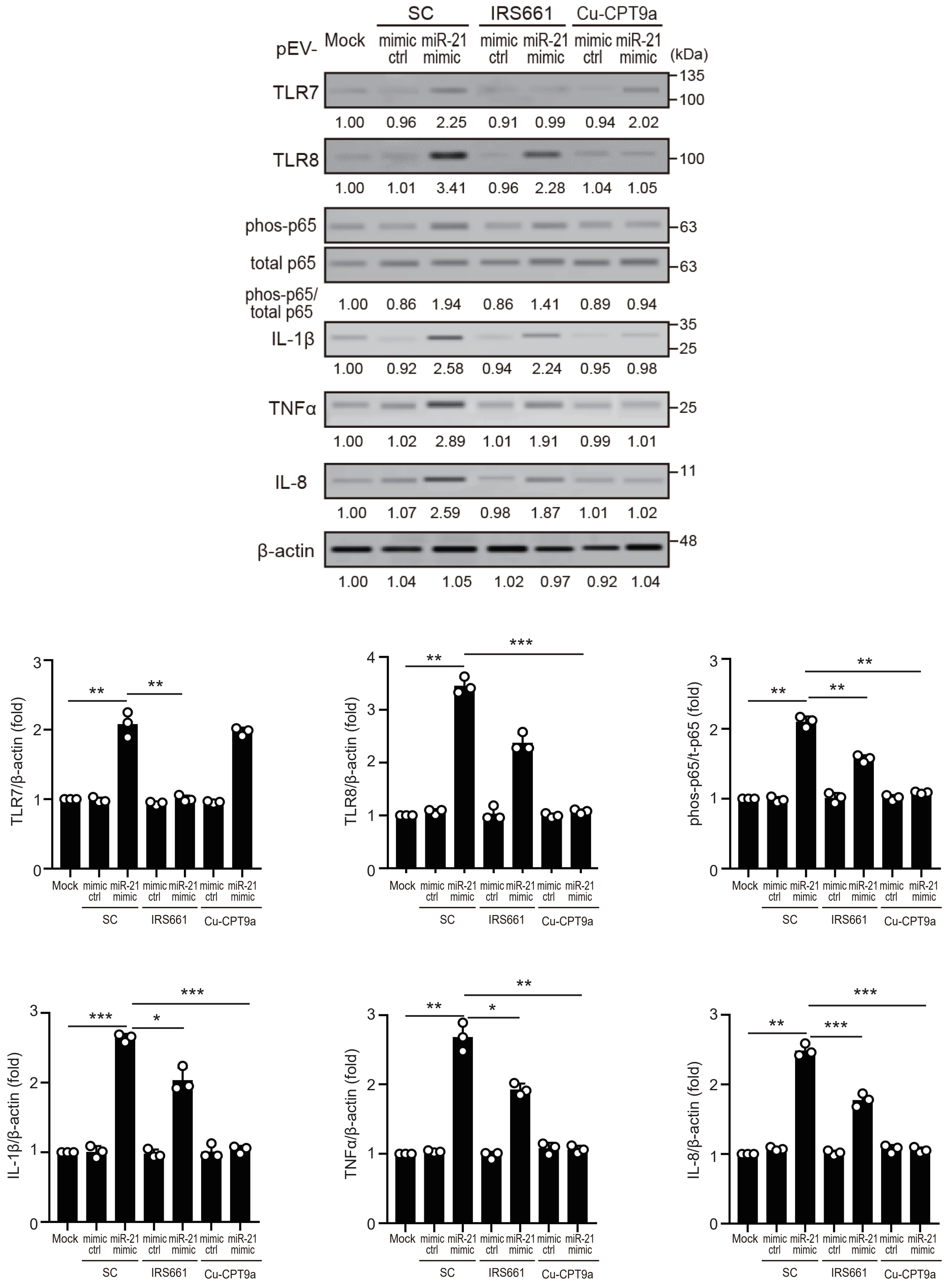

SC
IRS661
Cu-CPT9a
pEV-
Mock
mimic ctrl
miR-21 mimic
(kDa)
TLR7
135
100
1.00 0.96 2.25 0.91 0.99 0.94 2.02
TLR8
100
1.00 1.01 3.41 0.96 2.28 1.04 1.05
phos-p65
63
total p65
63
phos-p65/ total p65
1.00 0.86 1.94 0.86 1.41 0.89 0.94
IL-1β
35
25
1.00 0.92 2.58 0.94 2.24 0.95 0.98
TNFα
25
1.00 1.02 2.89 1.01 1.91 0.99 1.01
IL-8
11
1.00 1.07 2.59 0.98 1.87 1.01 1.02
β-actin
48
1.00 1.04 1.05 1.02 0.97 0.92 1.04
TLR7/β-actin (fold)
TLR8/β-actin (fold)
phos-p65/t-p65 (fold)
IL-1β/β-actin (fold)
TNFα/β-actin (fold)
IL-8/β-actin (fold)

Fig. S3

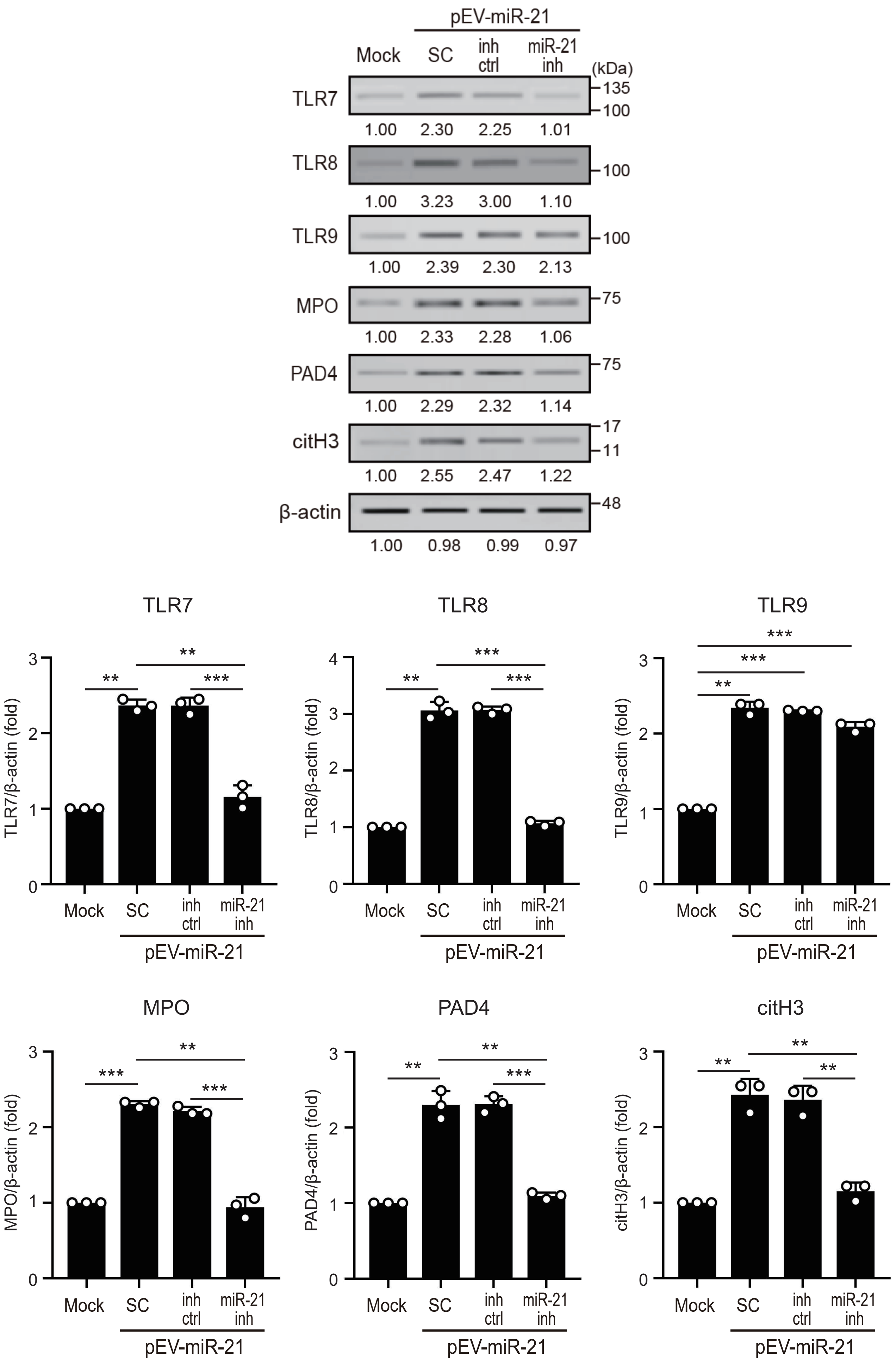
pEV-miR-21
Mock
SC
inh ctrl
miR-21 inh
(kDa)
TLR7
135
100
1.00 2.30 2.25 1.01
TLR8
100
1.00 3.23 3.00 1.10
TLR9
100
1.00 2.39 2.30 2.13
MPO
75
1.00 2.33 2.28 1.06
PAD4
75
1.00 2.29 2.32 1.14
citH3
17
11
1.00 2.55 2.47 1.22
β-actin
48
1.00 0.98 0.99 0.97
TLR7
TLR7/β-actin (fold)
TLR8
TLR8/β-actin (fold)
TLR9
TLR9/β-actin (fold)
MPO
MPO/β-actin (fold)
PAD4
PAD4/β-actin (fold)
citH3
citH3/β-actin (fold)
**
***
pEV-miR-21
