# Supplementary material for: SARS-CoV-2 primed platelets–derived microRNAs enhance NETs formation by extracellular vesicle transmission and TLR7/8 activation
Source: Cell Commun Signal. 2023 Oct 30;21:304. doi: 10.1186/s12964-023-01345-4 (PMC10614402; doi:10.1186/s12964-023-01345-4)
Supplement: Supplementary file 4 — Additional file 3. [file 12964_2023_1345_MOESM3_ESM.pdf]

Fig. 1E

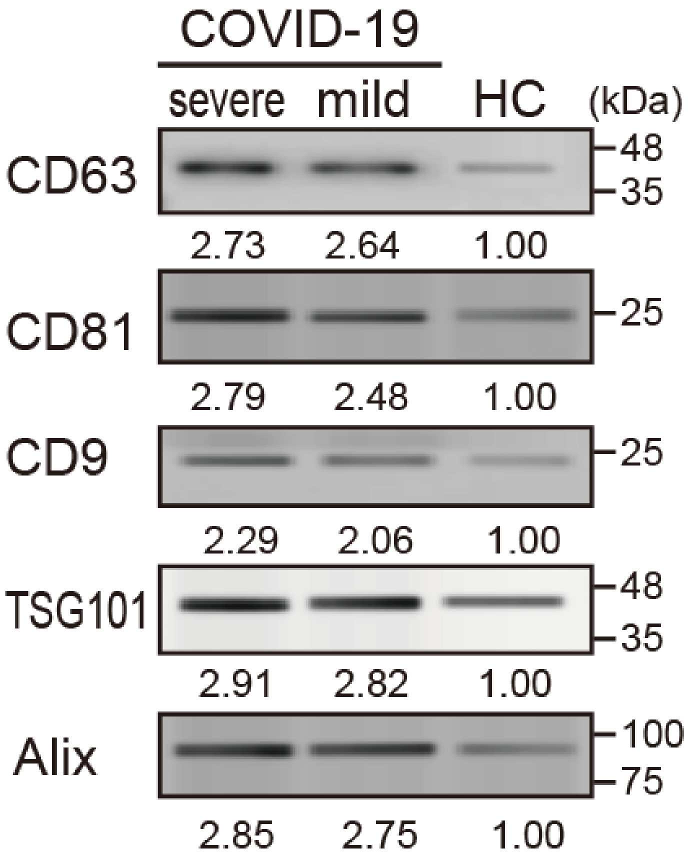
COVID-19
severe
mild
HC
(kDa)
CD63
48
35
2.73 2.64 1.00
CD81
25
2.79 2.48 1.00
CD9
25
2.29 2.06 1.00
TSG101
48
35
2.91 2.82 1.00
Alix
100
75
2.85 2.75 1.00

CD63 (43 kDa)

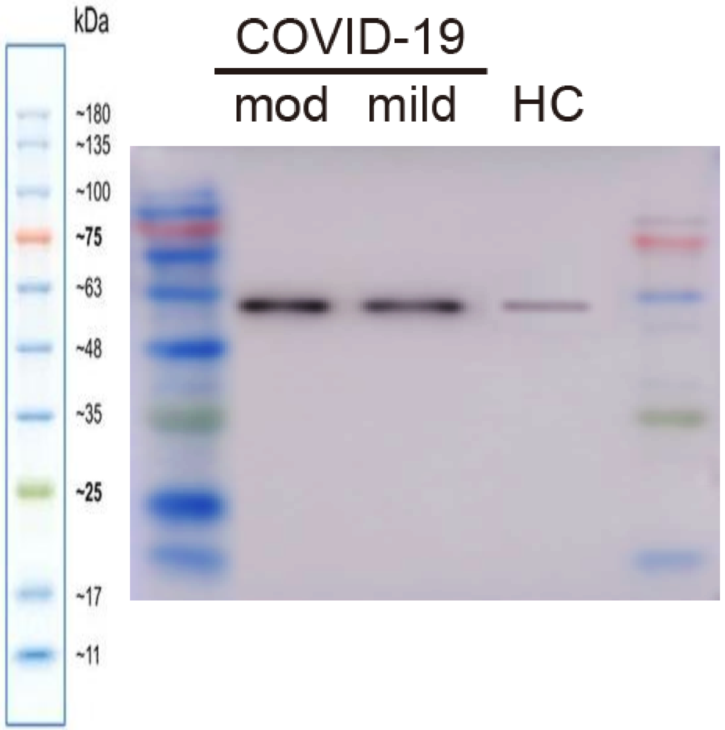

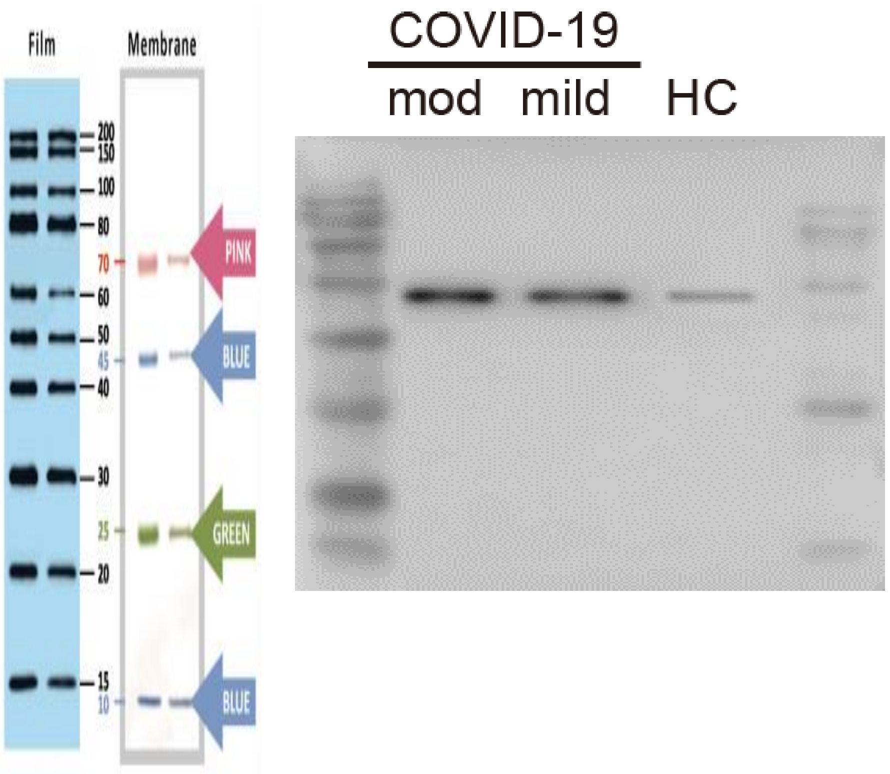

CD63 (43 kDa)

CD81 (25 kDa)

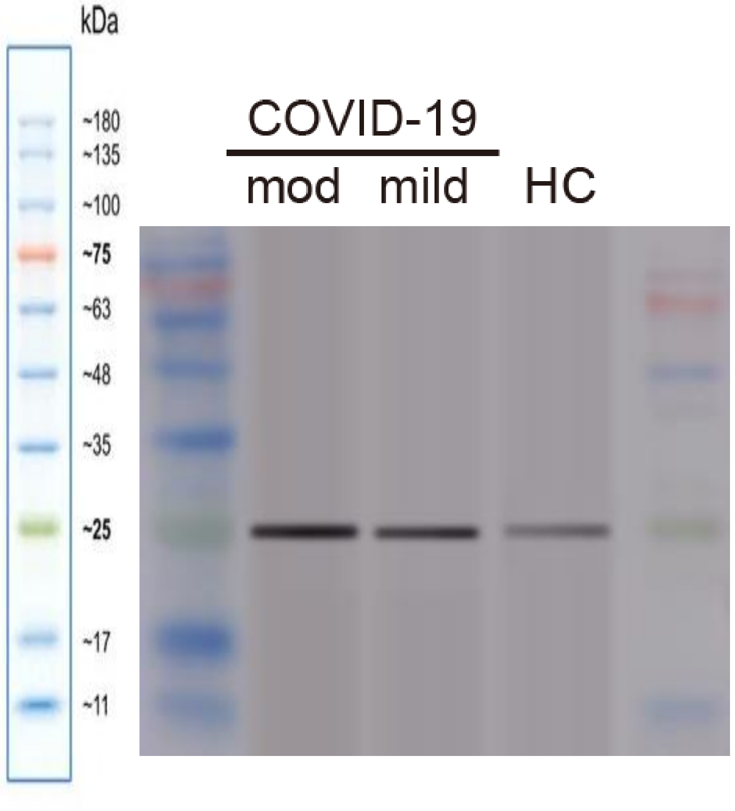

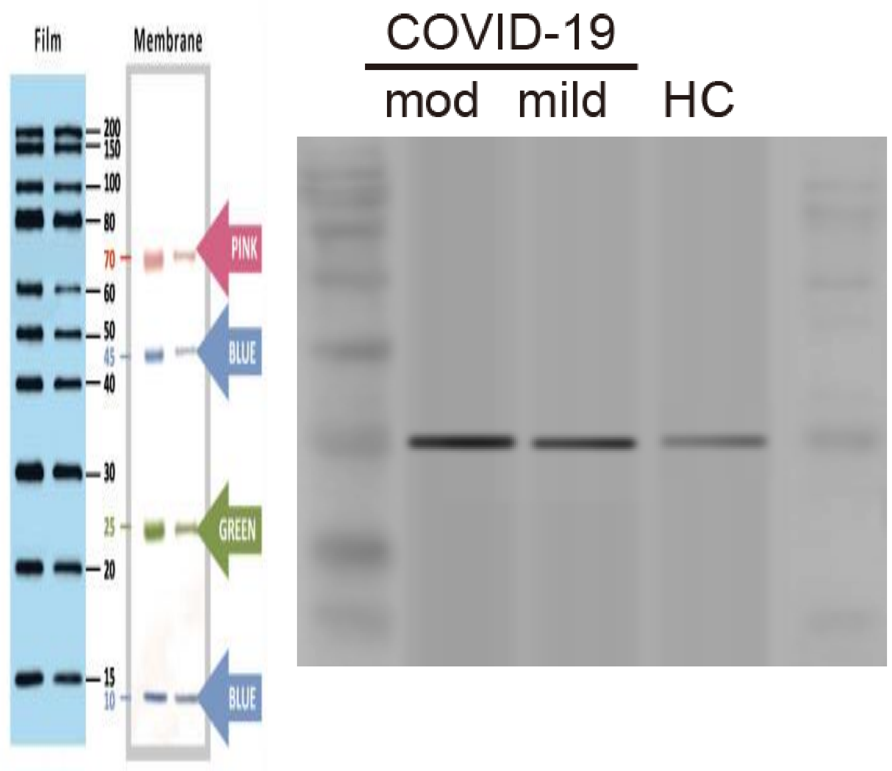

CD81 (25 kDa)

CD9 (24 kDa)

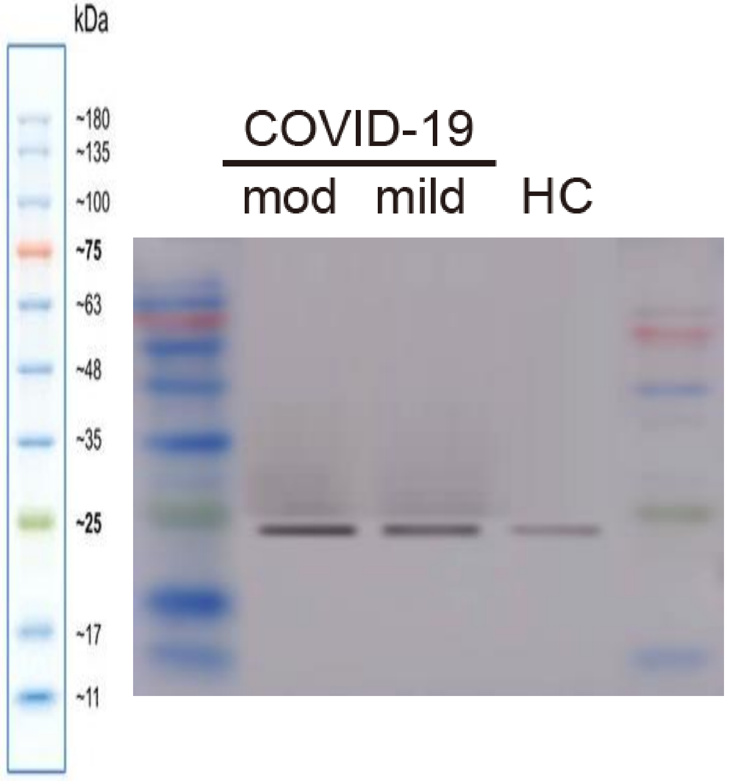

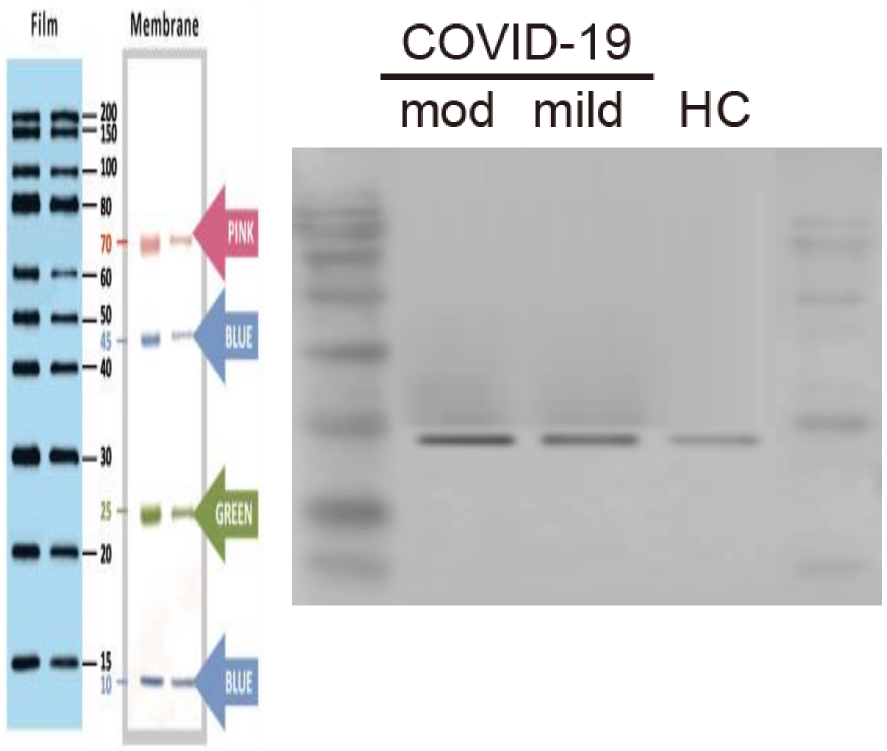

CD9 (24 kDa)

Tsg101 (45 kDa)

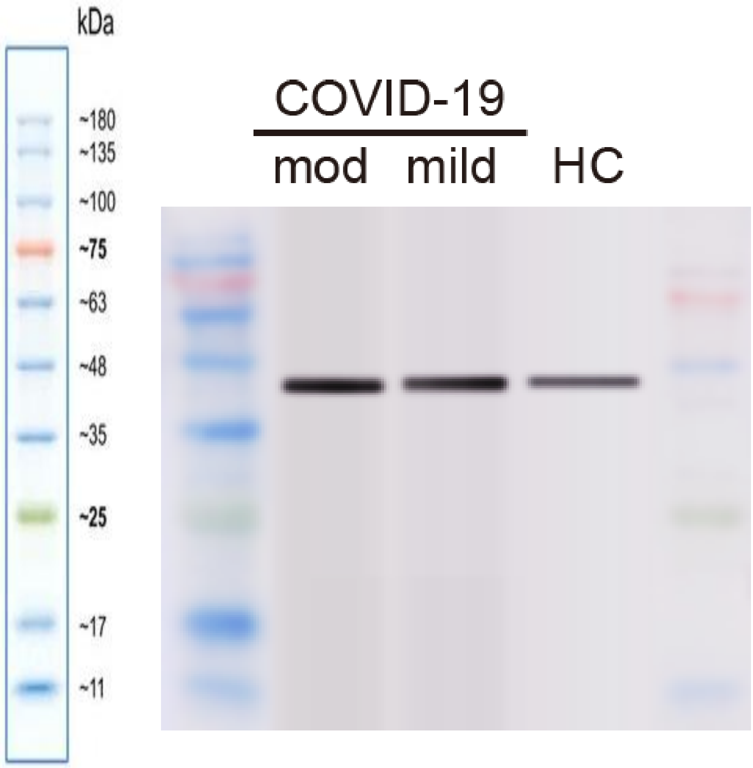

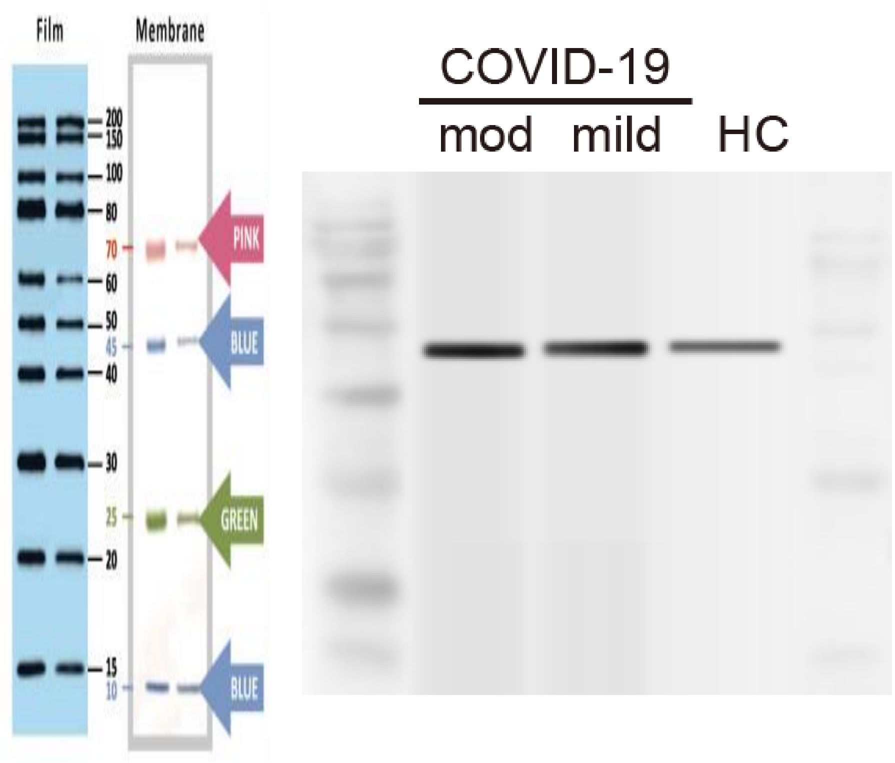

Tsg101 (45 kDa)

Alix (95 kDa)

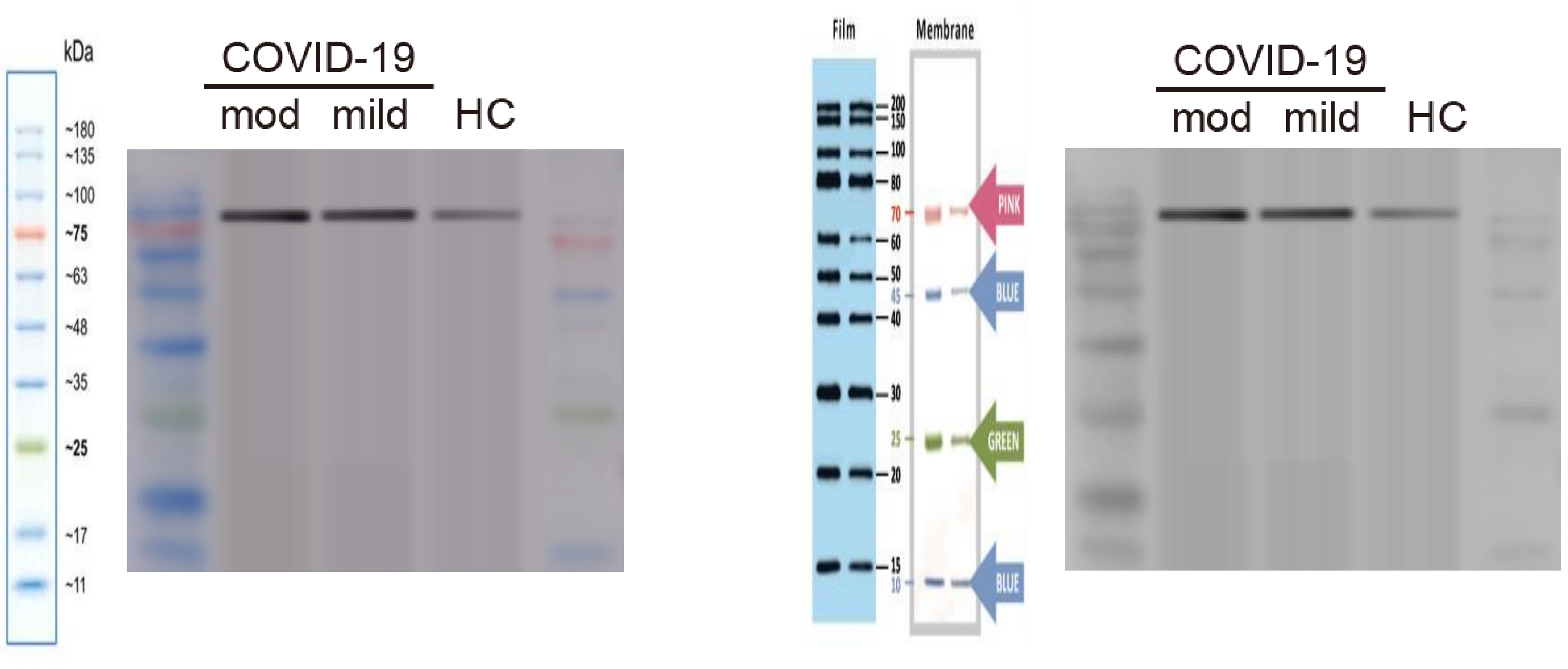

Alix (95 kDa)

Fig. 2E

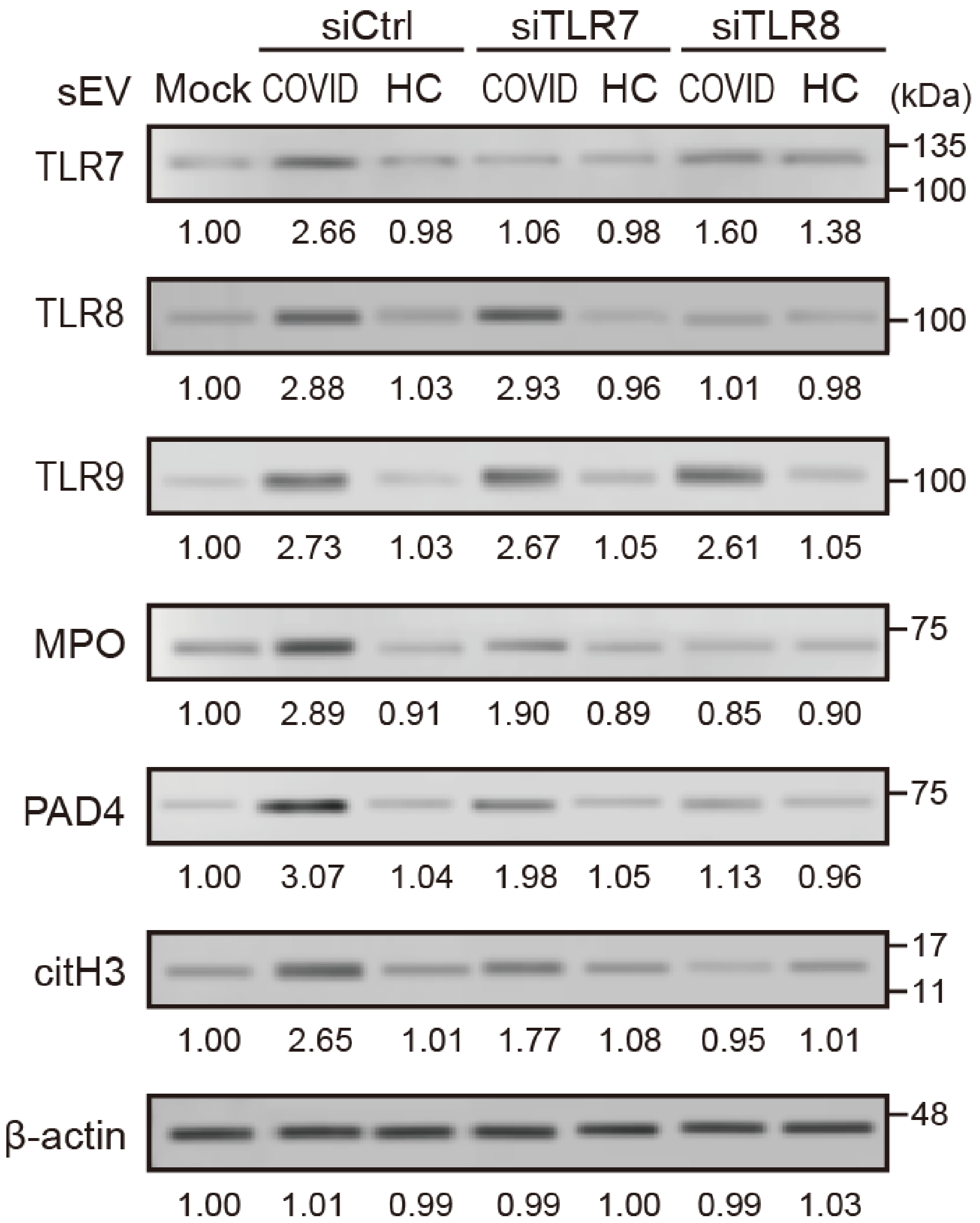
siCtrl
siTLR7
siTLR8
sEV
Mock
COVID
HC
COVID
HC
COVID
HC
(kDa)
TLR7
135
100
1.00 2.66 0.98 1.06 0.98 1.60 1.38
TLR8
100
1.00 2.88 1.03 2.93 0.96 1.01 0.98
TLR9
100
1.00 2.73 1.03 2.67 1.05 2.61 1.05
MPO
75
1.00 2.89 0.91 1.90 0.89 0.85 0.90
PAD4
75
1.00 3.07 1.04 1.98 1.05 1.13 0.96
citH3
17
11
1.00 2.65 1.01 1.77 1.08 0.95 1.01
β-actin
48
1.00 1.01 0.99 0.99 1.00 0.99 1.03

TLR7 (121 kDa)

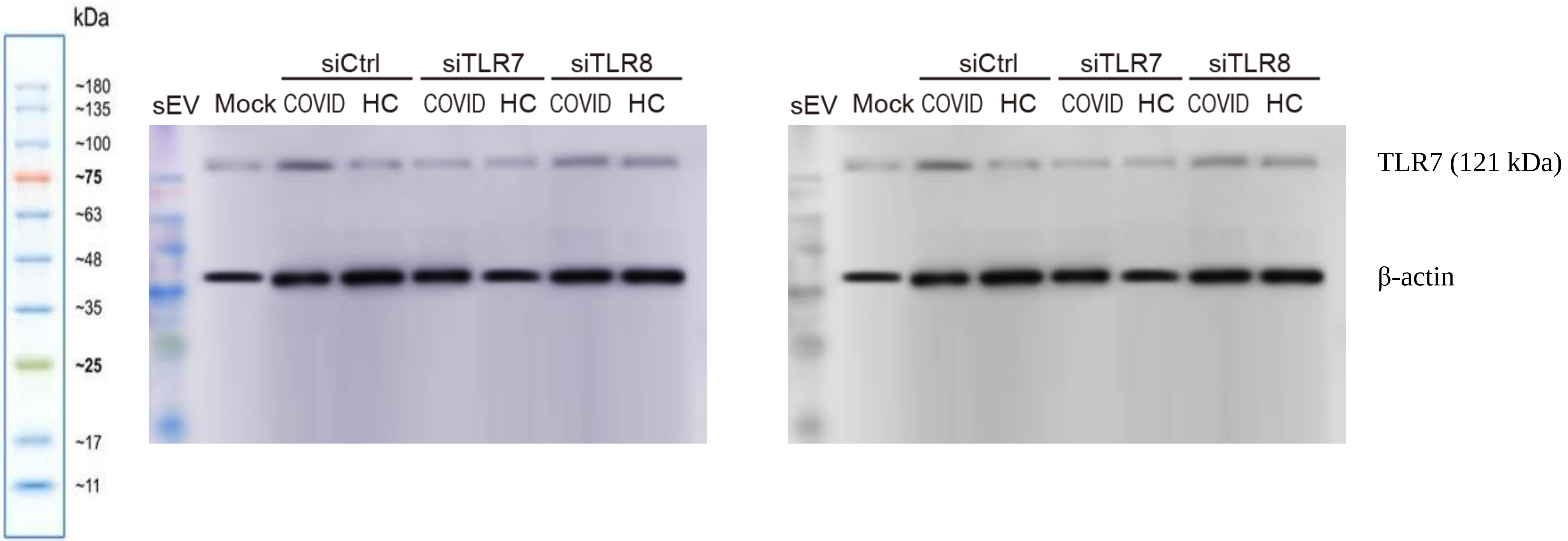
kDa
~180
~135
~100
~75
~63
~48
~35
~25
~17
~11
siCtrl
siTLR7
siTLR8
sEV
Mock
COVID
HC
COVID
HC
COVID
HC
siCtrl
siTLR7
siTLR8
sEV
Mock
COVID
HC
COVID
HC
COVID
HC
TLR7 (121 kDa)
β-actin

TLR8 (110 kDa)

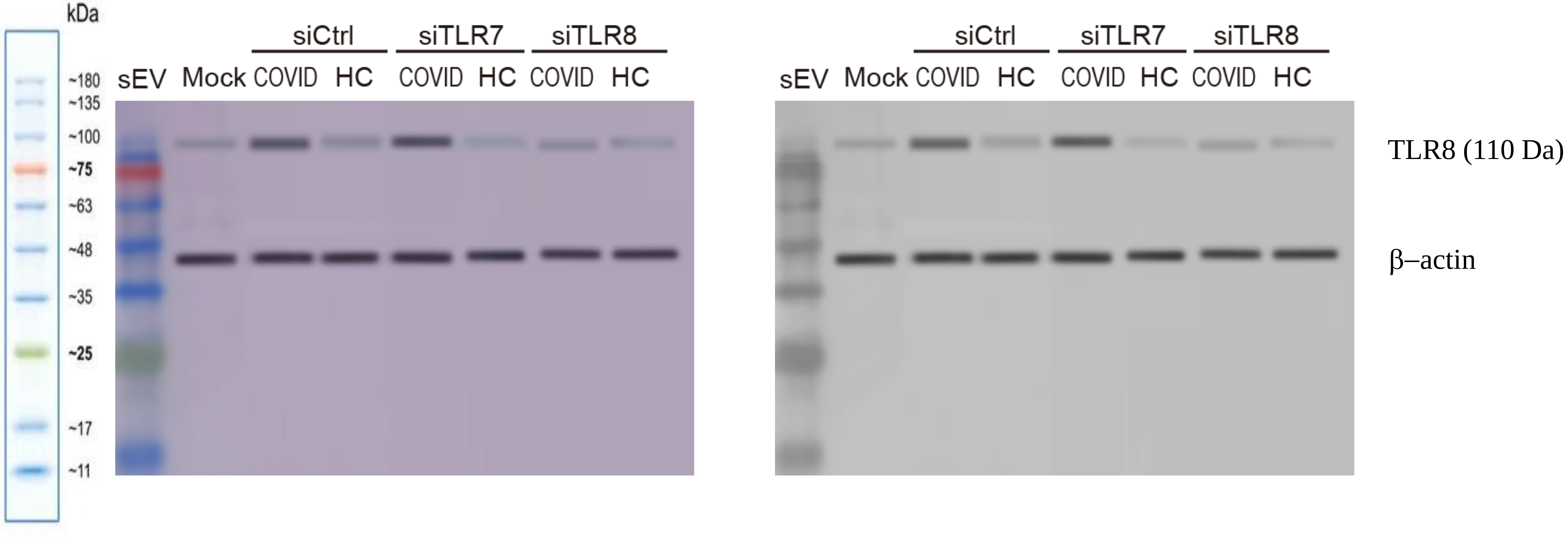
kDa
~180
~135
~100
~75
~63
~48
~35
~25
~17
~11
sEV
Mock
siCtrl
COVID
HC
siTLR7
COVID
HC
siTLR8
COVID
HC
sEV
Mock
siCtrl
COVID
HC
siTLR7
COVID
HC
siTLR8
COVID
HC
TLR8 (110 Da)
β–actin

TLR9 (113 kDa)

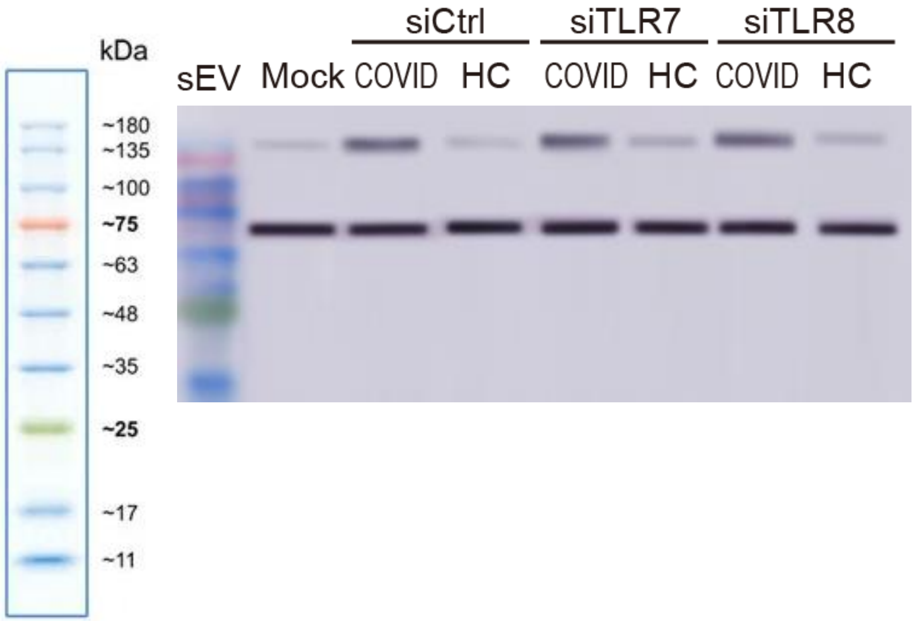

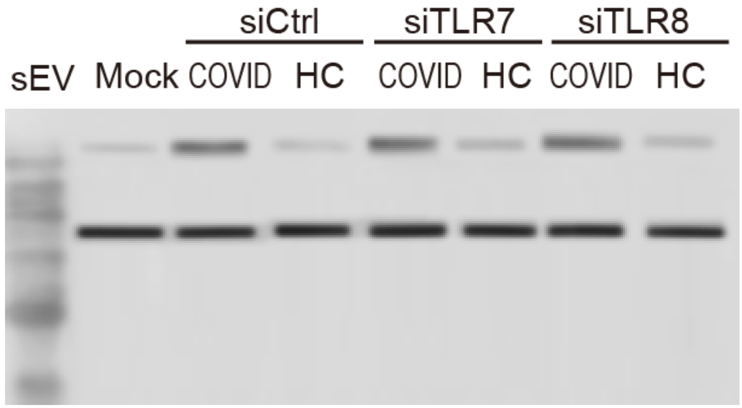

TLR9 (113 kDa)

β–actin

MPO (72 kDa)

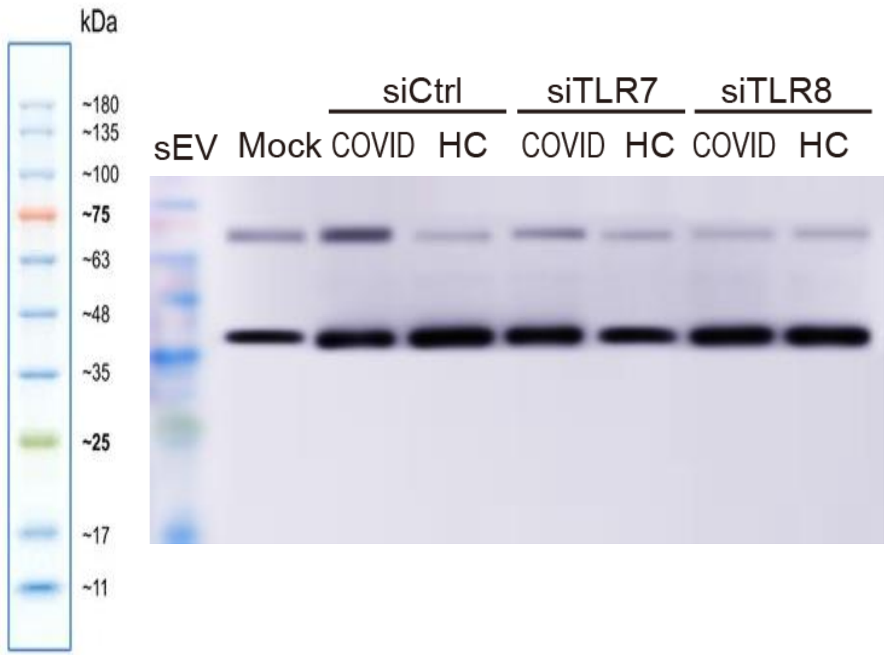
kDa
~180
~135
~100
~75
~63
~48
~35
~25
~17
~11
siCtrl
siTLR7
siTLR8
sEV
Mock
COVID
HC
COVID
HC
COVID
HC

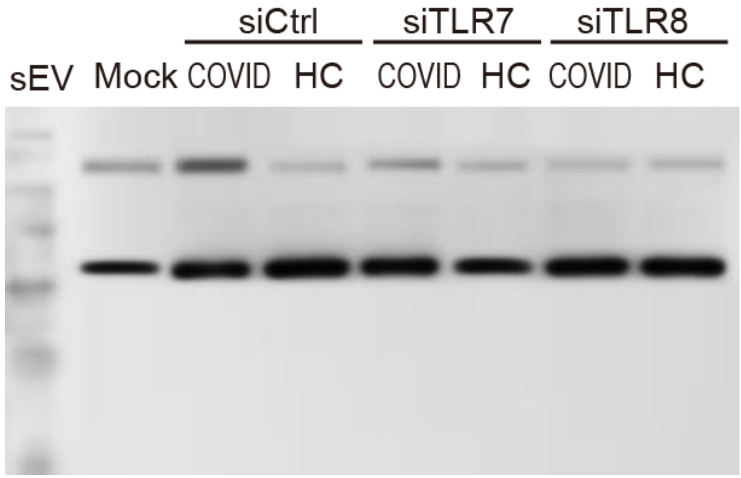
siCtrl
siTLR7
siTLR8
sEV
Mock
COVID
HC
COVID
HC
COVID
HC
MPO (72 kDa)
β–actin

PAD4 (72 kDa)

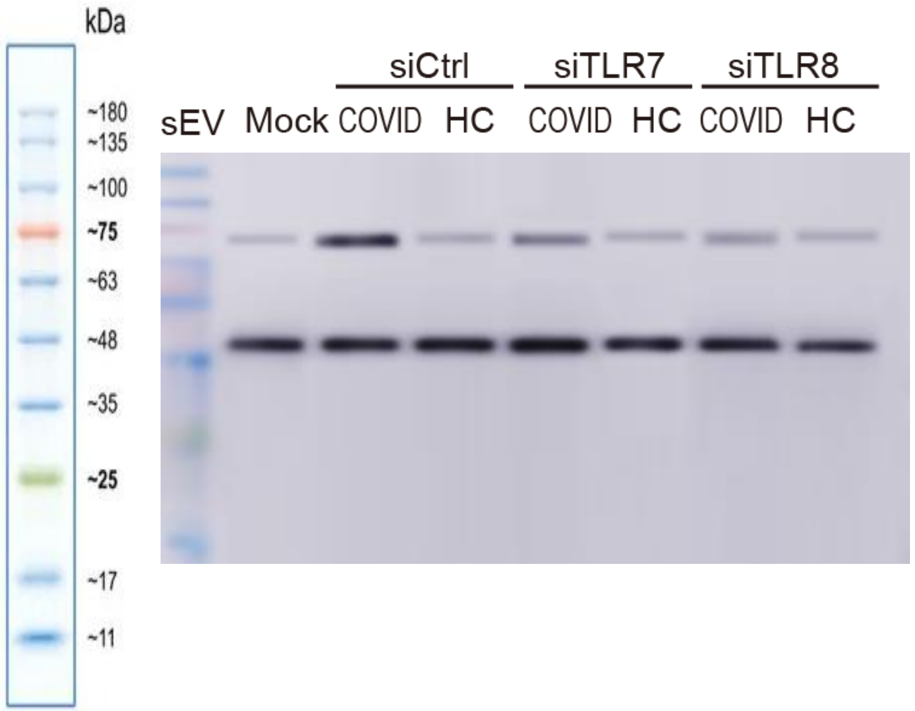

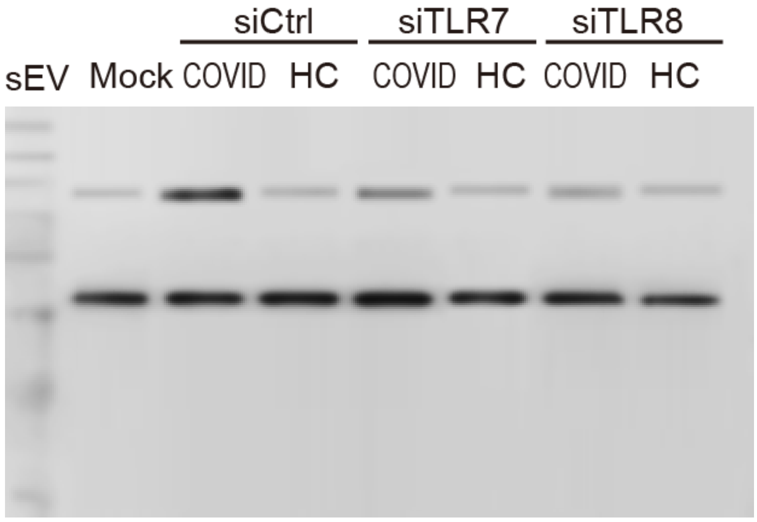

PAD4 (72 kDa)

β–actin

citH3 (14 kDa)

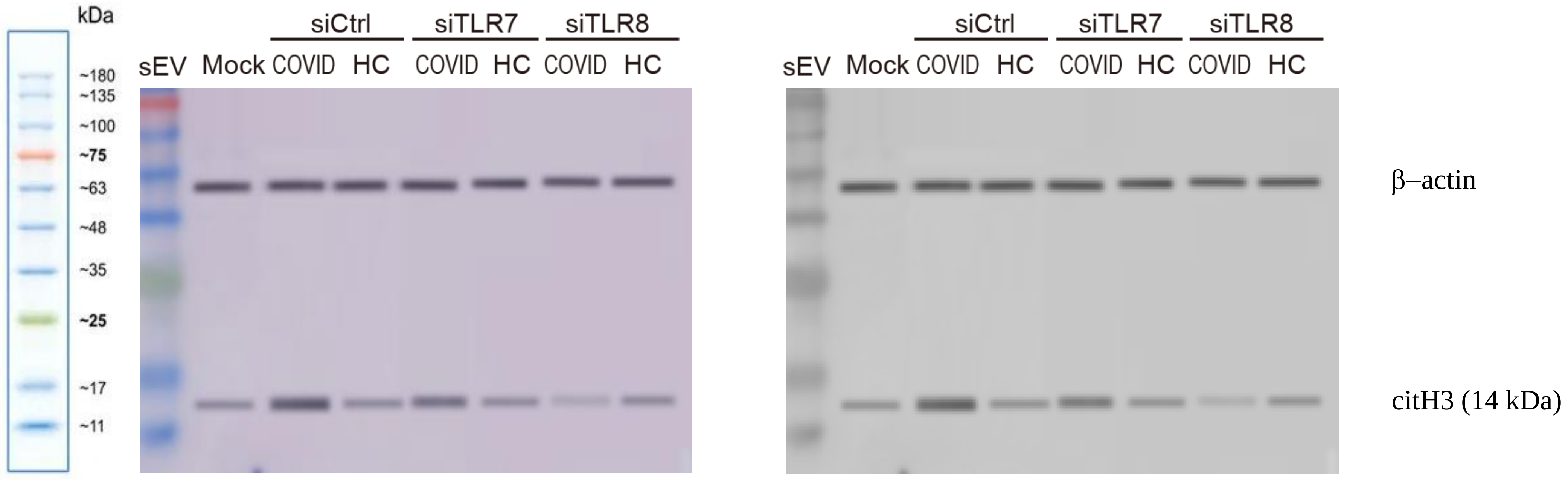
kDa
~180
~135
~100
~75
~63
~48
~35
~25
~17
~11
siCtrl
siTLR7
siTLR8
sEV
Mock
COVID
HC
COVID
HC
COVID
HC
siCtrl
siTLR7
siTLR8
sEV
Mock
COVID
HC
COVID
HC
COVID
HC
β–actin
citH3 (14 kDa)

Fig. 3C

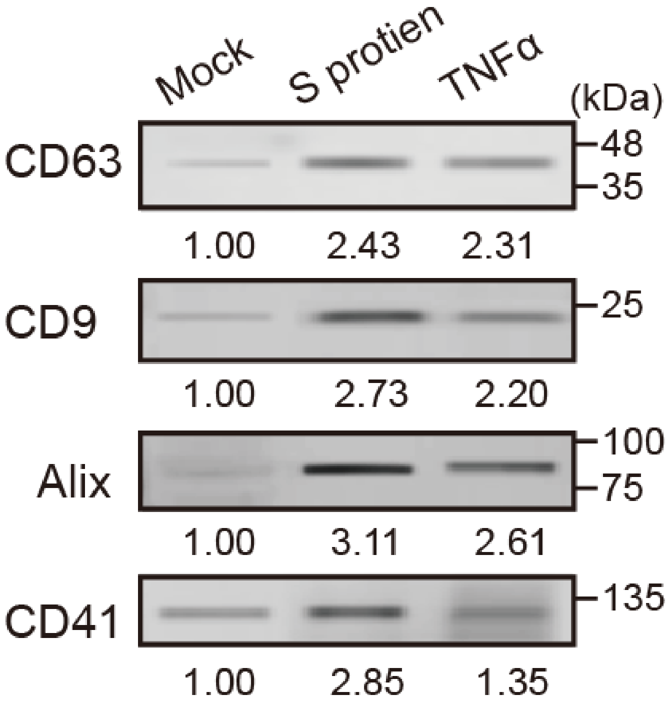
Mock
S protien
TNFα
(kDa)
CD63
48
35
1.00 2.43 2.31
CD9
25
1.00 2.73 2.20
Alix
100
75
1.00 3.11 2.61
CD41
135
1.00 2.85 1.35

CD63 (43 kDa)

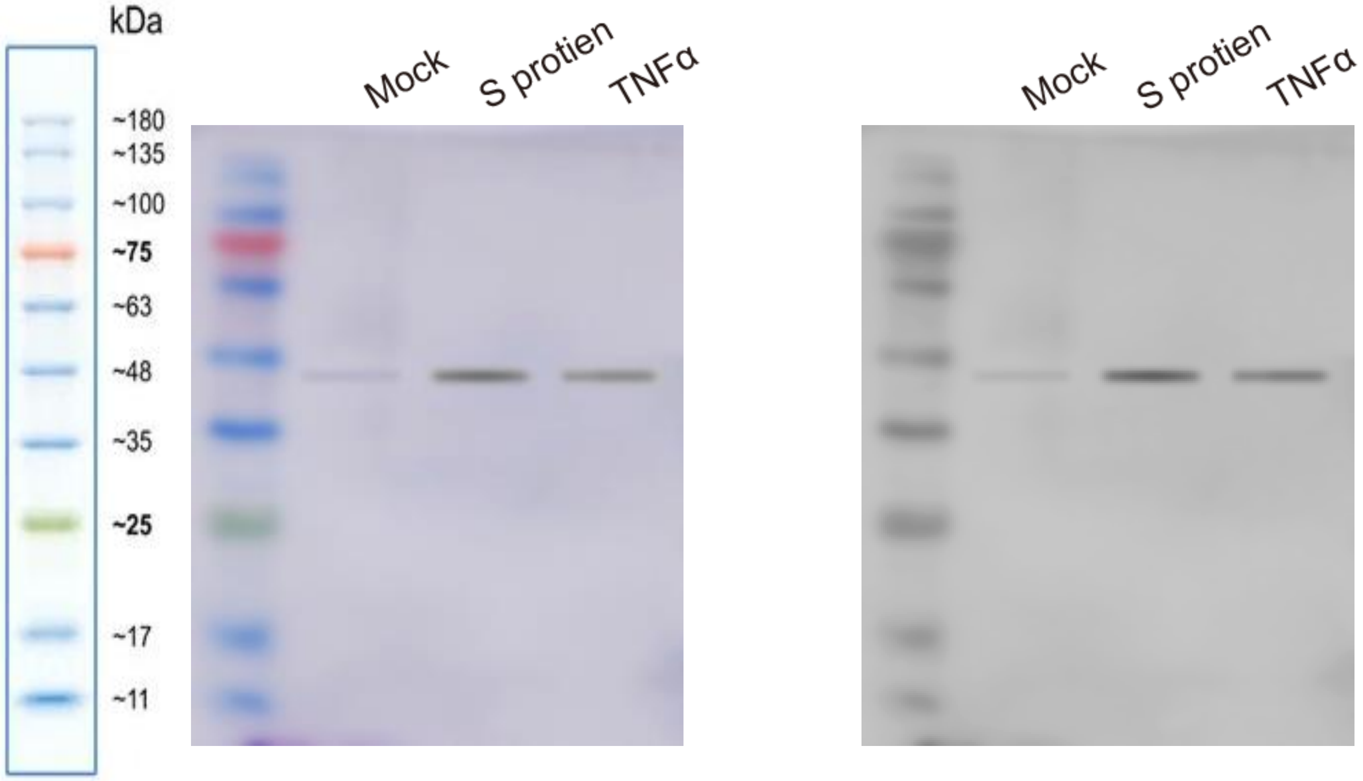

CD63 (43 kDa)

CD9 (24 kDa)

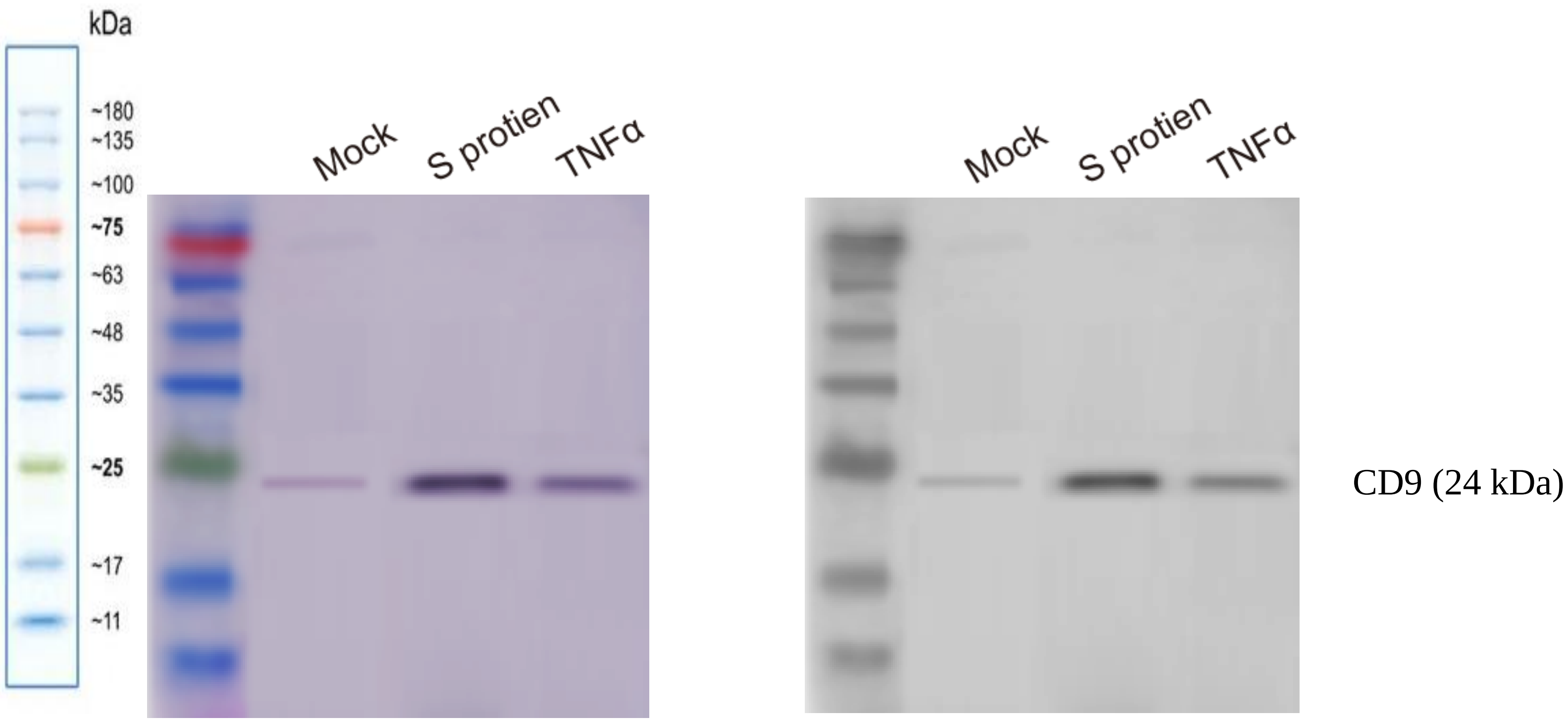
kDa
~180
~135
~100
~75
~63
~48
~35
~25
~17
~11
Mock
S protien
TNFα
Mock
S protien
TNFα
CD9 (24 kDa)

Alix (95 kDa)

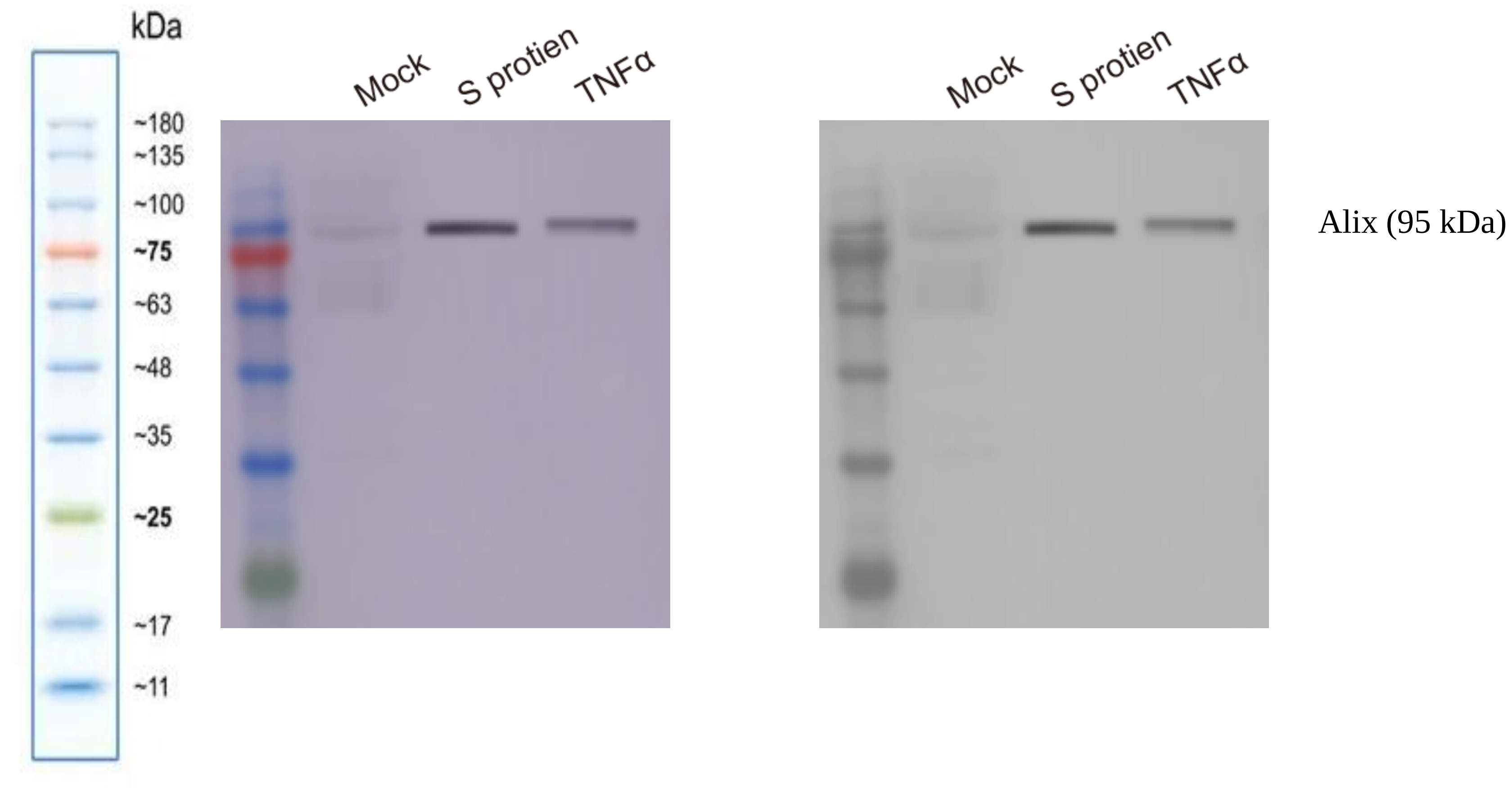
kDa
~180
~135
~100
~75
~63
~48
~35
~25
~17
~11
Mock
S protien
TNFα
Mock
S protien
TNFα
Alix (95 kDa)

CD41 (137 kDa)

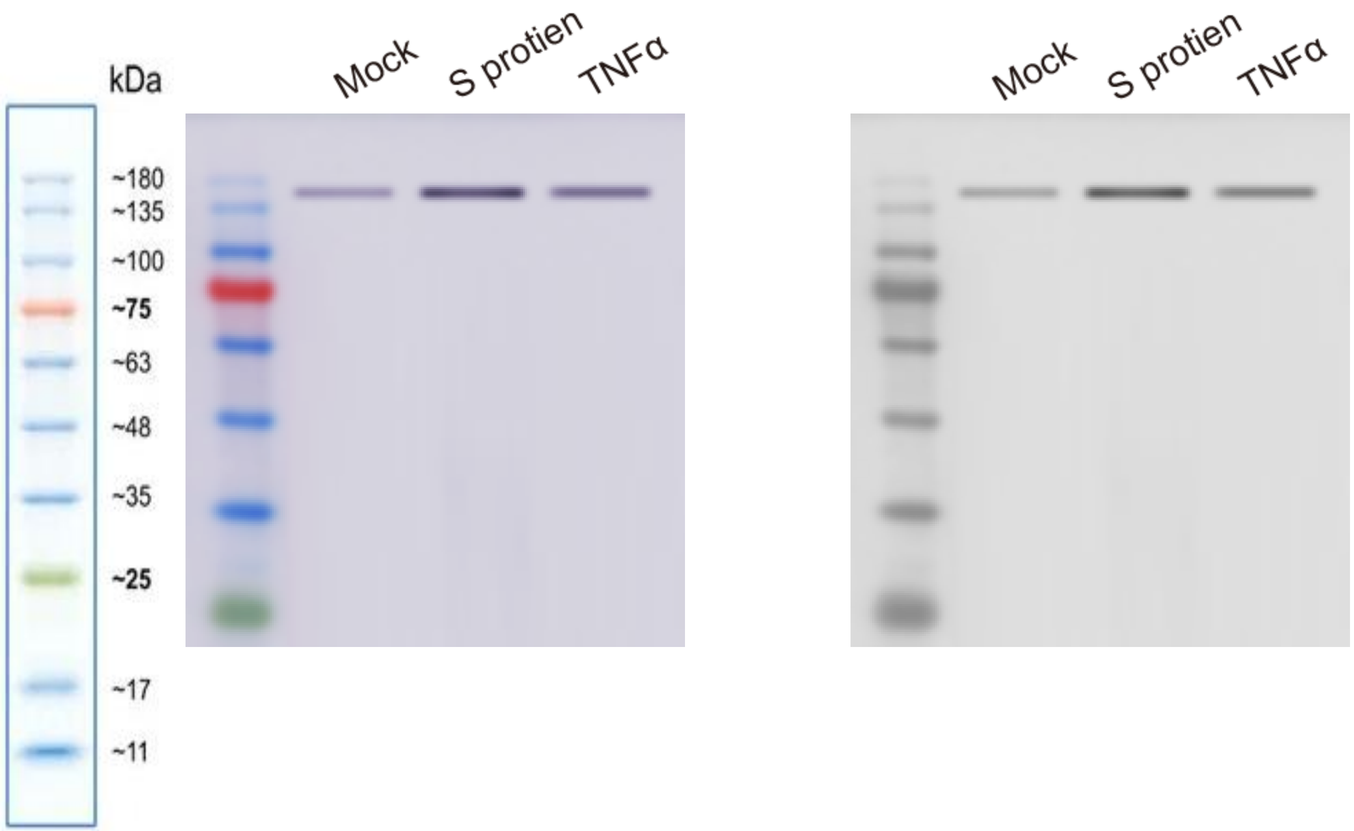

CD41 (137 kDa)

Fig. 3G

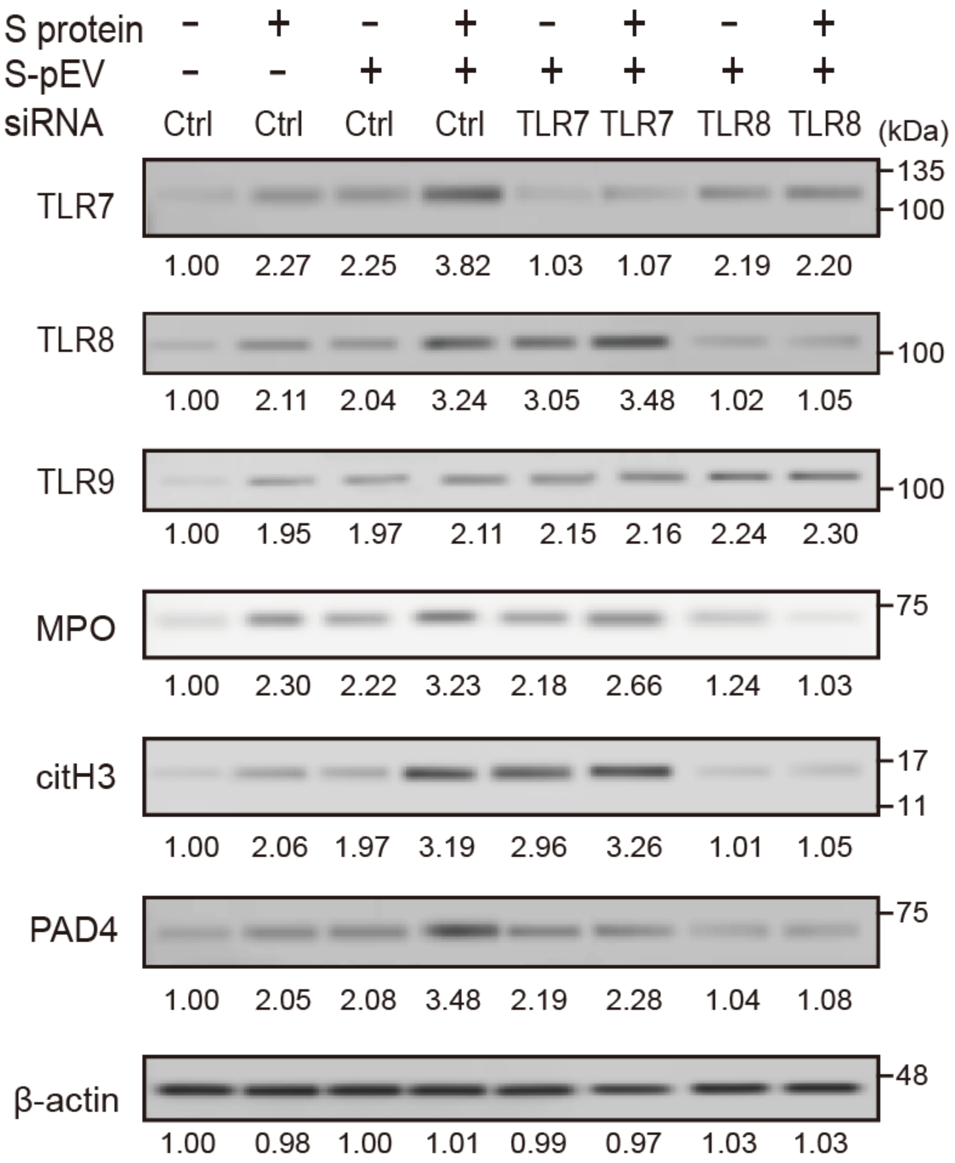
S protein - + - + - + - +
S-pEV - - + + + + + +
siRNA Ctrl Ctrl Ctrl Ctrl TLR7 TLR7 TLR8 TLR8
(kDa)
TLR7
135
100
1.00 2.27 2.25 3.82 1.03 1.07 2.19 2.20
TLR8
100
1.00 2.11 2.04 3.24 3.05 3.48 1.02 1.05
TLR9
100
1.00 1.95 1.97 2.11 2.15 2.16 2.24 2.30
MPO
75
1.00 2.30 2.22 3.23 2.18 2.66 1.24 1.03
citH3
17
11
1.00 2.06 1.97 3.19 2.96 3.26 1.01 1.05
PAD4
75
1.00 2.05 2.08 3.48 2.19 2.28 1.04 1.08
β-actin
48
1.00 0.98 1.00 1.01 0.99 0.97 1.03 1.03

TLR7 (121 kDa)

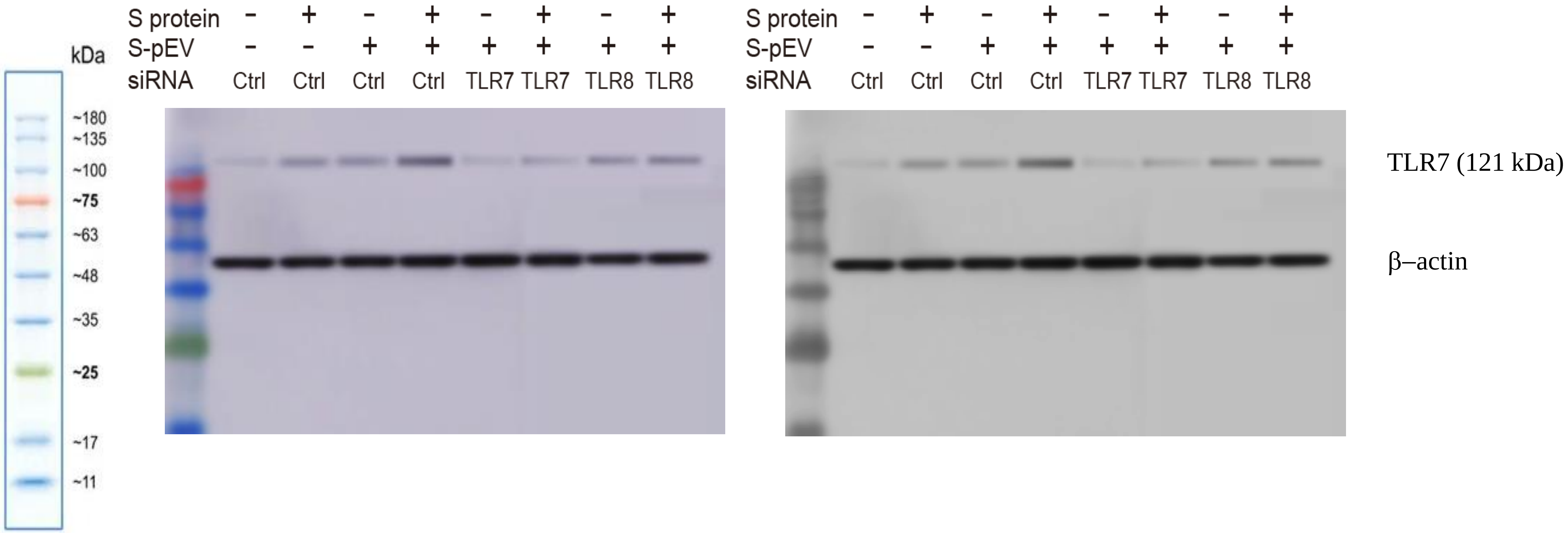
kDa
~180
~135
~100
~75
~63
~48
~35
~25
~17
~11
S protein - + - + - + - +
S-pEV - - + + + + + +
siRNA Ctrl Ctrl Ctrl Ctrl TLR7 TLR7 TLR8 TLR8
S protein - + - + - + - +
S-pEV - - + + + + + +
siRNA Ctrl Ctrl Ctrl Ctrl TLR7 TLR7 TLR8 TLR8
TLR7 (121 kDa)
β–actin

TLR8 (110 kDa)

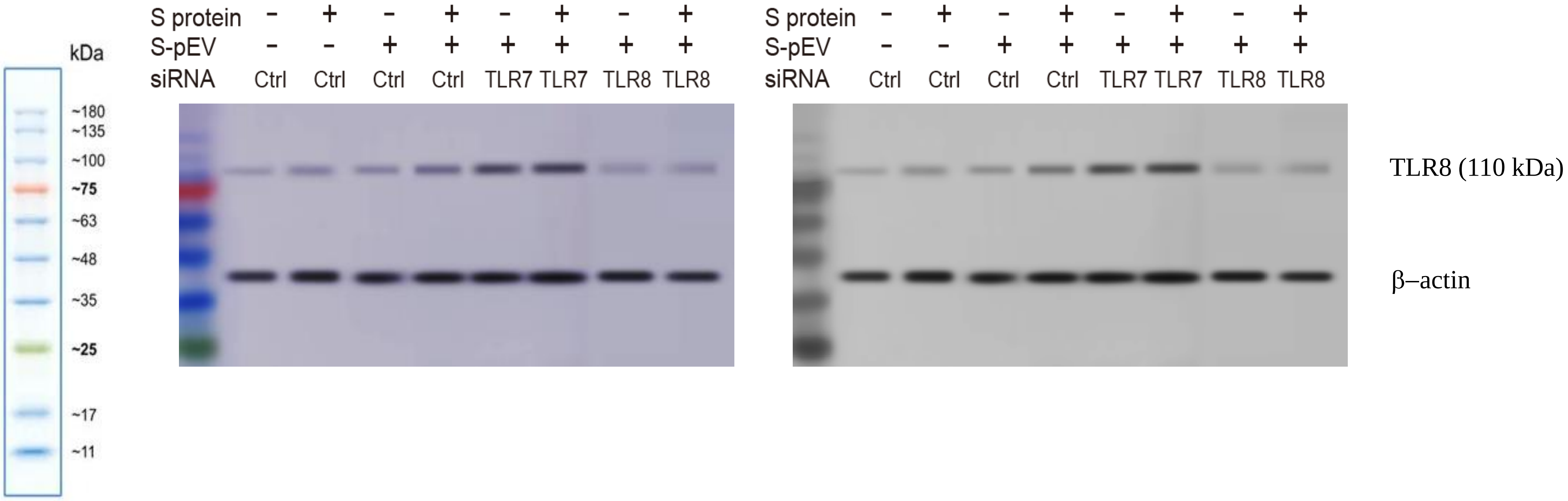
kDa
~180
~135
~100
~75
~63
~48
~35
~25
~17
~11
S protein - + - + - + - +
S-pEV - - + + + + + +
siRNA Ctrl Ctrl Ctrl Ctrl TLR7 TLR7 TLR8 TLR8
S protein - + - + - + - +
S-pEV - - + + + + + +
siRNA Ctrl Ctrl Ctrl Ctrl TLR7 TLR7 TLR8 TLR8
TLR8 (110 kDa)
β–actin

TLR9 (113 kDa)

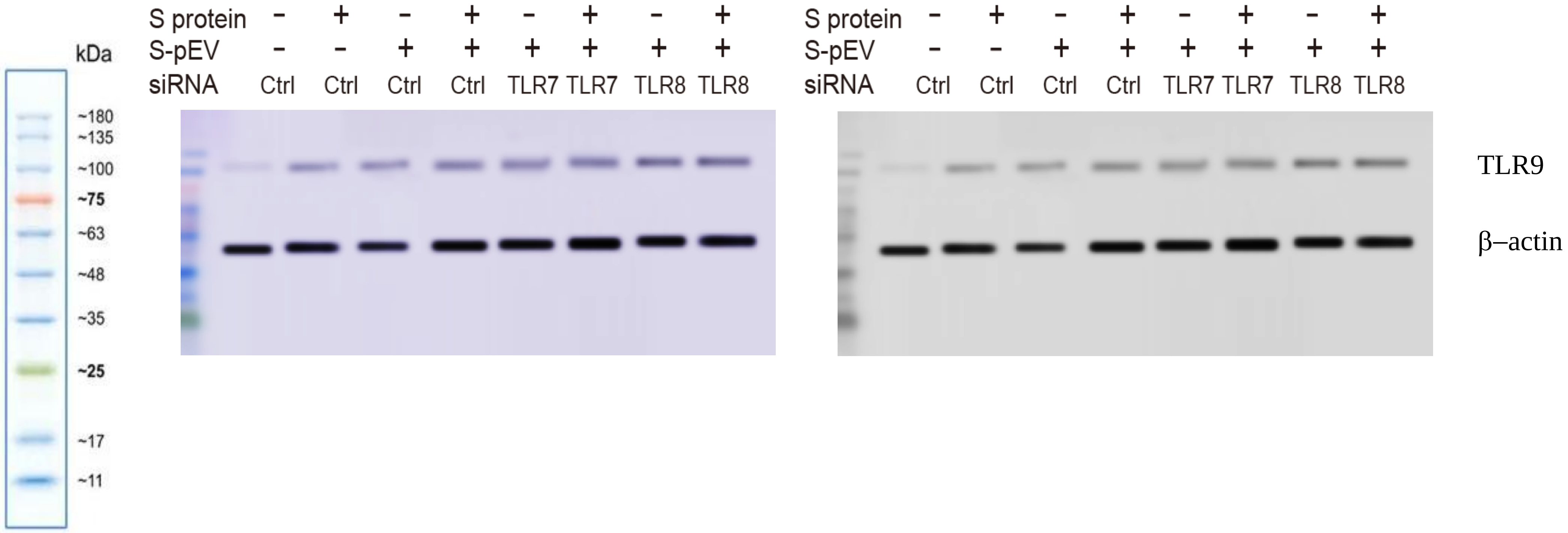
kDa
~180
~135
~100
~75
~63
~48
~35
~25
~17
~11
S protein - + - + - + - +
S-pEV - - + + + + + +
siRNA Ctrl Ctrl Ctrl Ctrl TLR7 TLR7 TLR8 TLR8
S protein - + - + - + - +
S-pEV - - + + + + + +
siRNA Ctrl Ctrl Ctrl Ctrl TLR7 TLR7 TLR8 TLR8
TLR9
β–actin

MPO (72 kDa)

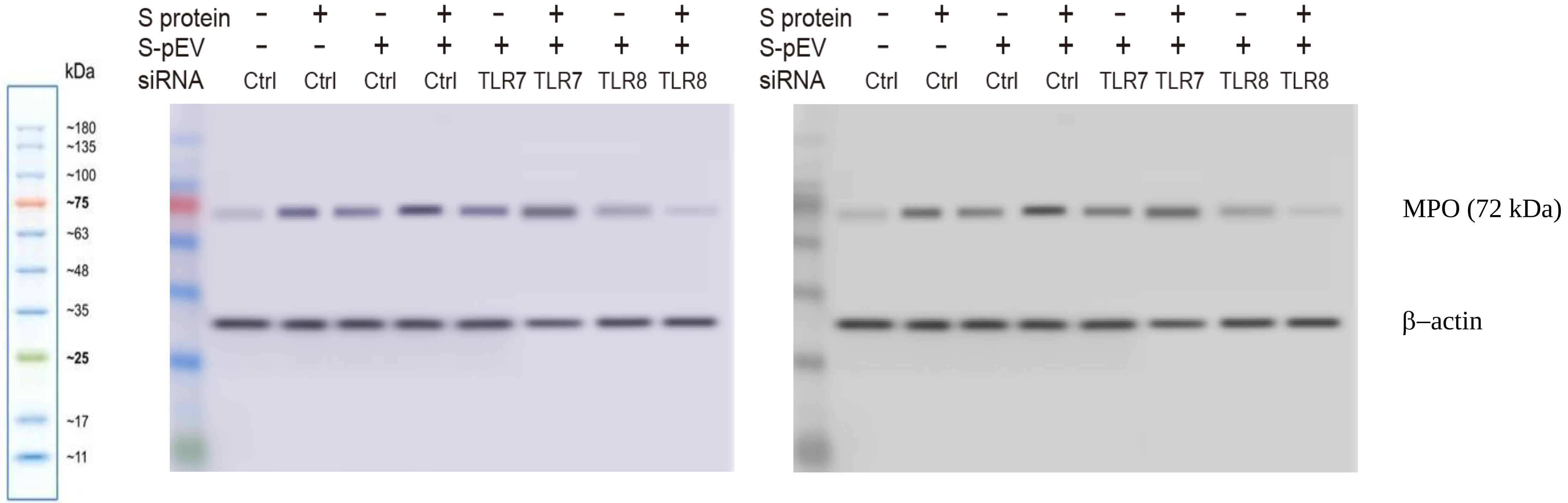

kDa
~180
~135
~100
~75
~63
~48
~35
~25
~17
~11
S protein - + - + - + - +
S-pEV - - + + + + + +
siRNA Ctrl Ctrl Ctrl Ctrl TLR7 TLR7 TLR8 TLR8
S protein - + - + - + - +
S-pEV - - + + + + + +
siRNA Ctrl Ctrl Ctrl Ctrl TLR7 TLR7 TLR8 TLR8
MPO (72 kDa)
β–actin

citH3 (14 kDa)

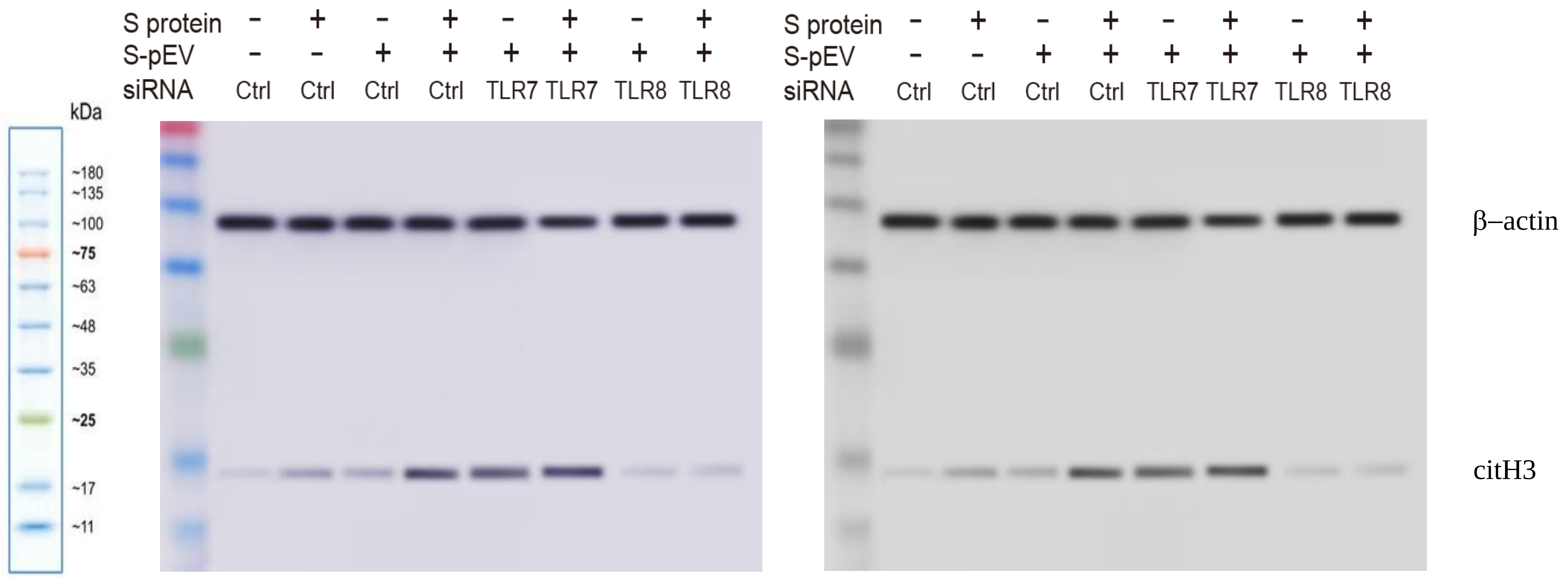
S protein - + - + - + - +
S-pEV - - + + + + + +
siRNA Ctrl Ctrl Ctrl Ctrl TLR7 TLR7 TLR8 TLR8
kDa
~180
~135
~100
~75
~63
~48
~35
~25
~17
~11
S protein - + - + - + - +
S-pEV - - + + + + + +
siRNA Ctrl Ctrl Ctrl Ctrl TLR7 TLR7 TLR8 TLR8
β–actin
citH3

PAD4(72 kDa)

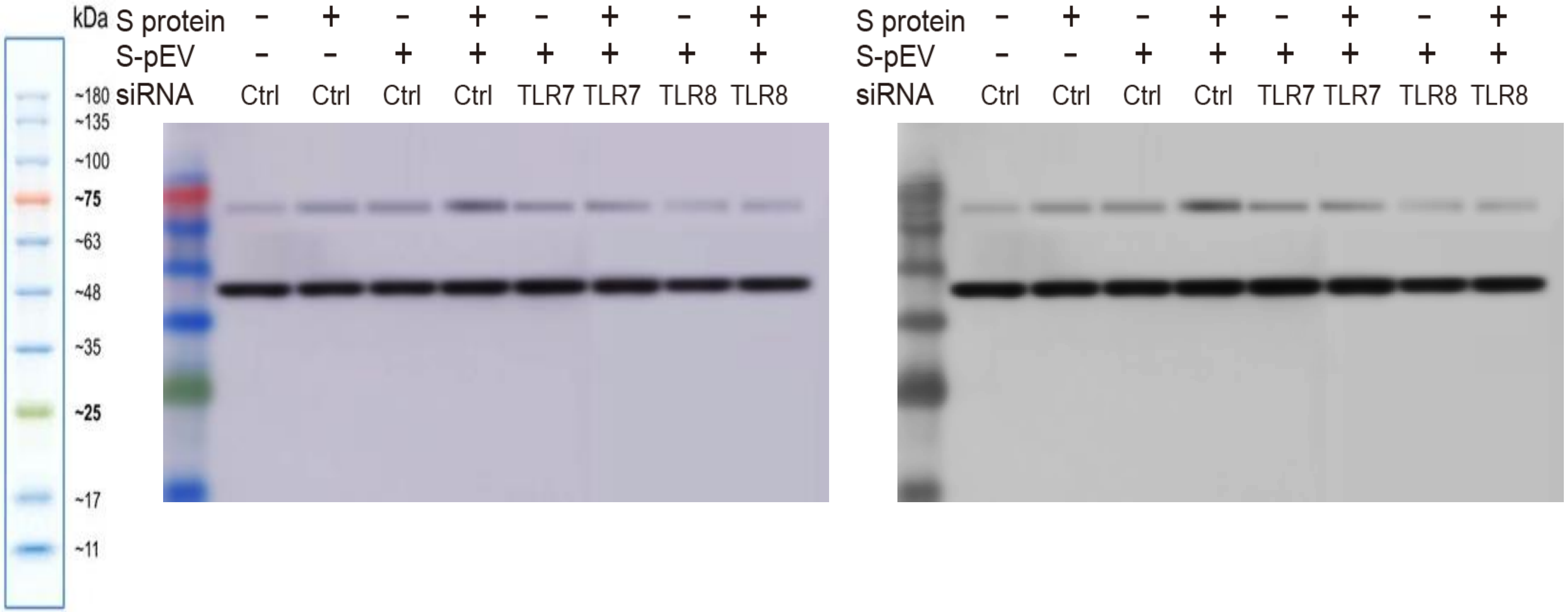

PAD4(72 kDa)

β–actin

Fig. 5E

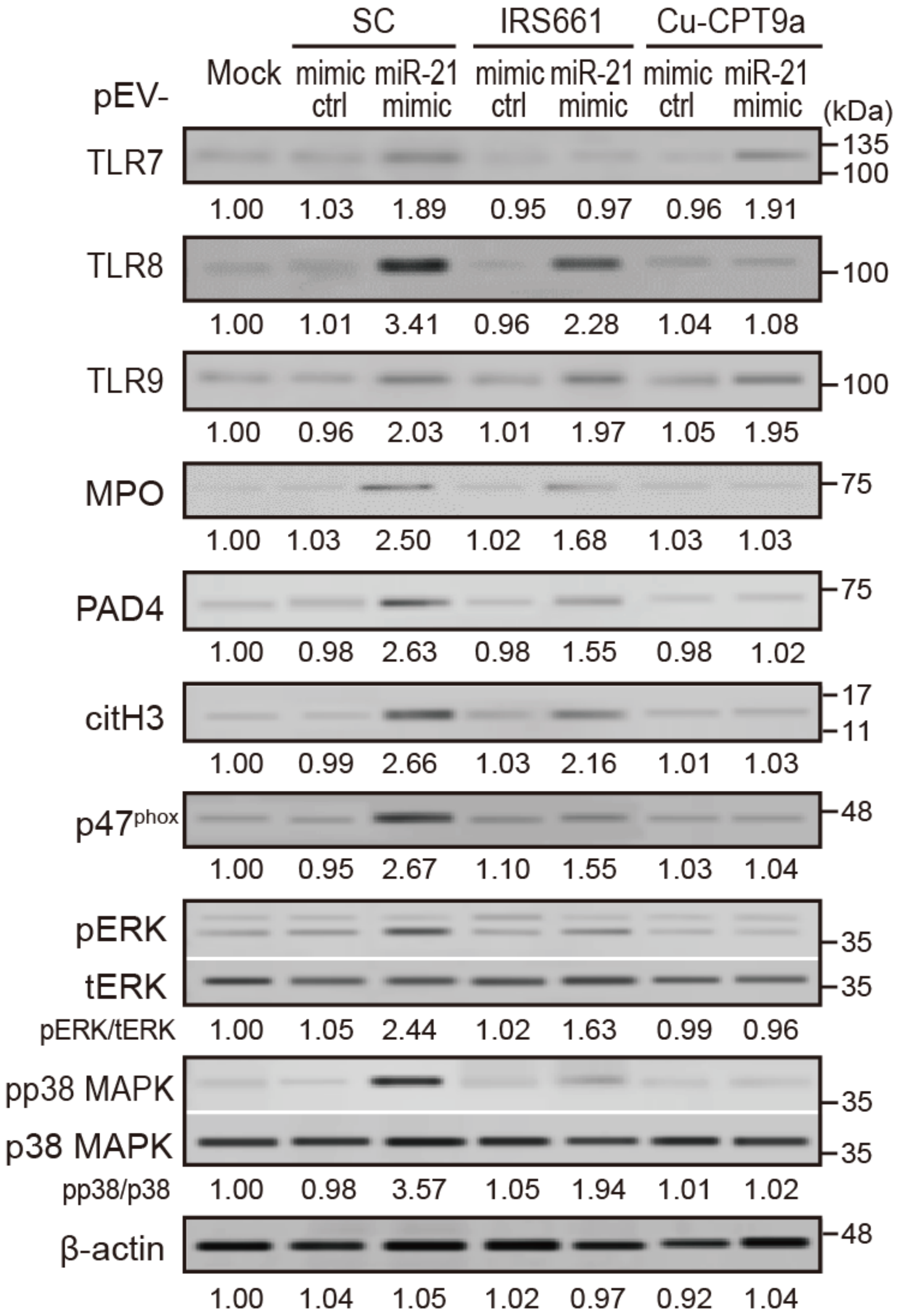

SC
IRS661
Cu-CPT9a
pEV-
Mock
mimic ctrl
miR-21 mimic
mimic ctrl
miR-21 mimic
mimic ctrl
miR-21 mimic
(kDa)
TLR7
135
100
1.00 1.03 1.89 0.95 0.97 0.96 1.91
TLR8
100
1.00 1.01 3.41 0.96 2.28 1.04 1.08
TLR9
100
1.00 0.96 2.03 1.01 1.97 1.05 1.95
MPO
75
1.00 1.03 2.50 1.02 1.68 1.03 1.03
PAD4
75
1.00 0.98 2.63 0.98 1.55 0.98 1.02
citH3
17
11
1.00 0.99 2.66 1.03 2.16 1.01 1.03
p47phox
48
1.00 0.95 2.67 1.10 1.55 1.03 1.04
pERK
35
tERK
35
pERK/tERK 1.00 1.05 2.44 1.02 1.63 0.99 0.96
pp38 MAPK
35
p38 MAPK
35
pp38/p38 1.00 0.98 3.57 1.05 1.94 1.01 1.02
β-actin
48
1.00 1.04 1.05 1.02 0.97 0.92 1.04

TLR7 (121 kDa)

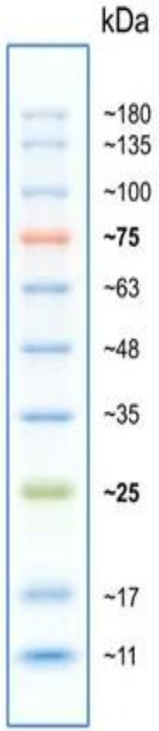

kDa
~180
~135
~100
~75
~63
~48
~35
~25
~17
~11

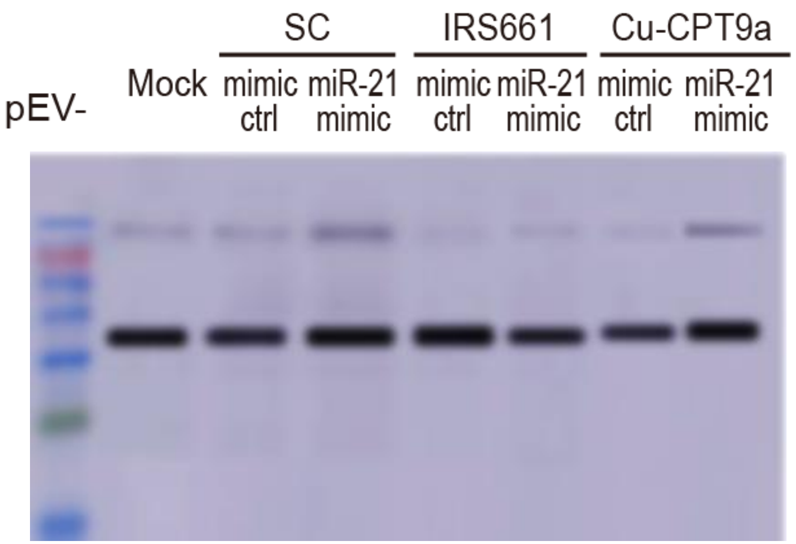

SC
IRS661
Cu-CPT9a
pEV-
Mock
mimic ctrl
miR-21 mimic
mimic ctrl
miR-21 mimic
mimic ctrl
miR-21 mimic

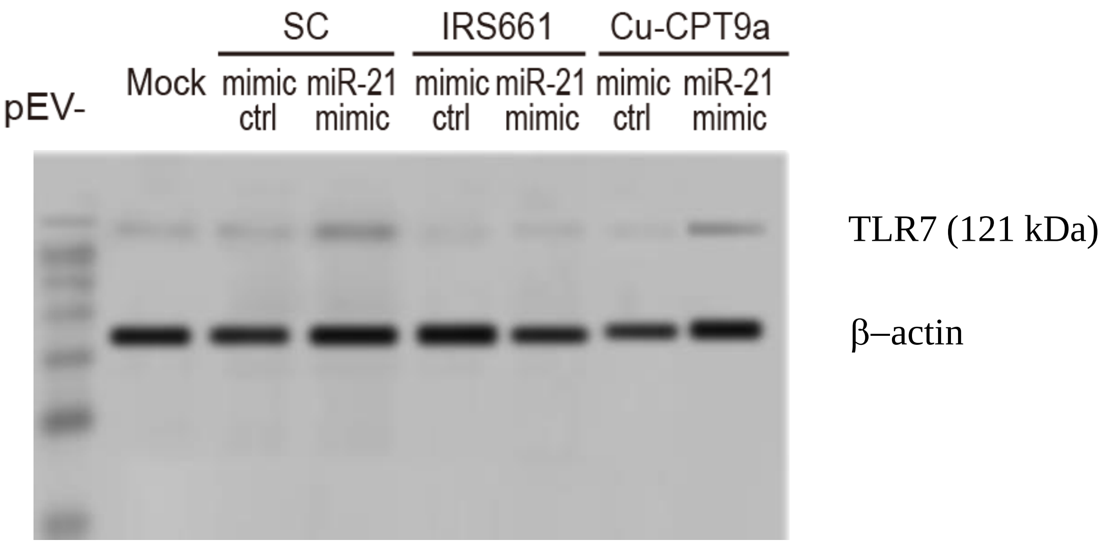

SC
IRS661
Cu-CPT9a
pEV-
Mock
mimic ctrl
miR-21 mimic
mimic ctrl
miR-21 mimic
mimic ctrl
miR-21 mimic
TLR7 (121 kDa)
β–actin

TLR8 (110 kDa)

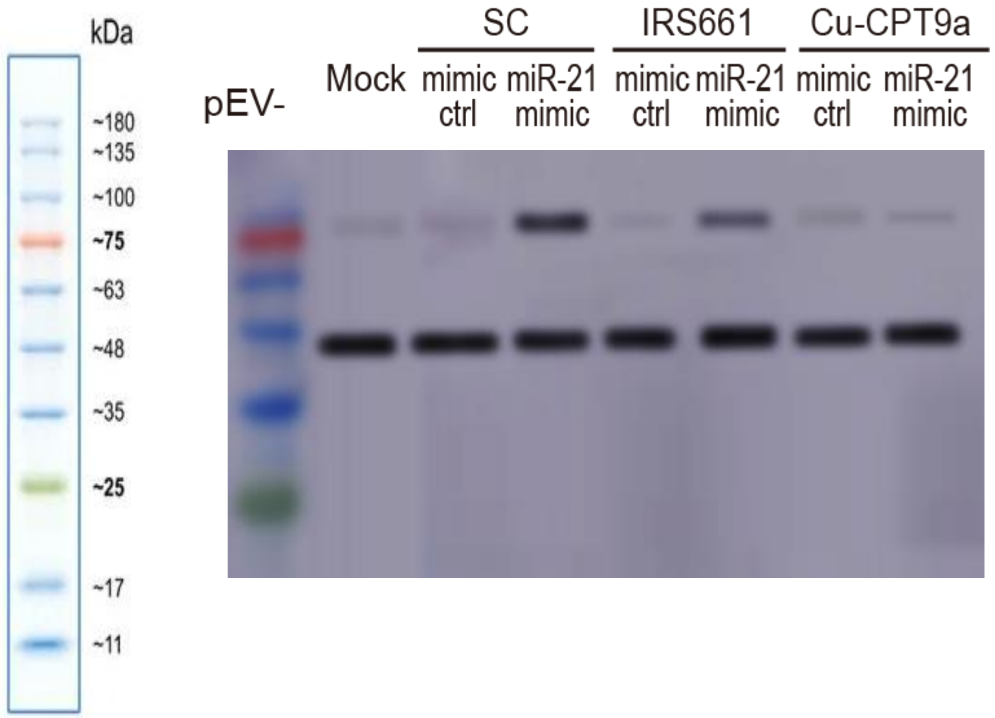

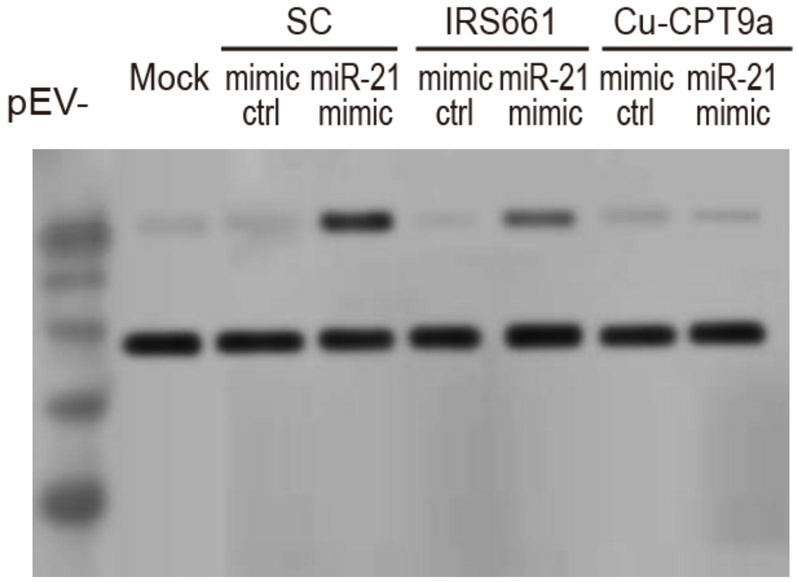

TLR8 (110 kDa)

β–actin

TLR9 (113 kDa)

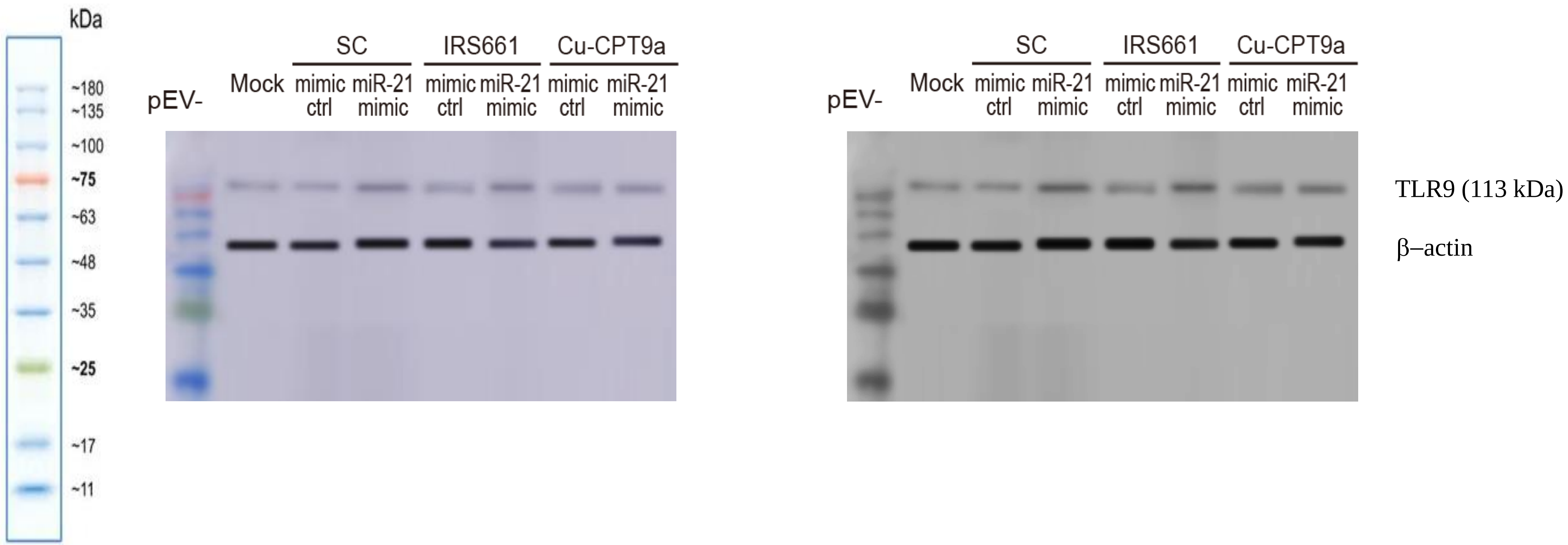
kDa
~180
~135
~100
~75
~63
~48
~35
~25
~17
~11
pEV-
Mock
SC
mimic ctrl
miR-21 mimic
IRS661
mimic ctrl
miR-21 mimic
Cu-CPT9a
mimic ctrl
miR-21 mimic
pEV-
Mock
SC
mimic ctrl
miR-21 mimic
IRS661
mimic ctrl
miR-21 mimic
Cu-CPT9a
mimic ctrl
miR-21 mimic
TLR9 (113 kDa)
β–actin

MPO (72 kDa)

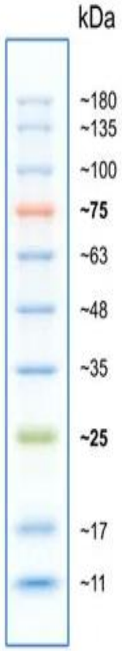
kDa
~180
~135
~100
~75
~63
~48
~35
~25
~17
~11

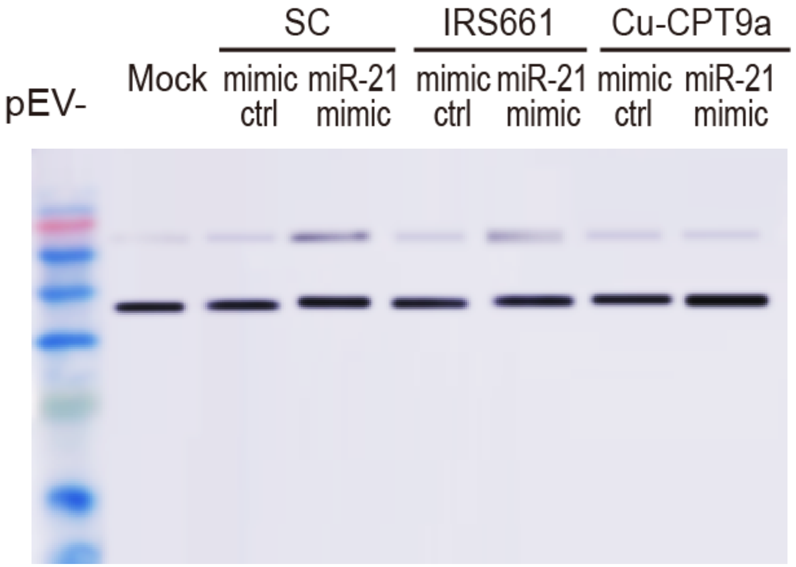
SC
IRS661
Cu-CPT9a
pEV-
Mock
mimic ctrl
miR-21 mimic
mimic ctrl
miR-21 mimic
mimic ctrl
miR-21 mimic

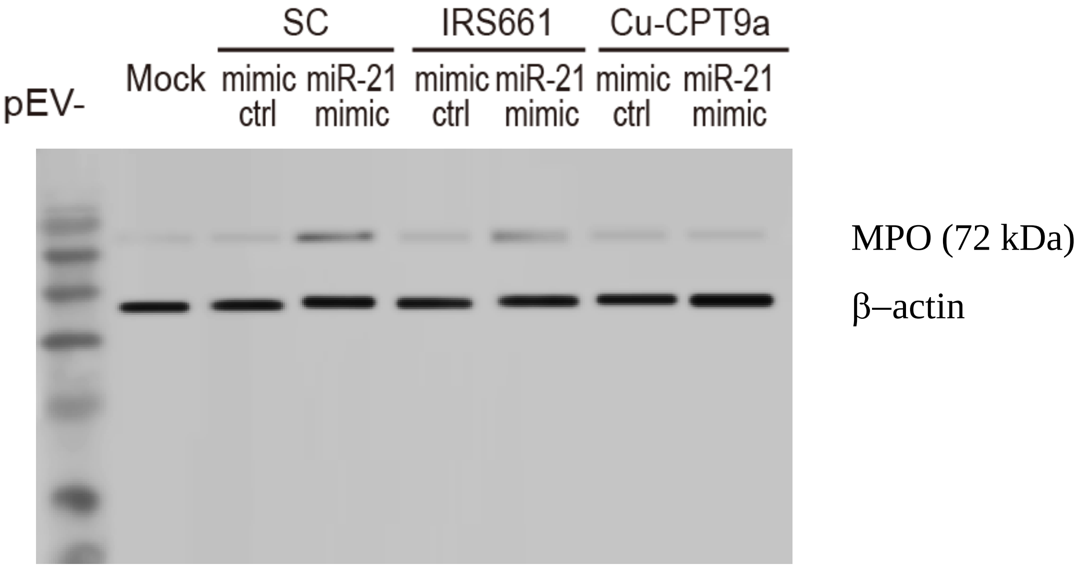
SC
IRS661
Cu-CPT9a
pEV-
Mock
mimic ctrl
miR-21 mimic
mimic ctrl
miR-21 mimic
mimic ctrl
miR-21 mimic
MPO (72 kDa)
β–actin

PAD4(72 kDa)

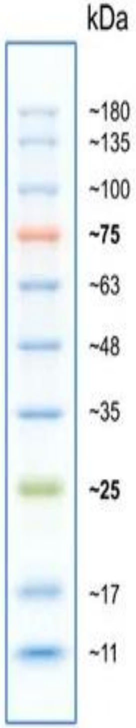
kDa
~180
~135
~100
~75
~63
~48
~35
~25
~17
~11

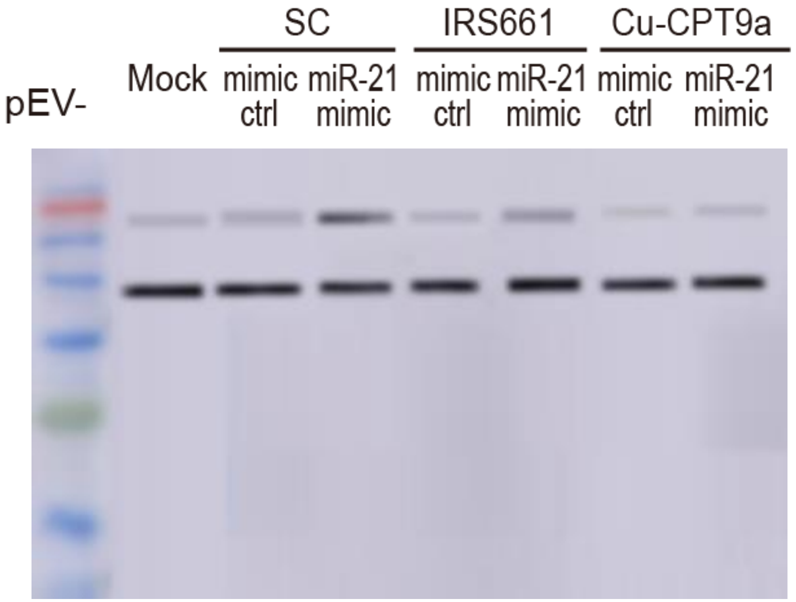
SC
IRS661
Cu-CPT9a
pEV-
Mock
mimic ctrl
miR-21 mimic
mimic ctrl
miR-21 mimic
mimic ctrl
miR-21 mimic

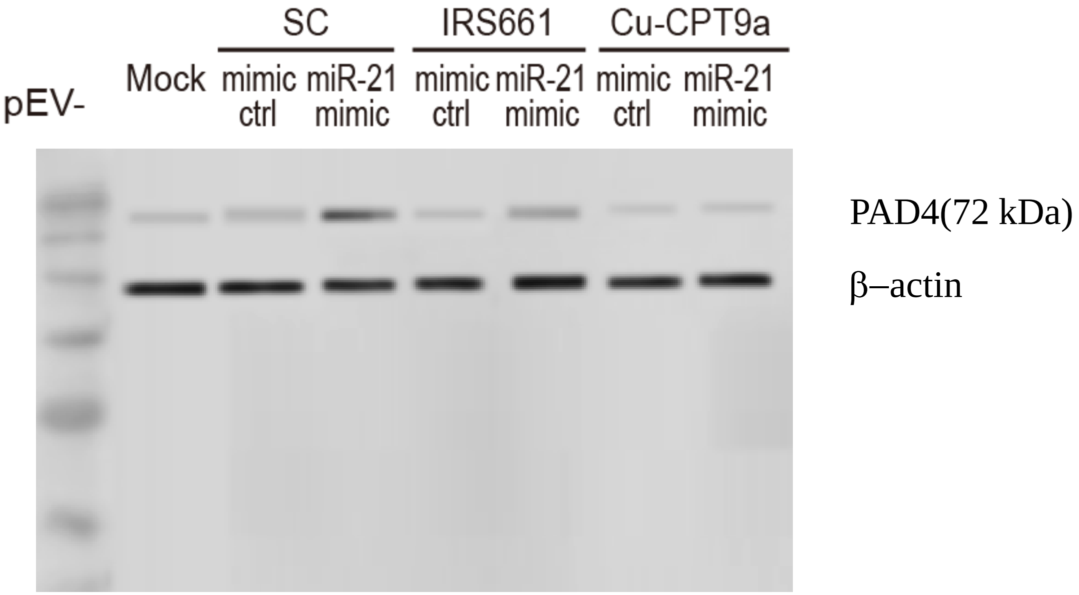
SC
IRS661
Cu-CPT9a
pEV-
Mock
mimic ctrl
miR-21 mimic
mimic ctrl
miR-21 mimic
mimic ctrl
miR-21 mimic
PAD4(72 kDa)
β–actin

citH3 (14 kDa)

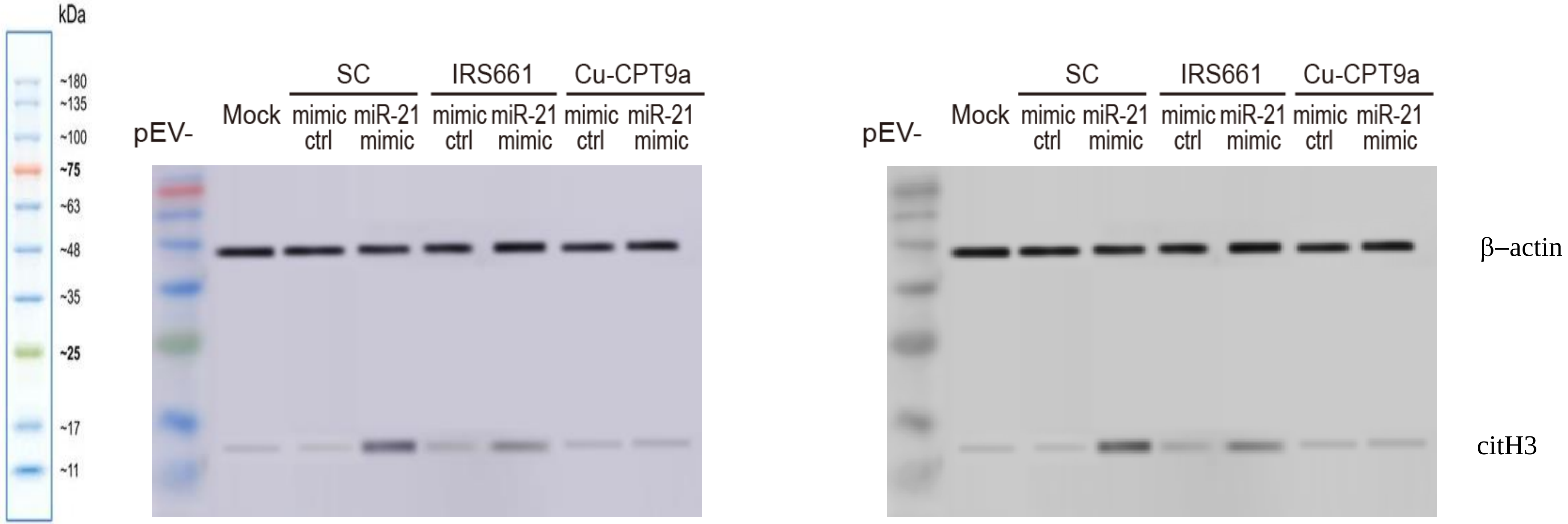

kDa
~180
~135
~100
~75
~63
~48
~35
~25
~17
~11
SC
IRS661
Cu-CPT9a
pEV-
Mock
mimic ctrl
miR-21 mimic
mimic ctrl
miR-21 mimic
mimic ctrl
miR-21 mimic
SC
IRS661
Cu-CPT9a
pEV-
Mock
mimic ctrl
miR-21 mimic
mimic ctrl
miR-21 mimic
mimic ctrl
miR-21 mimic
β–actin
citH3

p47 (47 kDa)

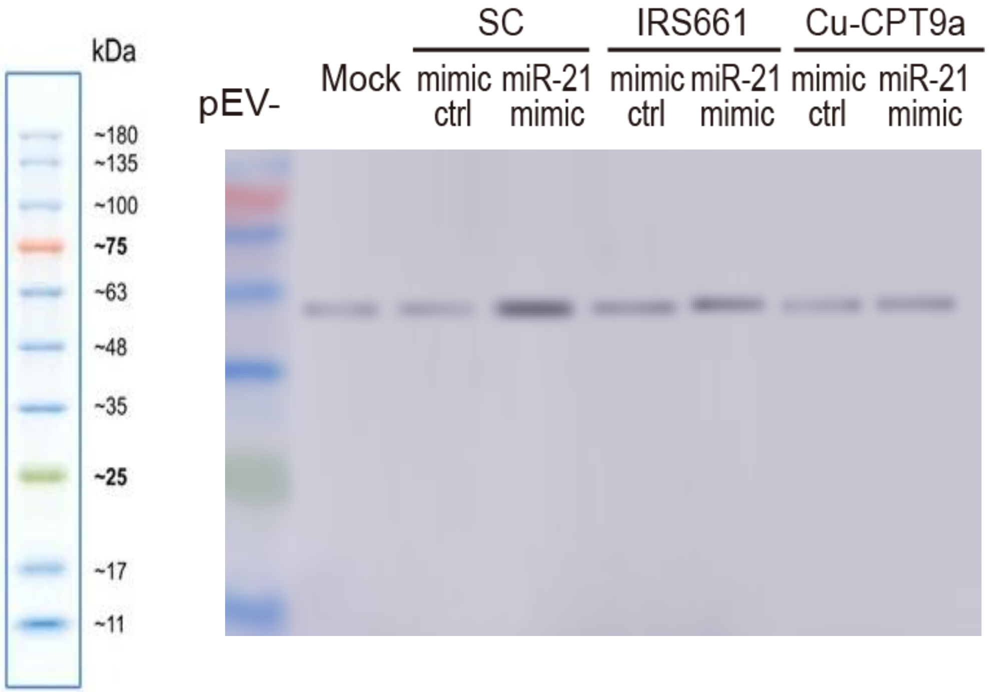

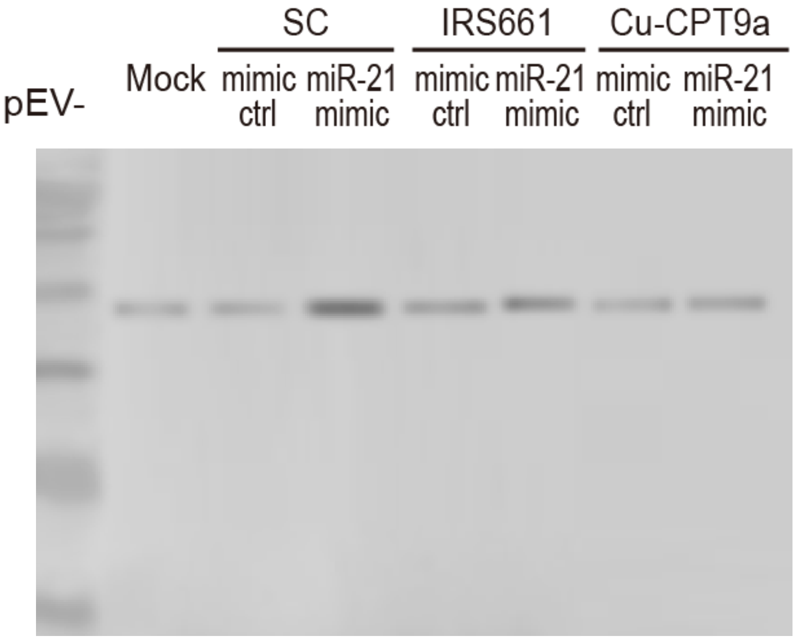

p47 (47 kDa)

pERK (42,44 kDa)

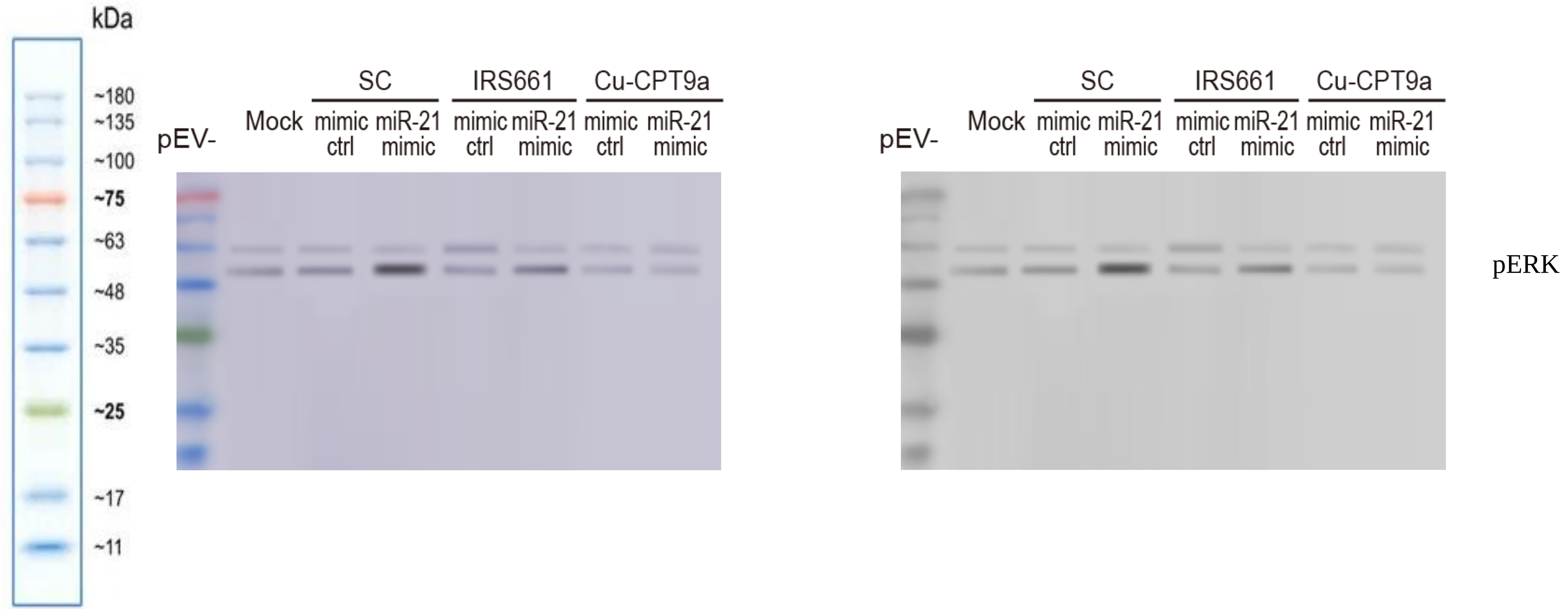

tERK (44 kDa)

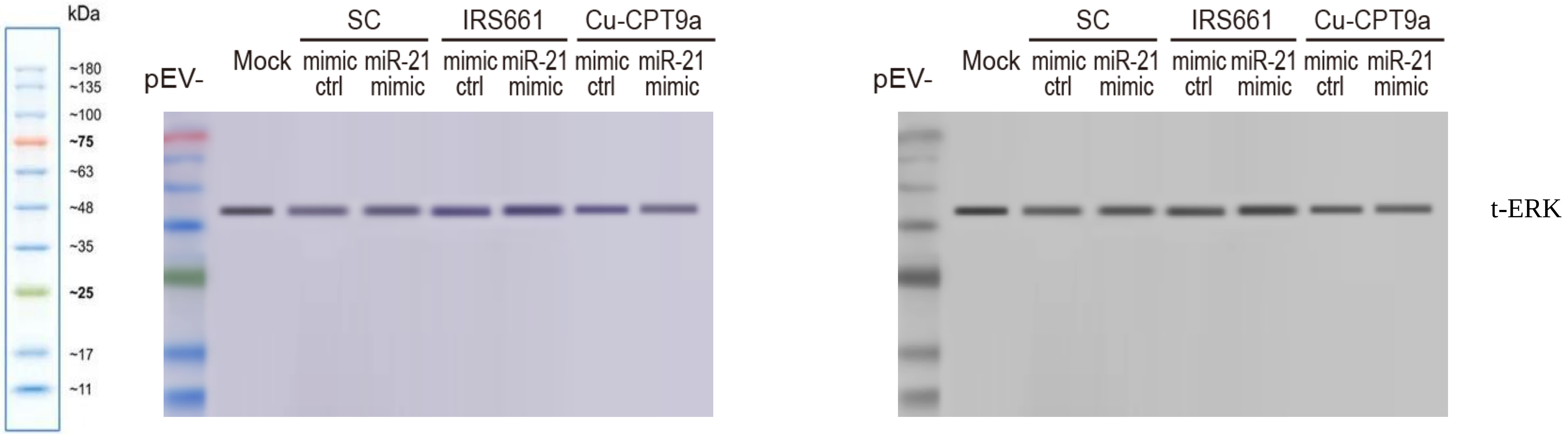
kDa
~180
~135
~100
~75
~63
~48
~35
~25
~17
~11
SC
IRS661
Cu-CPT9a
pEV-
Mock
mimic ctrl
miR-21 mimic
mimic ctrl
miR-21 mimic
mimic ctrl
miR-21 mimic
SC
IRS661
Cu-CPT9a
pEV-
Mock
mimic ctrl
miR-21 mimic
mimic ctrl
miR-21 mimic
mimic ctrl
miR-21 mimic
t-ERK

phospho-p38 MAPK (43 kDa)

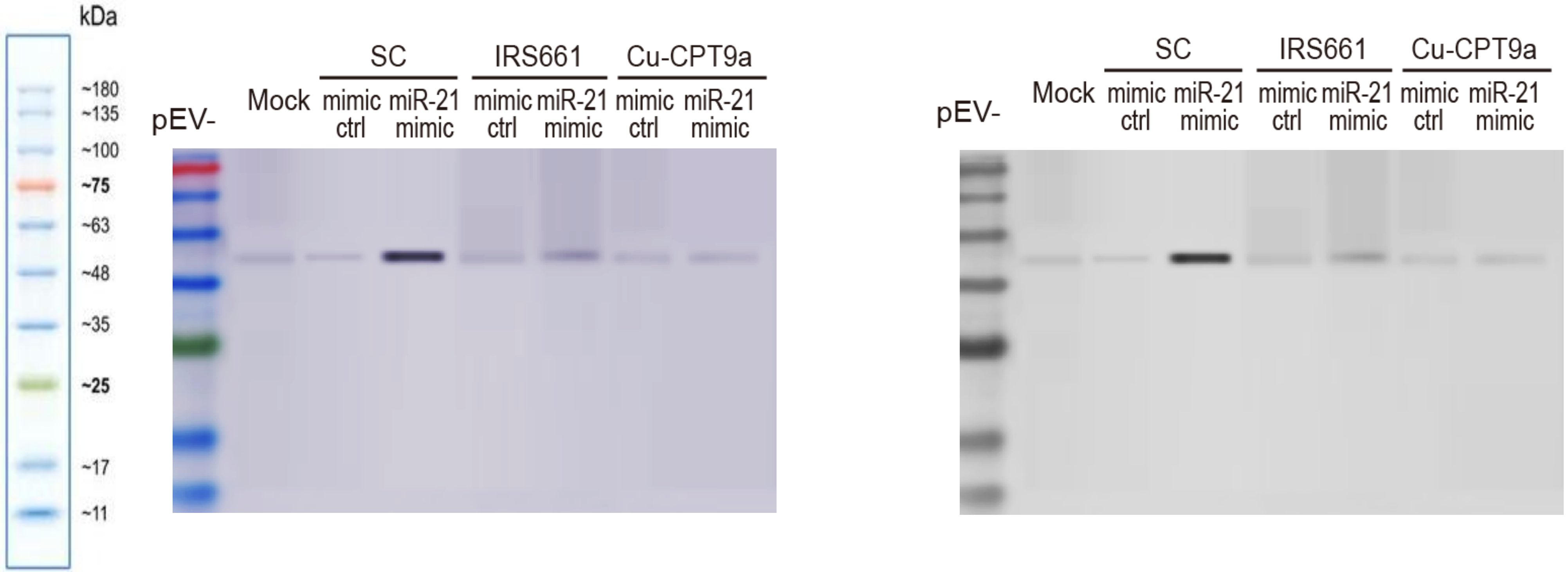

pp38 MAPK

p38 MAPK (40 kDa)

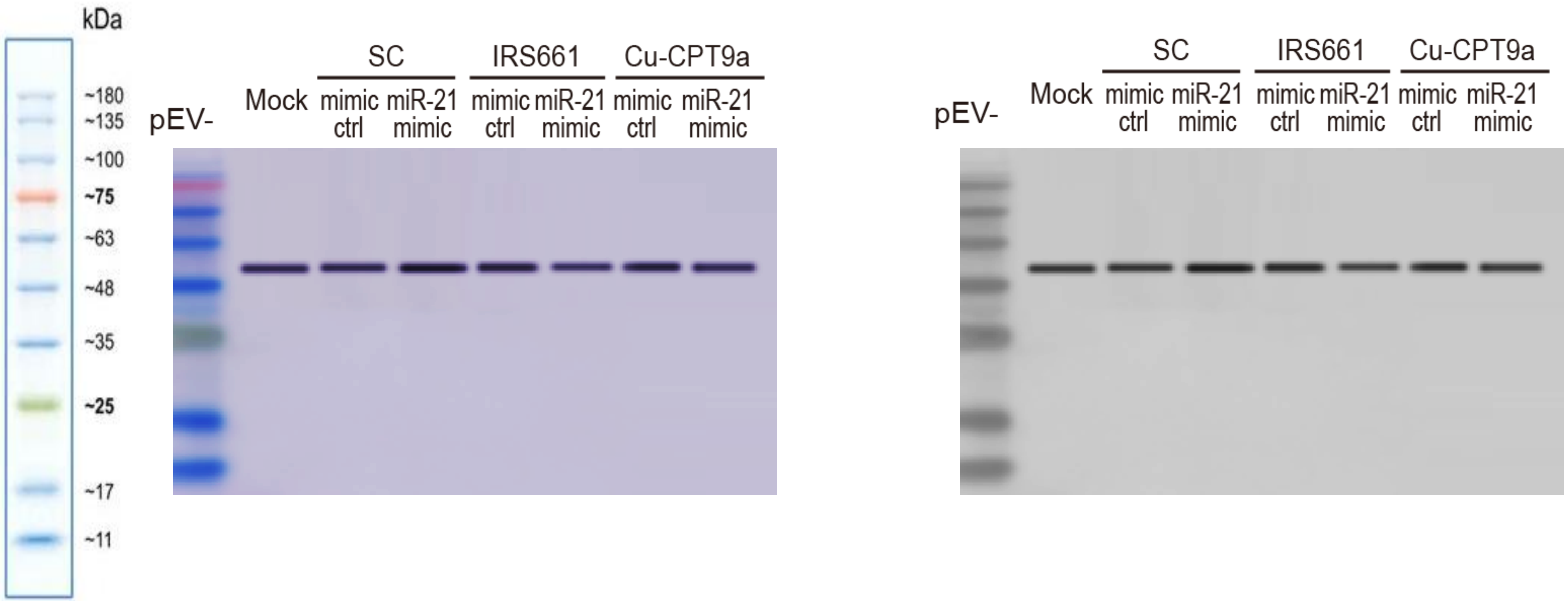

t-p38 MAPK

Fig. 5G

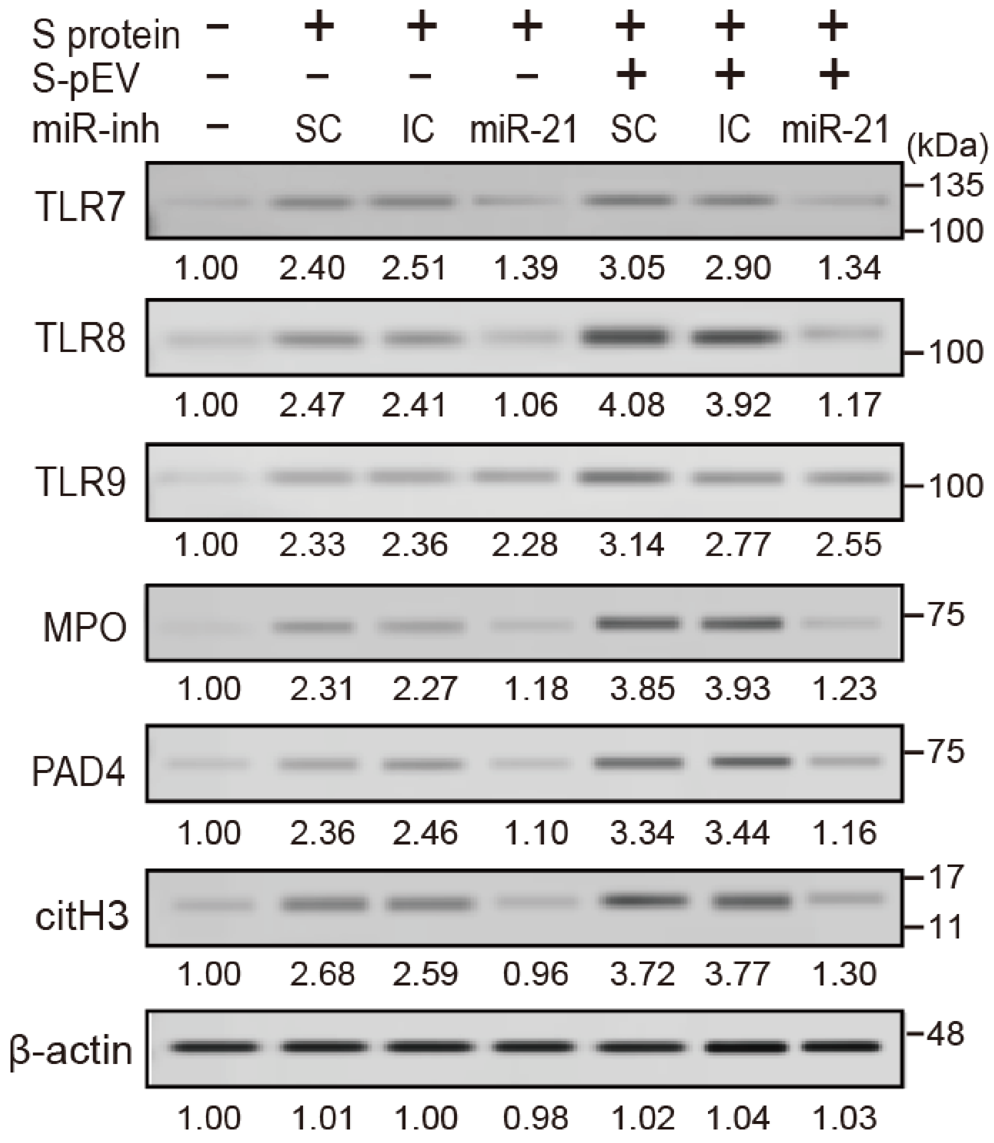
S protein − + + + + + +
S-pEV − − − − + + +
miR-inh − SC IC miR-21 SC IC miR-21
(kDa)
TLR7
135
100
1.00 2.40 2.51 1.39 3.05 2.90 1.34
TLR8
100
1.00 2.47 2.41 1.06 4.08 3.92 1.17
TLR9
100
1.00 2.33 2.36 2.28 3.14 2.77 2.55
MPO
75
1.00 2.31 2.27 1.18 3.85 3.93 1.23
PAD4
75
1.00 2.36 2.46 1.10 3.34 3.44 1.16
citH3
17
11
1.00 2.68 2.59 0.96 3.72 3.77 1.30
β-actin
48
1.00 1.01 1.00 0.98 1.02 1.04 1.03

TLR7 (121 kDa)

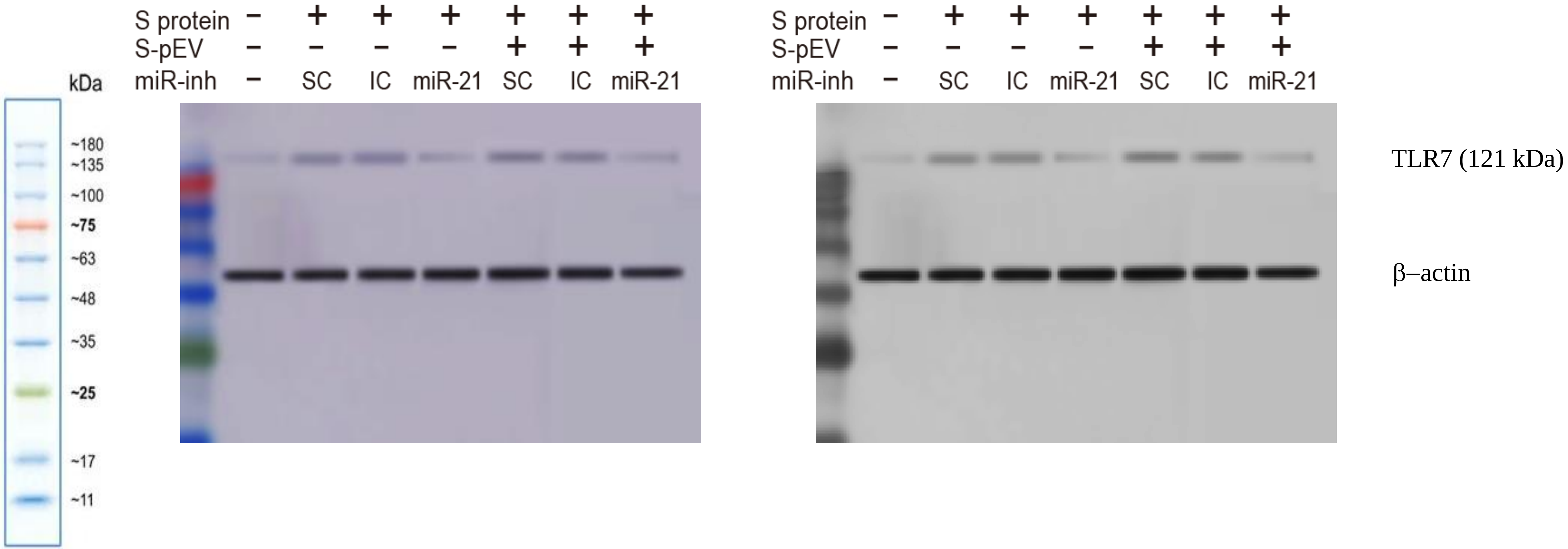

kDa
~180
~135
~100
~75
~63
~48
~35
~25
~17
~11
S protein − + + + + + +
S-pEV − − − − + + +
miR-inh − SC IC miR-21 SC IC miR-21
S protein − + + + + + +
S-pEV − − − − + + +
miR-inh − SC IC miR-21 SC IC miR-21
TLR7 (121 kDa)
β–actin

TLR8 (110 kDa)

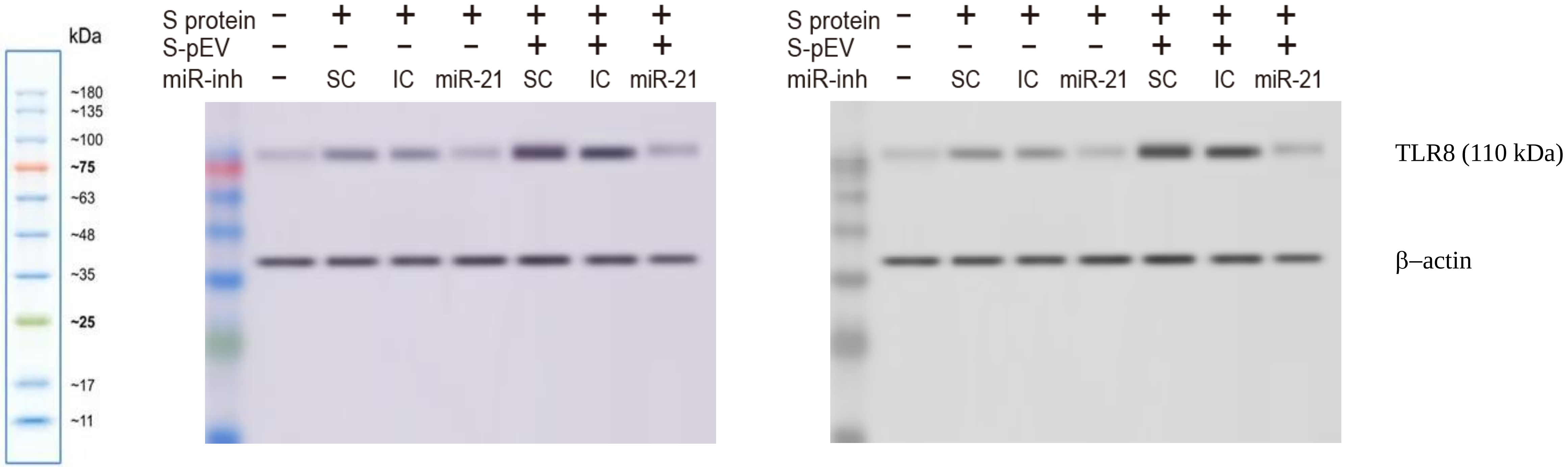
kDa
~180
~135
~100
~75
~63
~48
~35
~25
~17
~11
S protein − + + + + + +
S-pEV − − − − + + +
miR-inh − SC IC miR-21 SC IC miR-21
S protein − + + + + + +
S-pEV − − − − + + +
miR-inh − SC IC miR-21 SC IC miR-21
TLR8 (110 kDa)
β–actin

TLR9 (113 kDa)

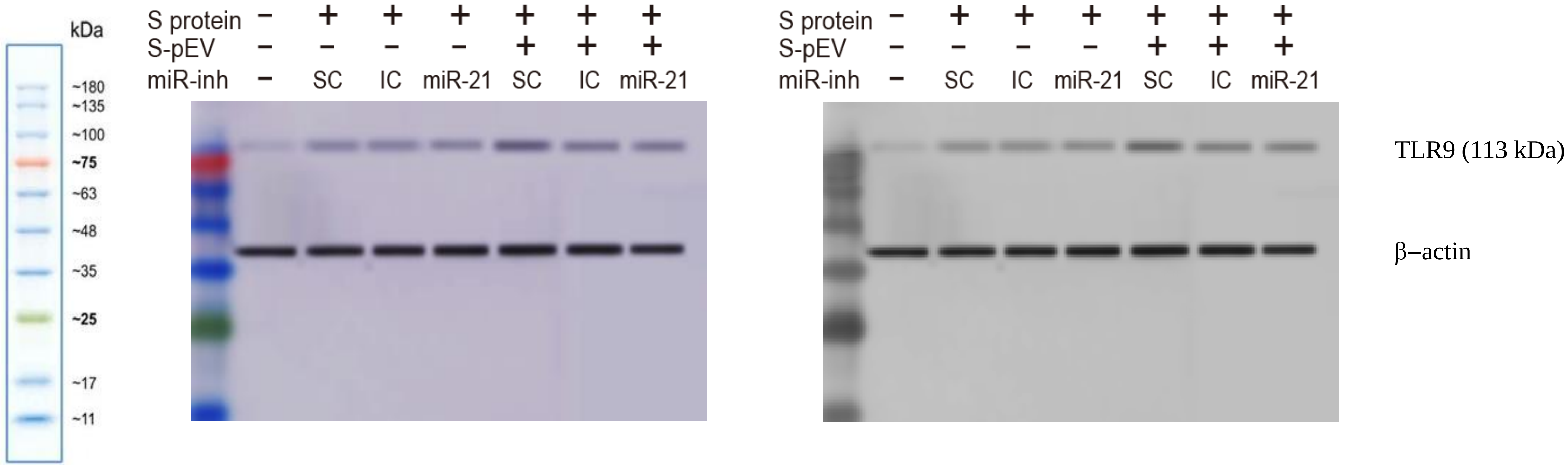
kDa
~180
~135
~100
~75
~63
~48
~35
~25
~17
~11
S protein − + + + + + +
S-pEV − − − − + + +
miR-inh − SC IC miR-21 SC IC miR-21
S protein − + + + + + +
S-pEV − − − − + + +
miR-inh − SC IC miR-21 SC IC miR-21
TLR9 (113 kDa)
β–actin

MPO (72 kDa)

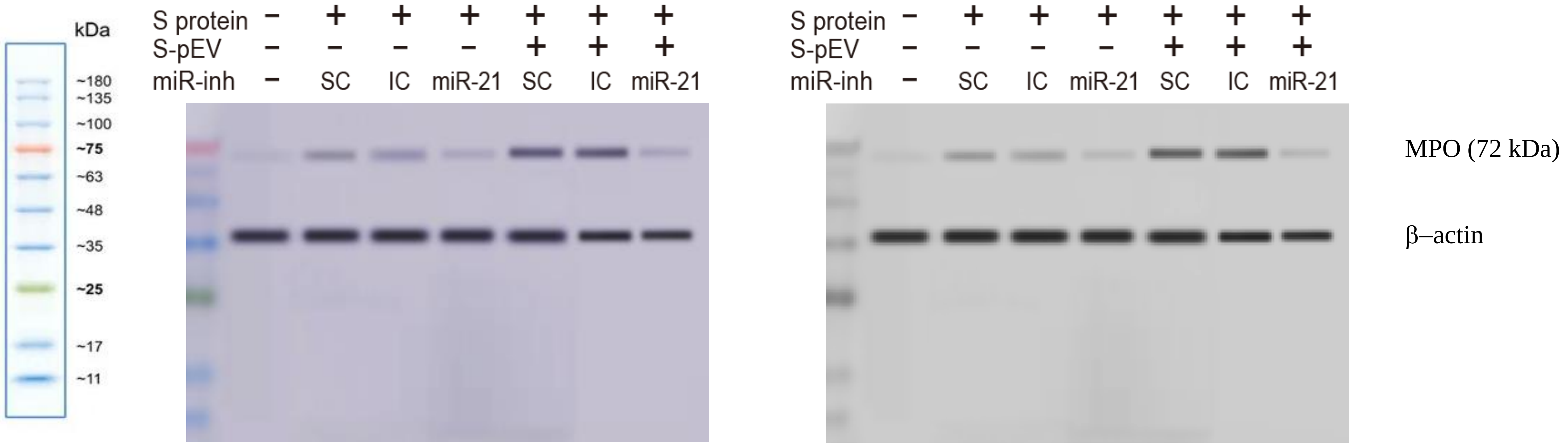

kDa
~180
~135
~100
~75
~63
~48
~35
~25
~17
~11
S protein − + + + + + +
S-pEV − − − − + + +
miR-inh − SC IC miR-21 SC IC miR-21
S protein − + + + + + +
S-pEV − − − − + + +
miR-inh − SC IC miR-21 SC IC miR-21
MPO (72 kDa)
β–actin

PAD4 (72 kDa)

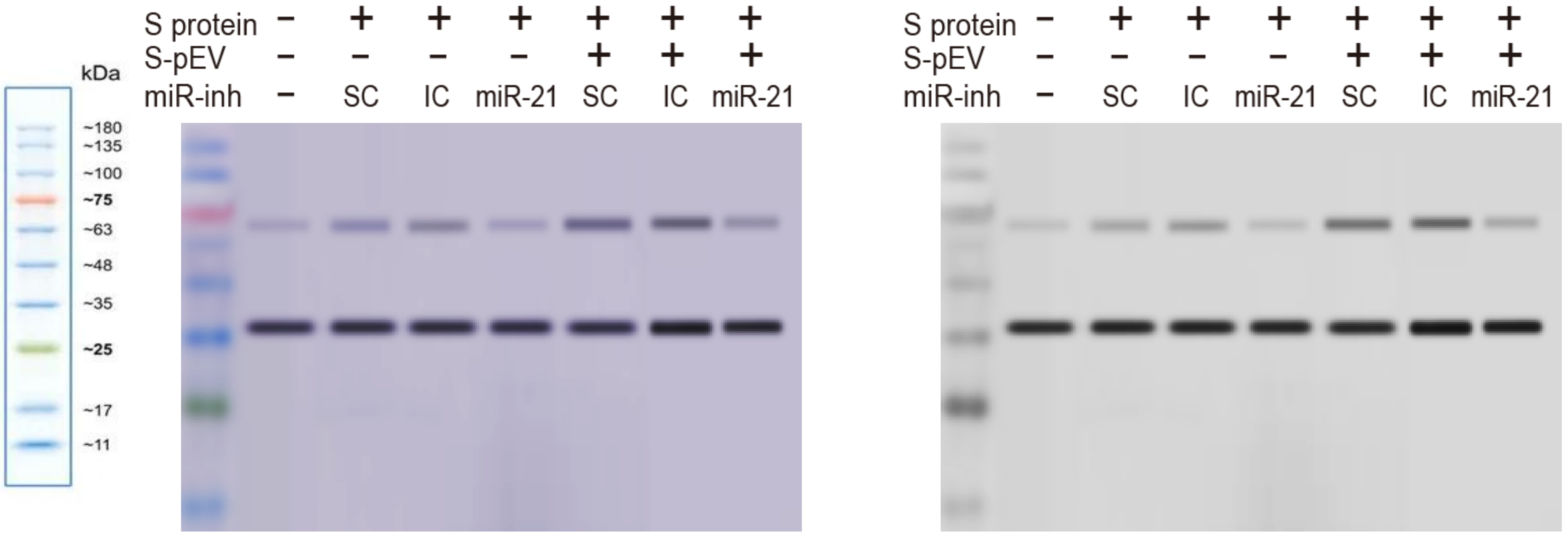

PAD4 (72 kDa)

β–actin

citH3 (14 kDa)

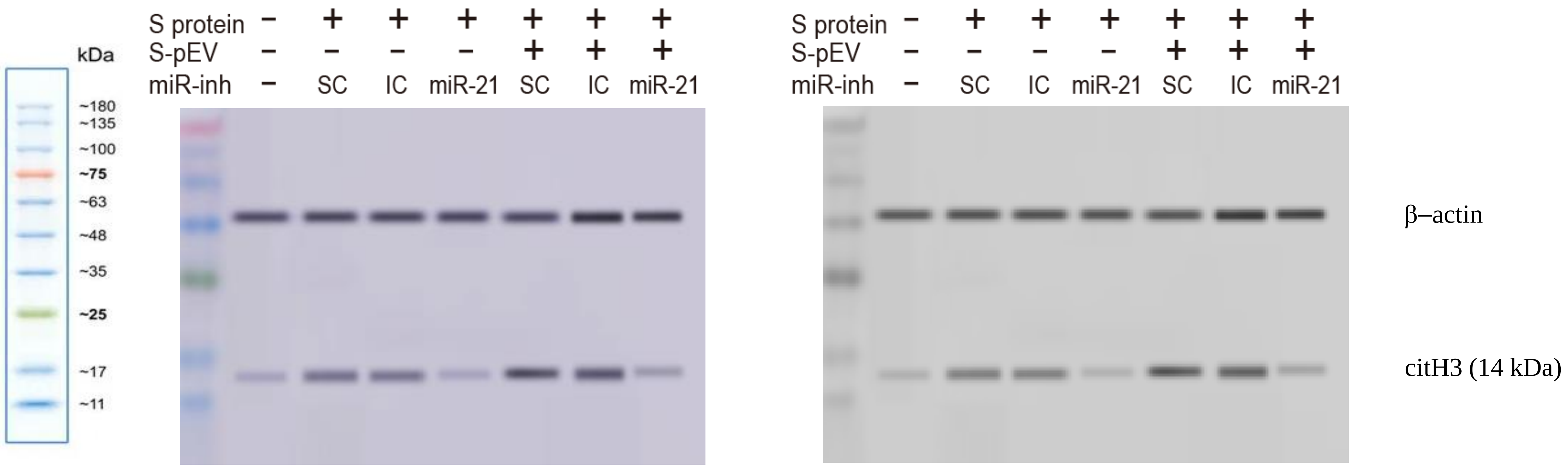
kDa
~180
~135
~100
~75
~63
~48
~35
~25
~17
~11
S protein − + + + + + +
S-pEV − − − − + + +
miR-inh − SC IC miR-21 SC IC miR-21
S protein − + + + + + +
S-pEV − − − − + + +
miR-inh − SC IC miR-21 SC IC miR-21
β–actin
citH3 (14 kDa)

Fig. 6E

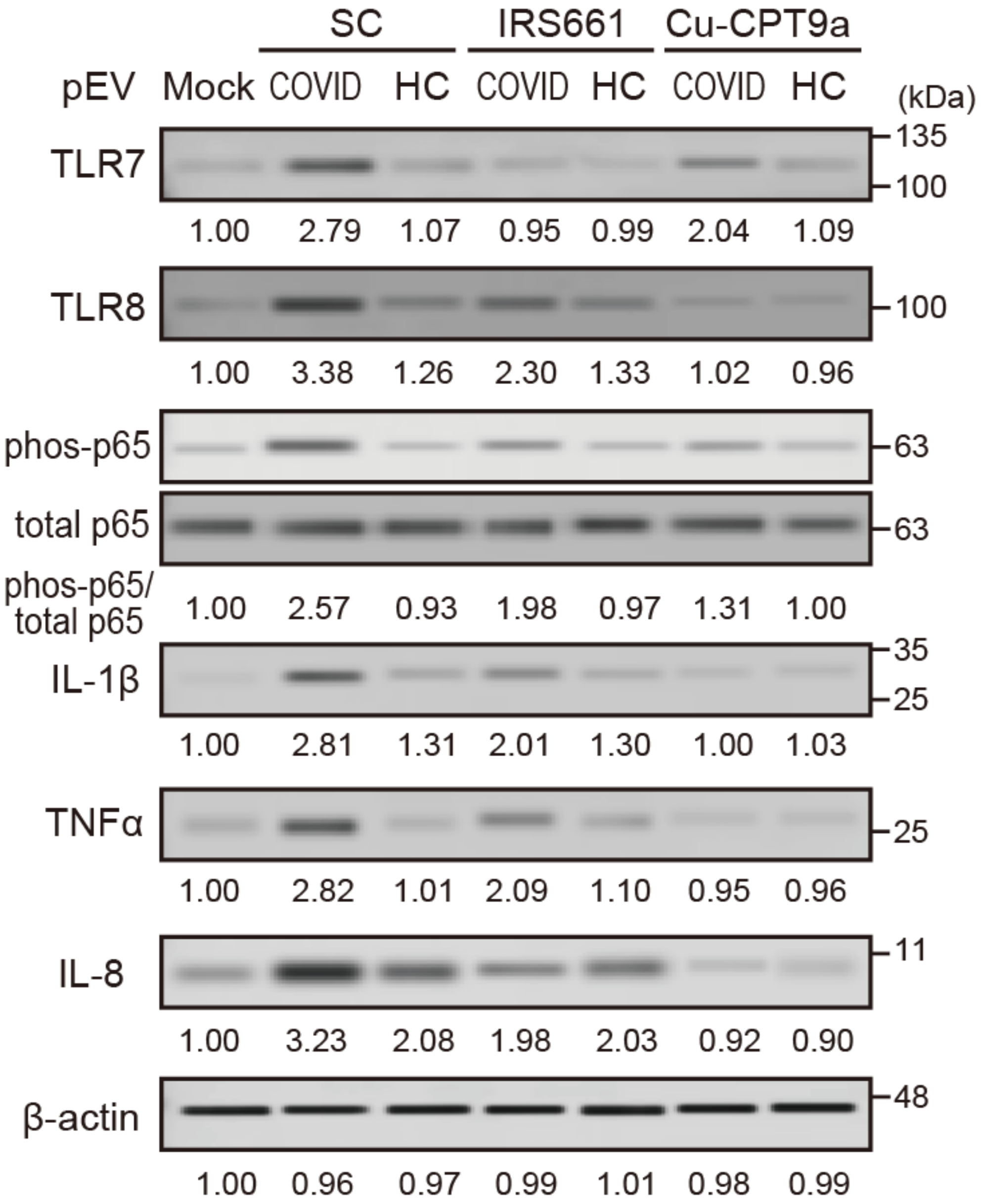

SC
IRS661
Cu-CPT9a
pEV
Mock
COVID
HC
COVID
HC
COVID
HC
(kDa)
TLR7
135
100
1.00 2.79 1.07 0.95 0.99 2.04 1.09
TLR8
100
1.00 3.38 1.26 2.30 1.33 1.02 0.96
phos-p65
63
total p65
63
phos-p65/ total p65
1.00 2.57 0.93 1.98 0.97 1.31 1.00
IL-1β
35
25
1.00 2.81 1.31 2.01 1.30 1.00 1.03
TNFα
25
1.00 2.82 1.01 2.09 1.10 0.95 0.96
IL-8
11
1.00 3.23 2.08 1.98 2.03 0.92 0.90
β-actin
48
1.00 0.96 0.97 0.99 1.01 0.98 0.99

TLR7 (121 kDa)

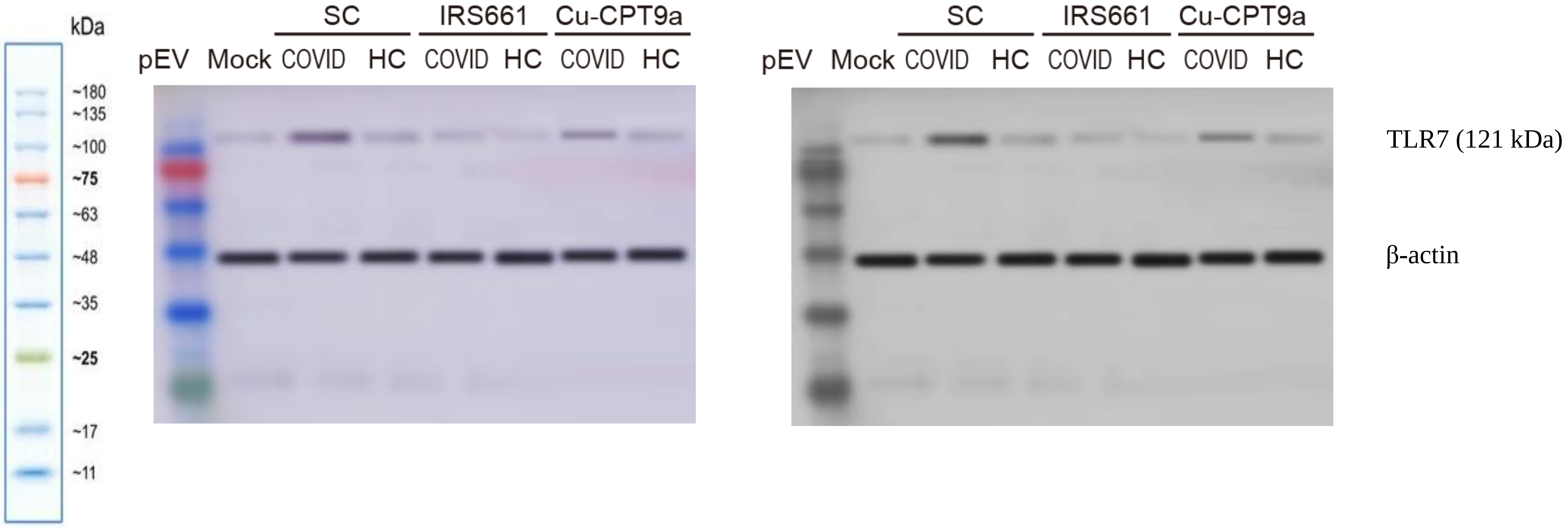
kDa
~180
~135
~100
~75
~63
~48
~35
~25
~17
~11
SC
IRS661
Cu-CPT9a
pEV
Mock
COVID
HC
COVID
HC
COVID
HC
SC
IRS661
Cu-CPT9a
pEV
Mock
COVID
HC
COVID
HC
COVID
HC
TLR7 (121 kDa)
β-actin

TLR8 (110 kDa)

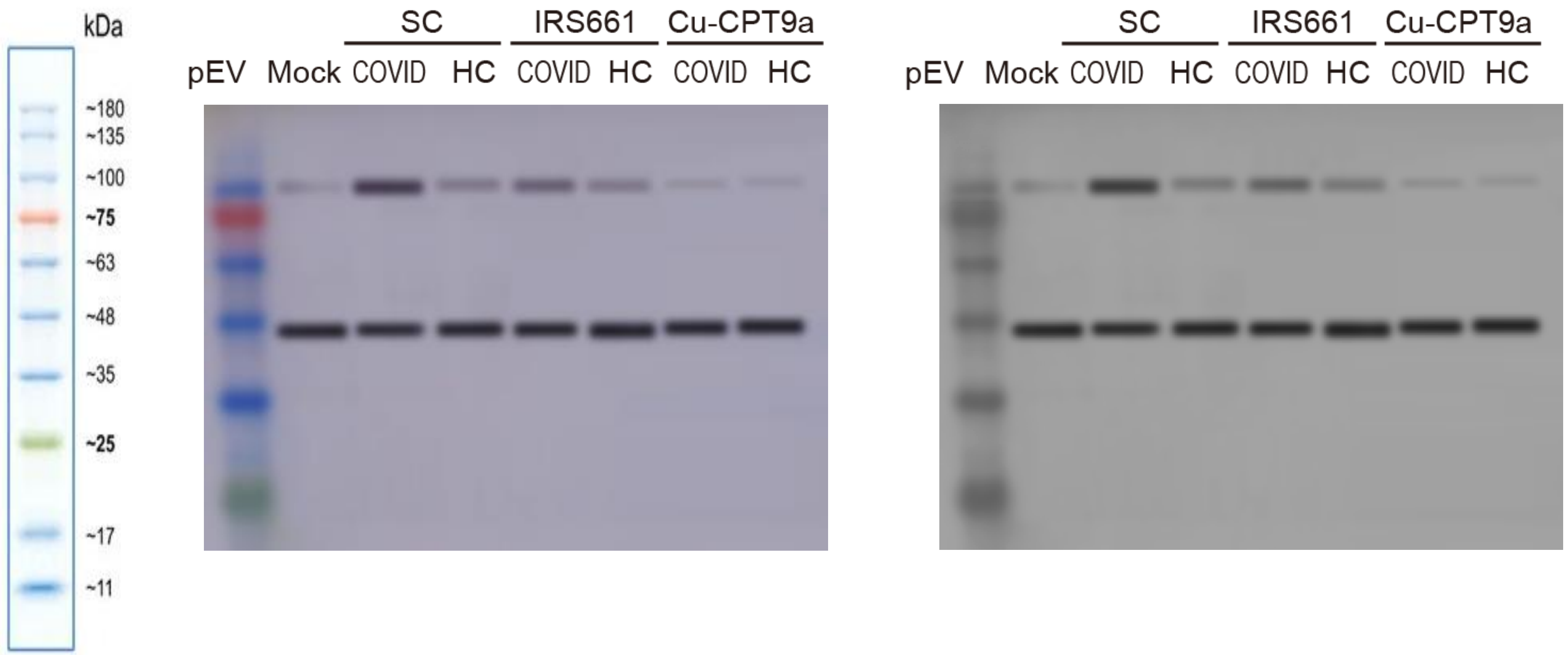

TLR8 (110 kDa)

β–actin

Phos-p65 (65 kDa)

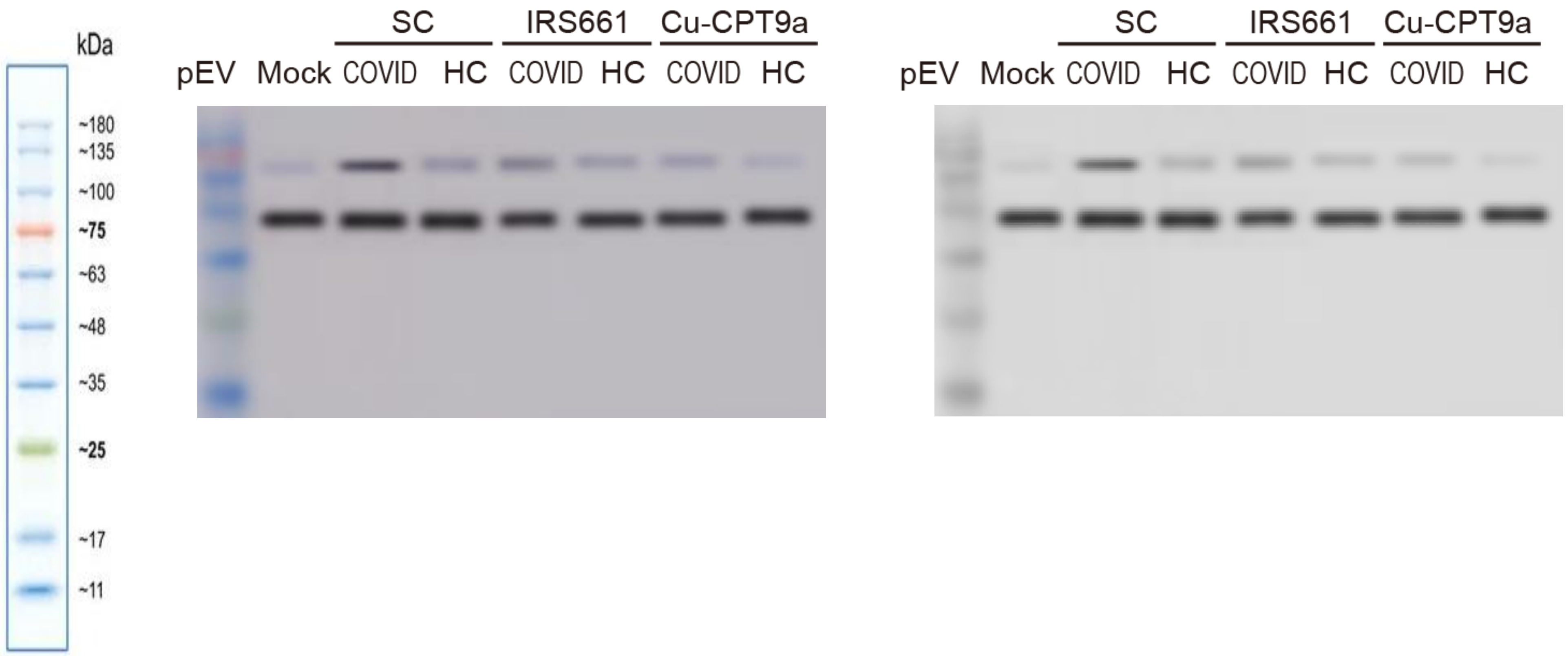

Phos-p65 (65 kDa)

β-actin

Total p65 (65 kDa)

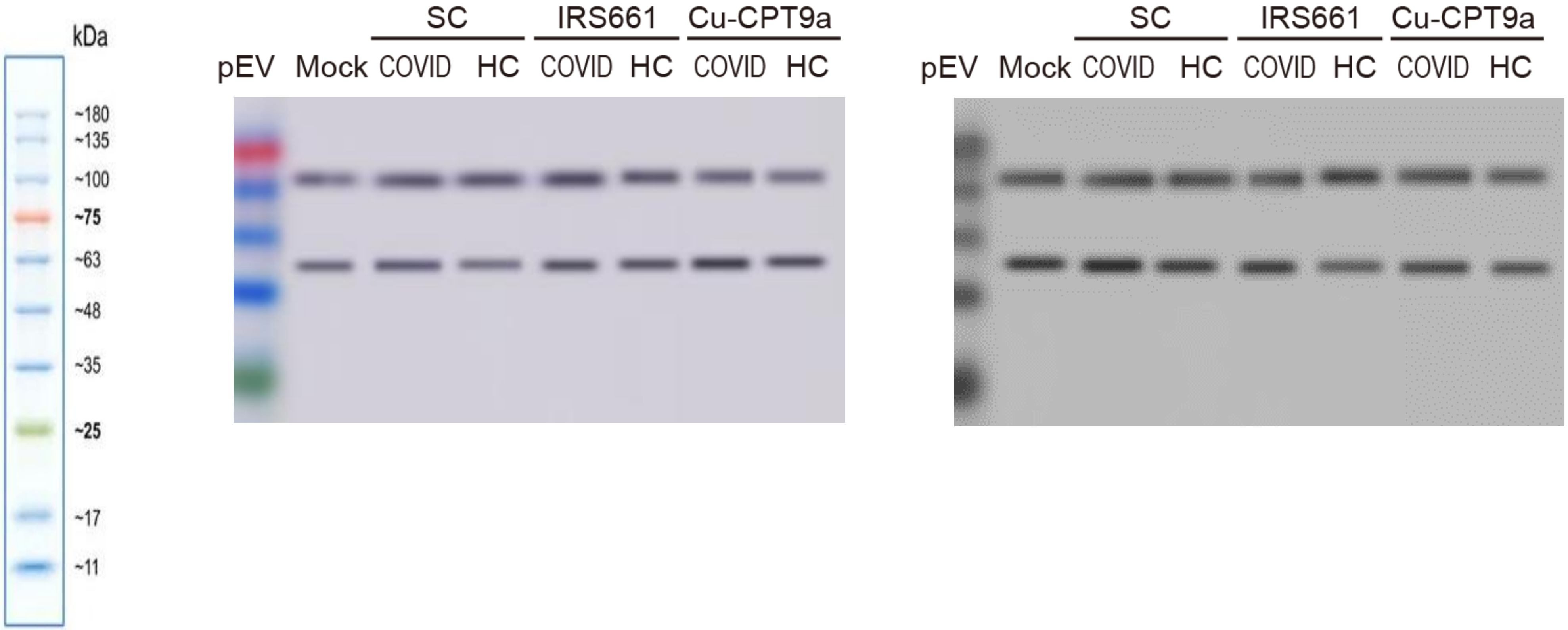

Total p65 (65 kDa)

β-actin

IL-1β (31 kDa)

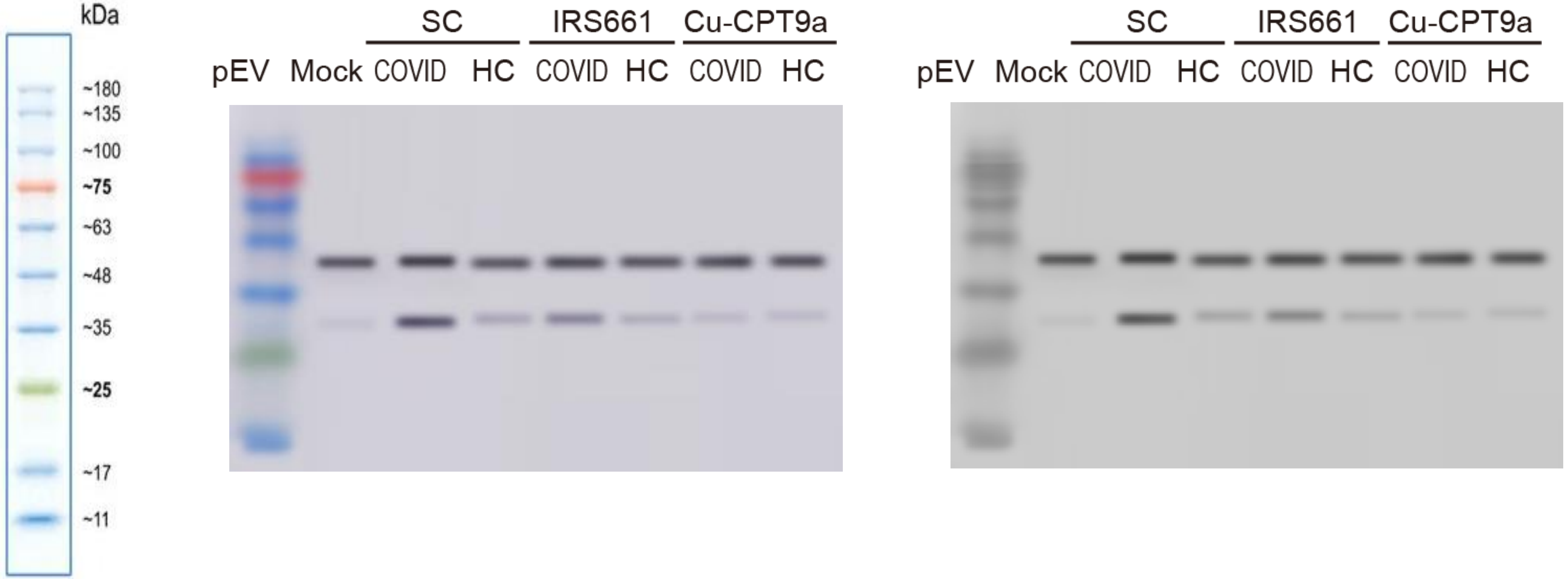

β-actin

IL-1β (31 kDa)

TNF-α (26 kDa)

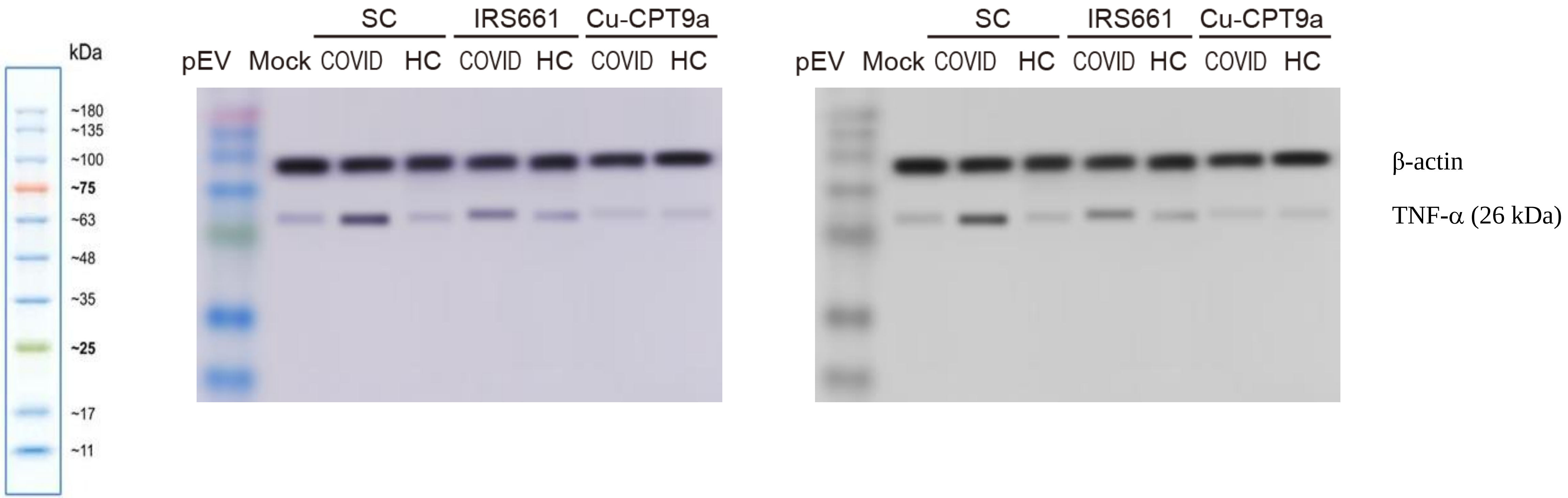
kDa
~180
~135
~100
~75
~63
~48
~35
~25
~17
~11
SC
IRS661
Cu-CPT9a
pEV
Mock
COVID
HC
COVID
HC
COVID
HC
SC
IRS661
Cu-CPT9a
pEV
Mock
COVID
HC
COVID
HC
COVID
HC
β-actin
TNF-α (26 kDa)

IL-8 (8 kDa)

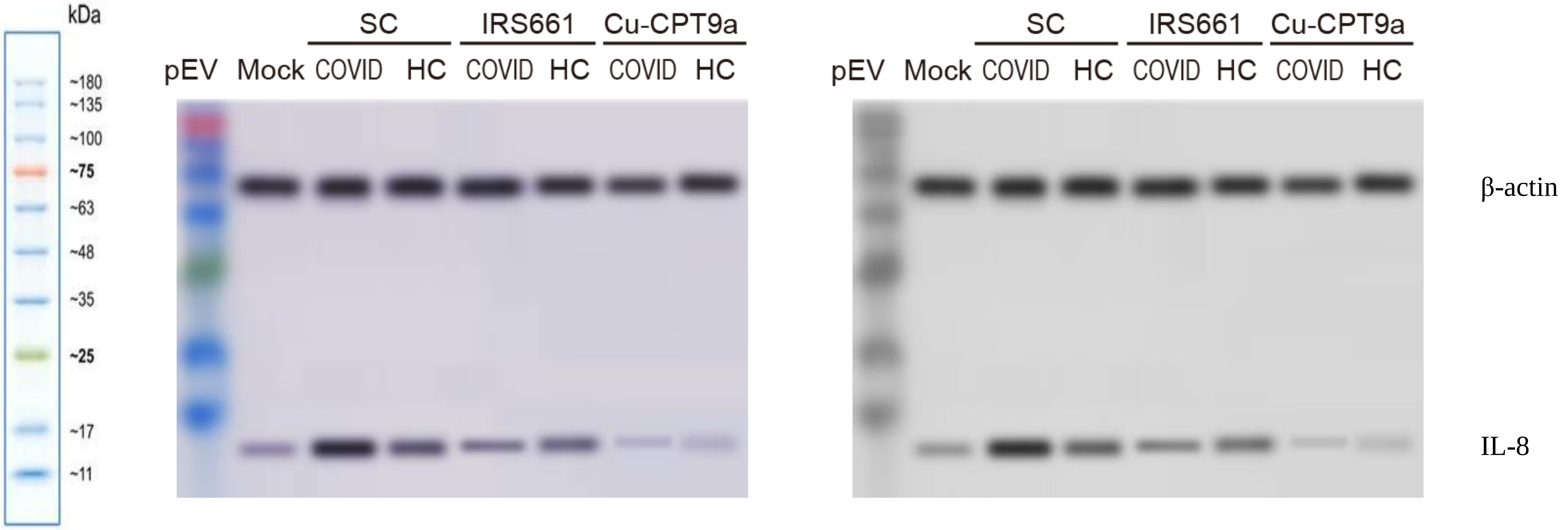
kDa
~180
~135
~100
~75
~63
~48
~35
~25
~17
~11
SC
IRS661
Cu-CPT9a
pEV
Mock
COVID
HC
COVID
HC
COVID
HC
SC
IRS661
Cu-CPT9a
pEV
Mock
COVID
HC
COVID
HC
COVID
HC
β-actin
IL-8

Fig. 6F

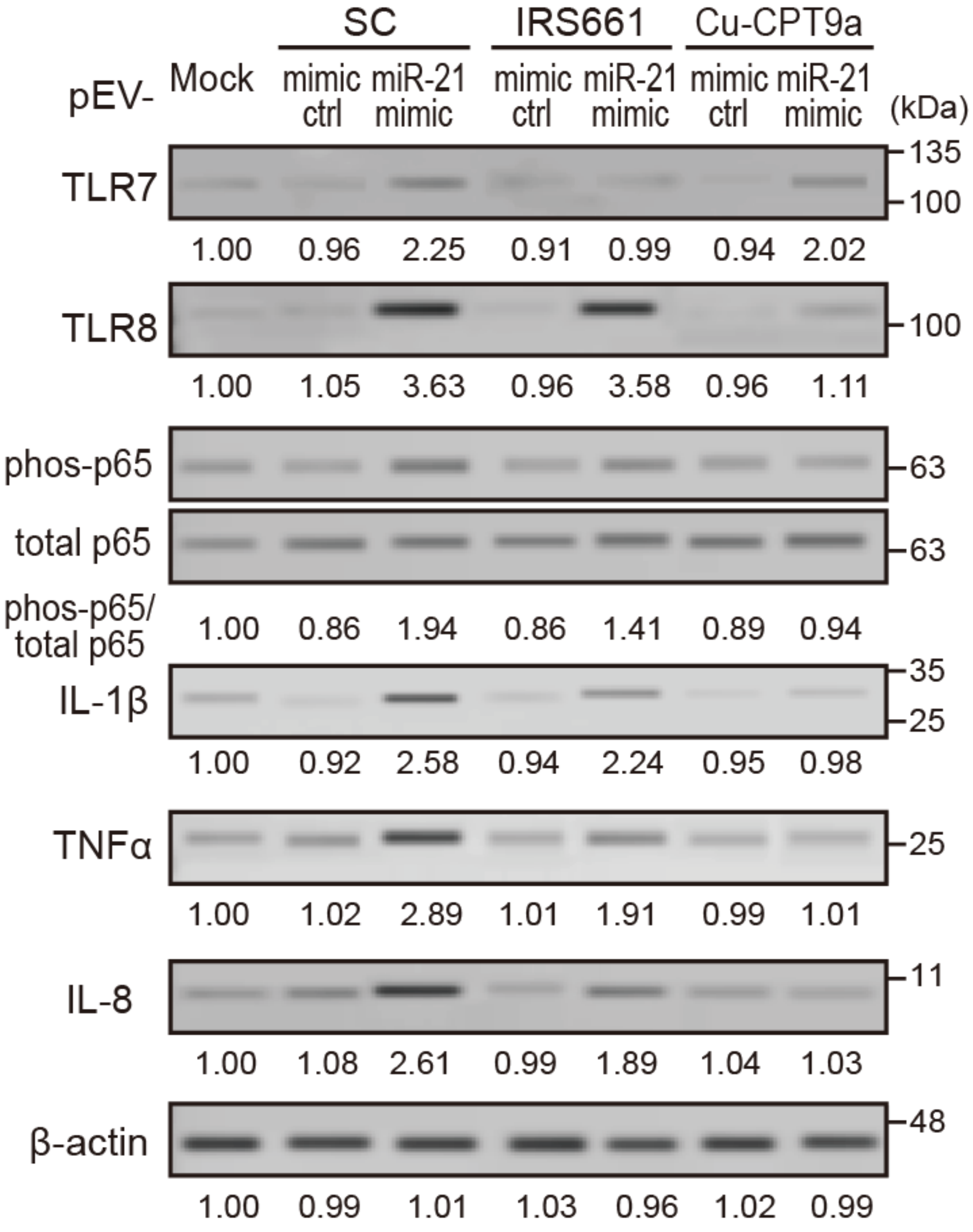
SC
IRS661
Cu-CPT9a
pEV-
Mock
mimic ctrl
miR-21 mimic
mimic ctrl
miR-21 mimic
mimic ctrl
miR-21 mimic
(kDa)
TLR7
135
100
1.00 0.96 2.25 0.91 0.99 0.94 2.02
TLR8
100
1.00 1.05 3.63 0.96 3.58 0.96 1.11
phos-p65
63
total p65
63
phos-p65/ total p65
1.00 0.86 1.94 0.86 1.41 0.89 0.94
IL-1β
35
25
1.00 0.92 2.58 0.94 2.24 0.95 0.98
TNFα
25
1.00 1.02 2.89 1.01 1.91 0.99 1.01
IL-8
11
1.00 1.08 2.61 0.99 1.89 1.04 1.03
β-actin
48
1.00 0.99 1.01 1.03 0.96 1.02 0.99

TLR7 (121 kDa)

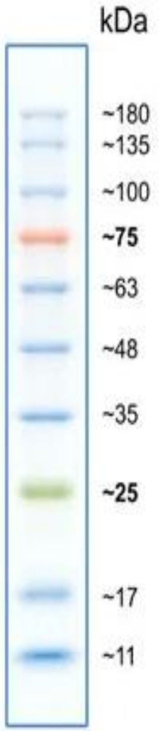

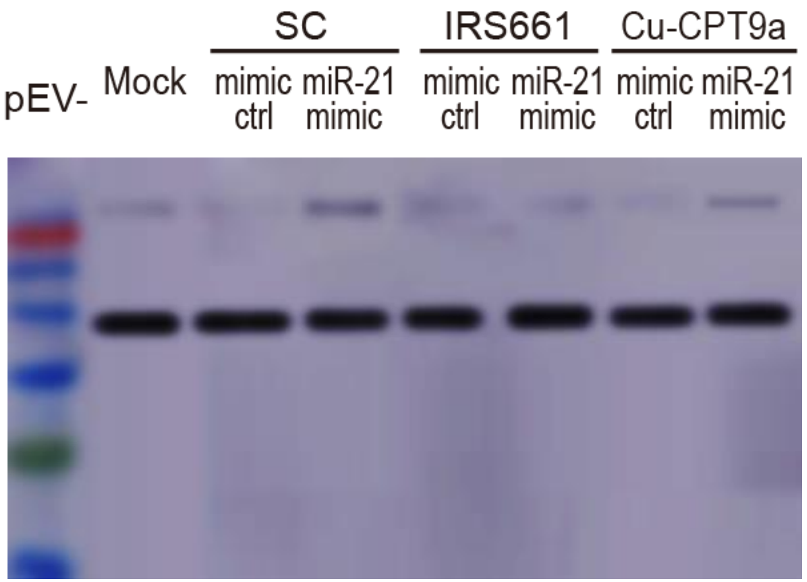

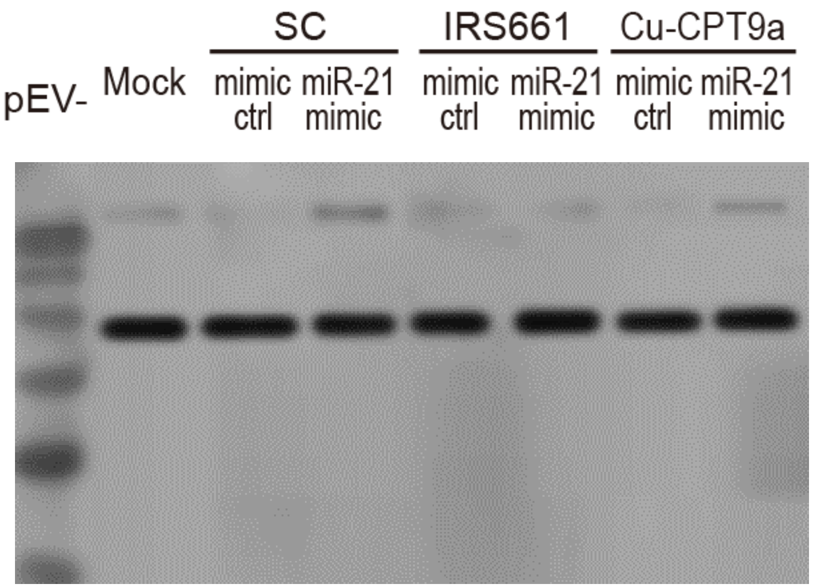

TLR7 (121 kDa)

β–actin

TLR8 (110 kDa)

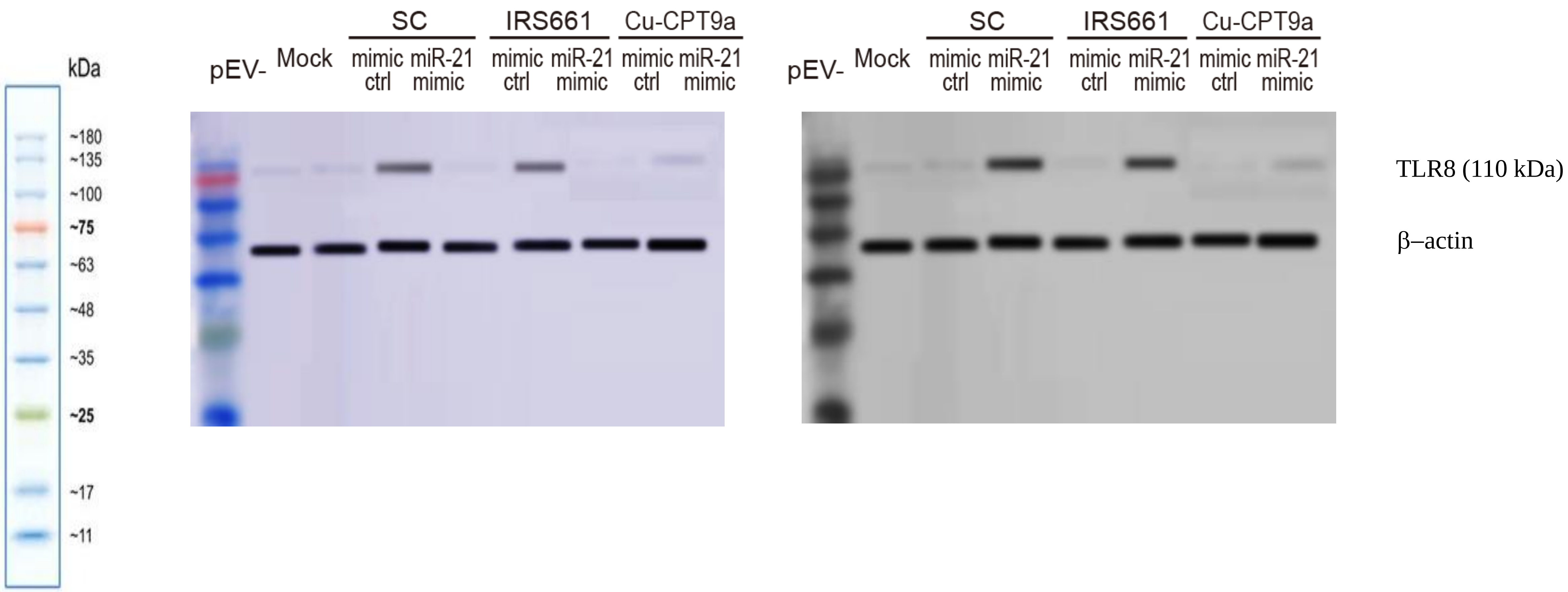
kDa
~180
~135
~100
~75
~63
~48
~35
~25
~17
~11
pEV-
Mock
SC
IRS661
Cu-CPT9a
mimic ctrl
miR-21 mimic
mimic ctrl
miR-21 mimic
mimic ctrl
miR-21 mimic
pEV-
Mock
SC
IRS661
Cu-CPT9a
mimic ctrl
miR-21 mimic
mimic ctrl
miR-21 mimic
mimic ctrl
miR-21 mimic
TLR8 (110 kDa)
β–actin

Phos-p65 (65 kDa)

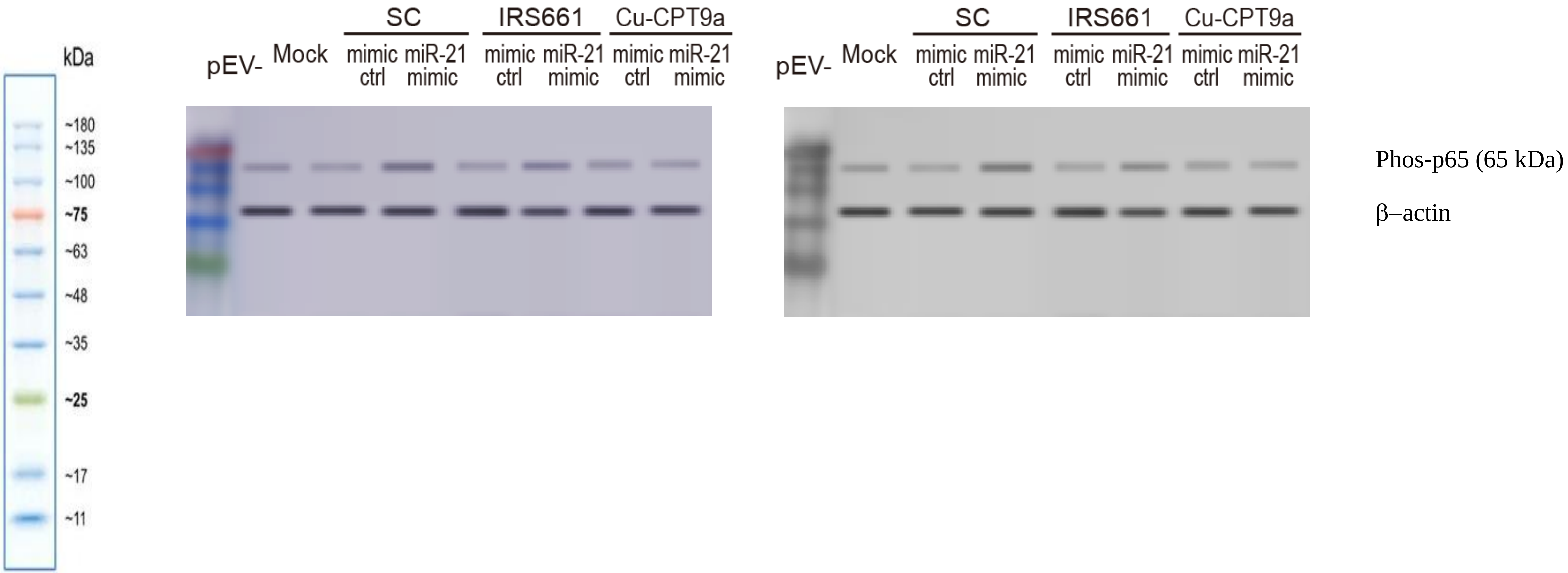

Total-p65 (65 kDa)

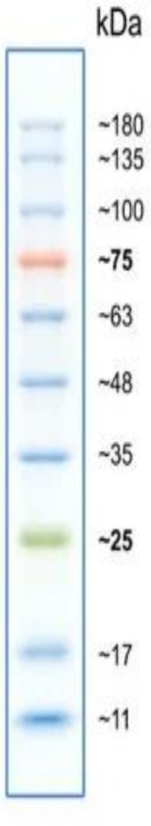

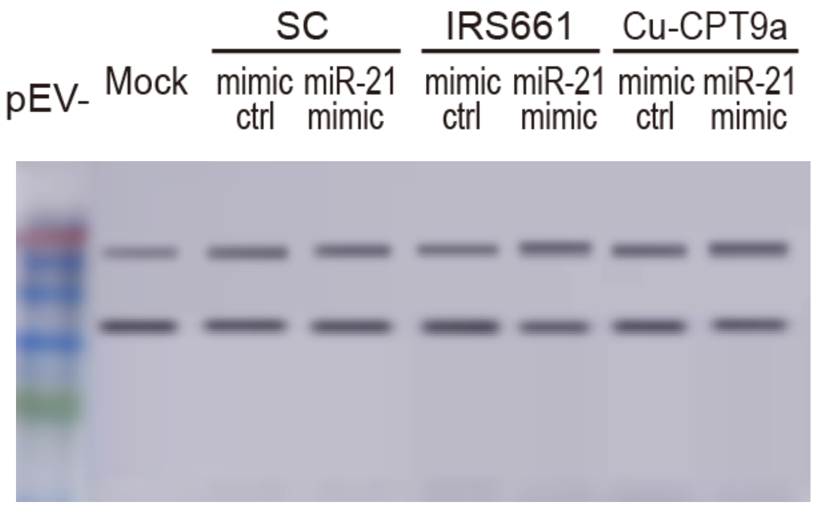

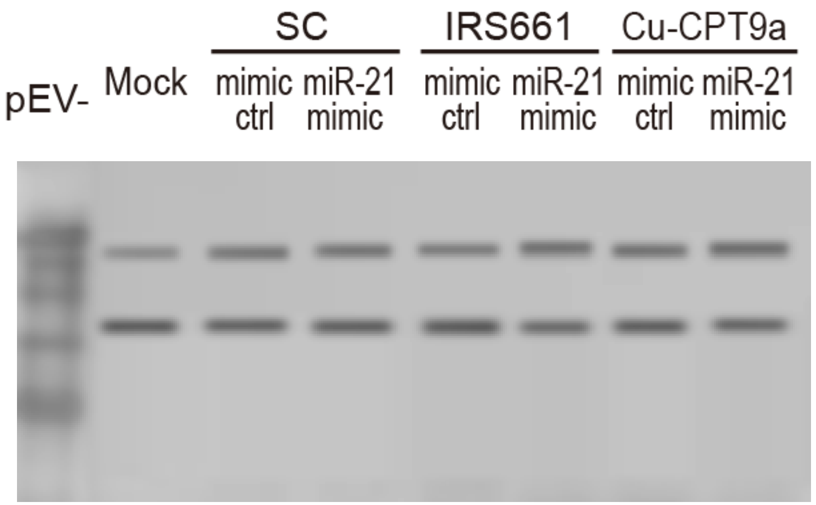

Total-p65 (65 kDa)

β-actin (45 kDa)

IL-1β (31 kDa)

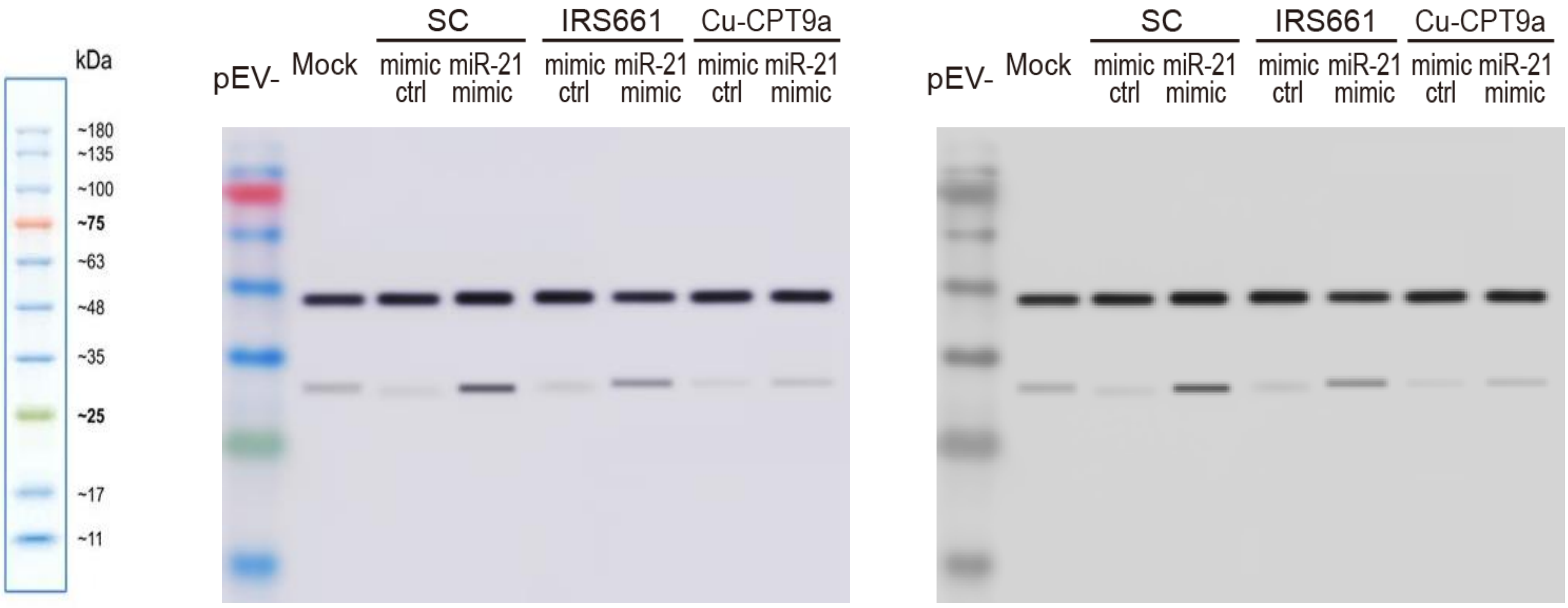

β-actin

IL-1β (31 kDa)

TNF-α (26 kDa)

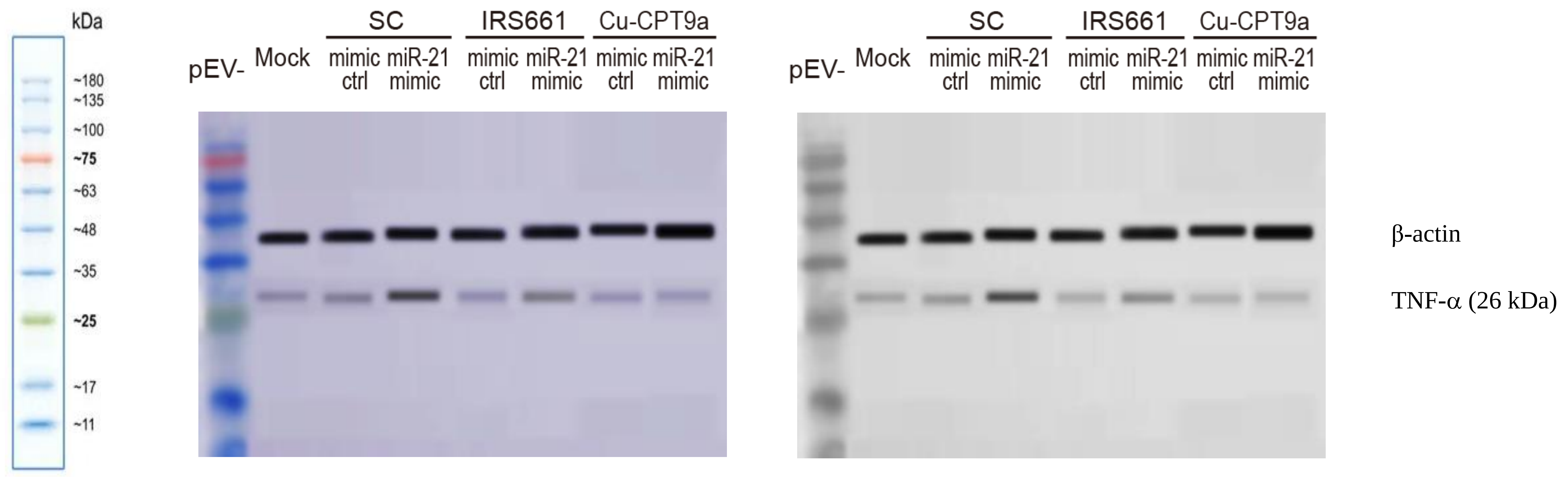

kDa
~180
~135
~100
~75
~63
~48
~35
~25
~17
~11
pEV-
Mock
SC
IRS661
Cu-CPT9a
mimic ctrl
miR-21 mimic
mimic ctrl
miR-21 mimic
mimic ctrl
miR-21 mimic
pEV-
Mock
SC
IRS661
Cu-CPT9a
mimic ctrl
miR-21 mimic
mimic ctrl
miR-21 mimic
mimic ctrl
miR-21 mimic
β-actin
TNF-α (26 kDa)

IL-8 (8 kDa)

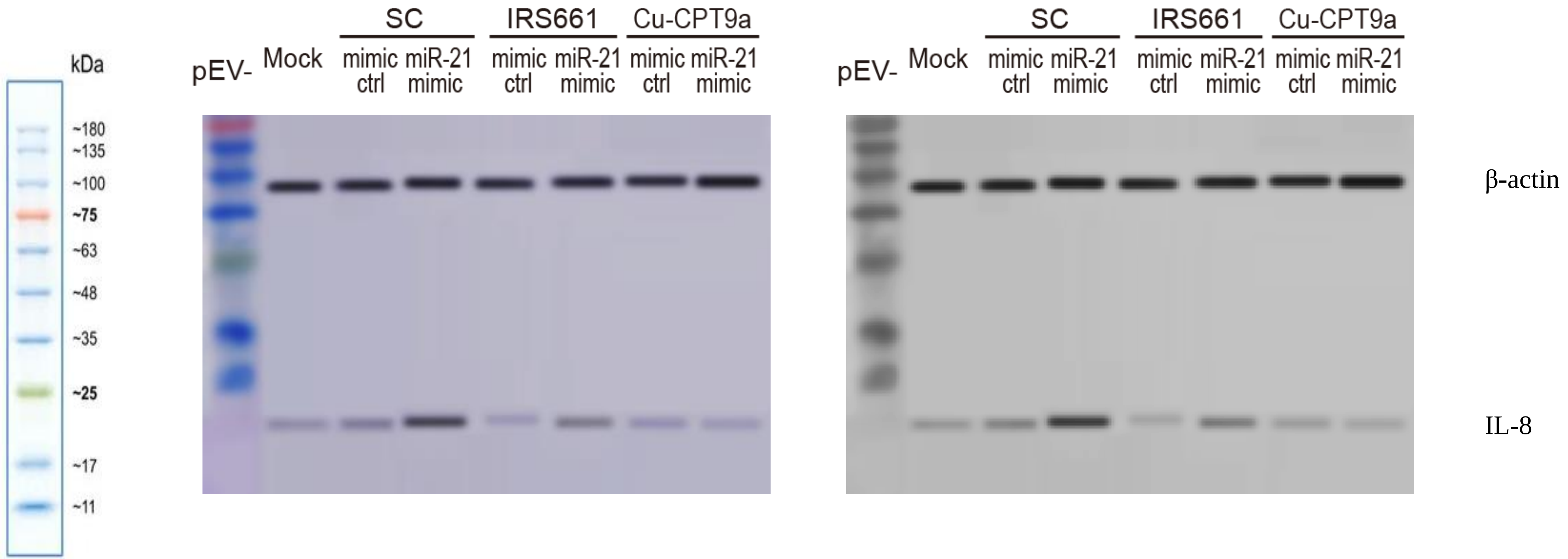
kDa
~180
~135
~100
~75
~63
~48
~35
~25
~17
~11
SC
IRS661
Cu-CPT9a
pEV-
Mock
mimic ctrl
miR-21 mimic
mimic ctrl
miR-21 mimic
mimic ctrl
miR-21 mimic
SC
IRS661
Cu-CPT9a
pEV-
Mock
mimic ctrl
miR-21 mimic
mimic ctrl
miR-21 mimic
mimic ctrl
miR-21 mimic
β-actin
IL-8

Fig. S3A

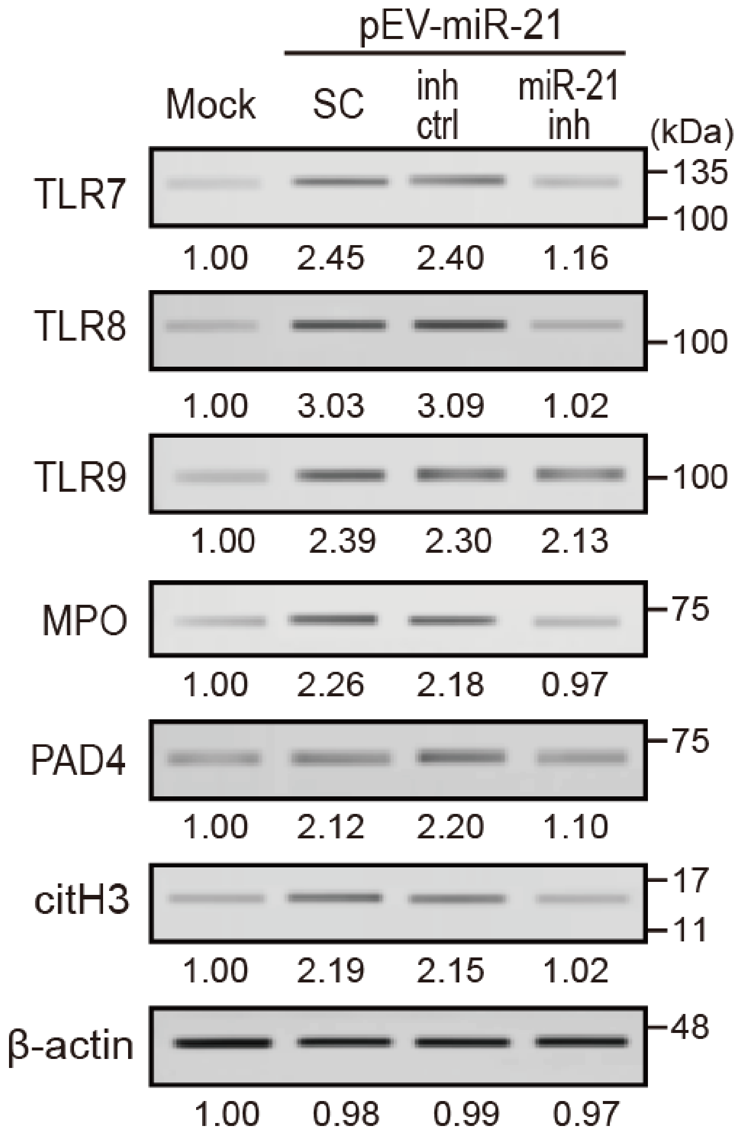

pEV-miR-21
Mock
SC
inh ctrl
miR-21 inh
(kDa)
TLR7
135
100
1.00 2.45 2.40 1.16
TLR8
100
1.00 3.03 3.09 1.02
TLR9
100
1.00 2.39 2.30 2.13
MPO
75
1.00 2.26 2.18 0.97
PAD4
75
1.00 2.12 2.20 1.10
citH3
17
11
1.00 2.19 2.15 1.02
β-actin
48
1.00 0.98 0.99 0.97

TLR7 (121 kDa)

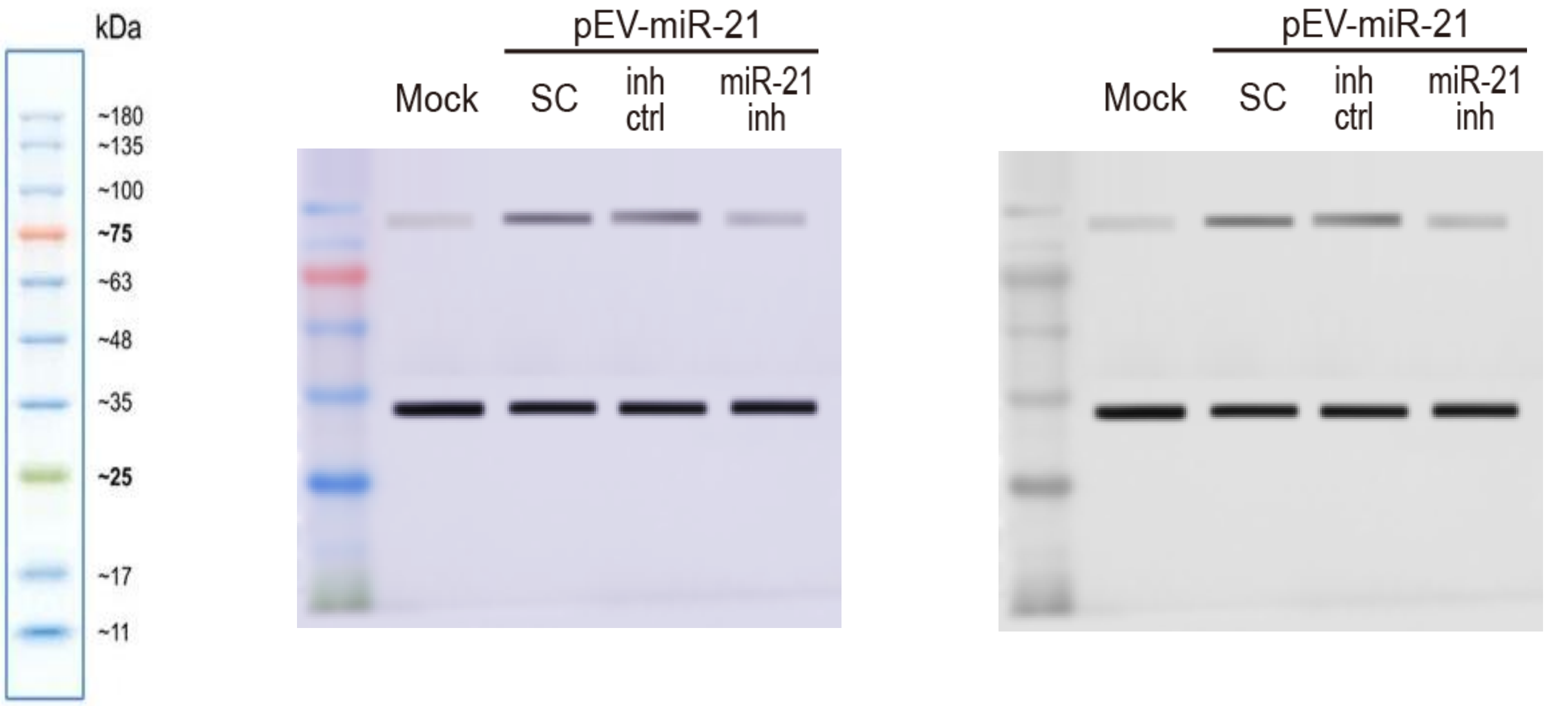

TLR7 (121 kDa)

β–actin

TLR8 (110 kDa)

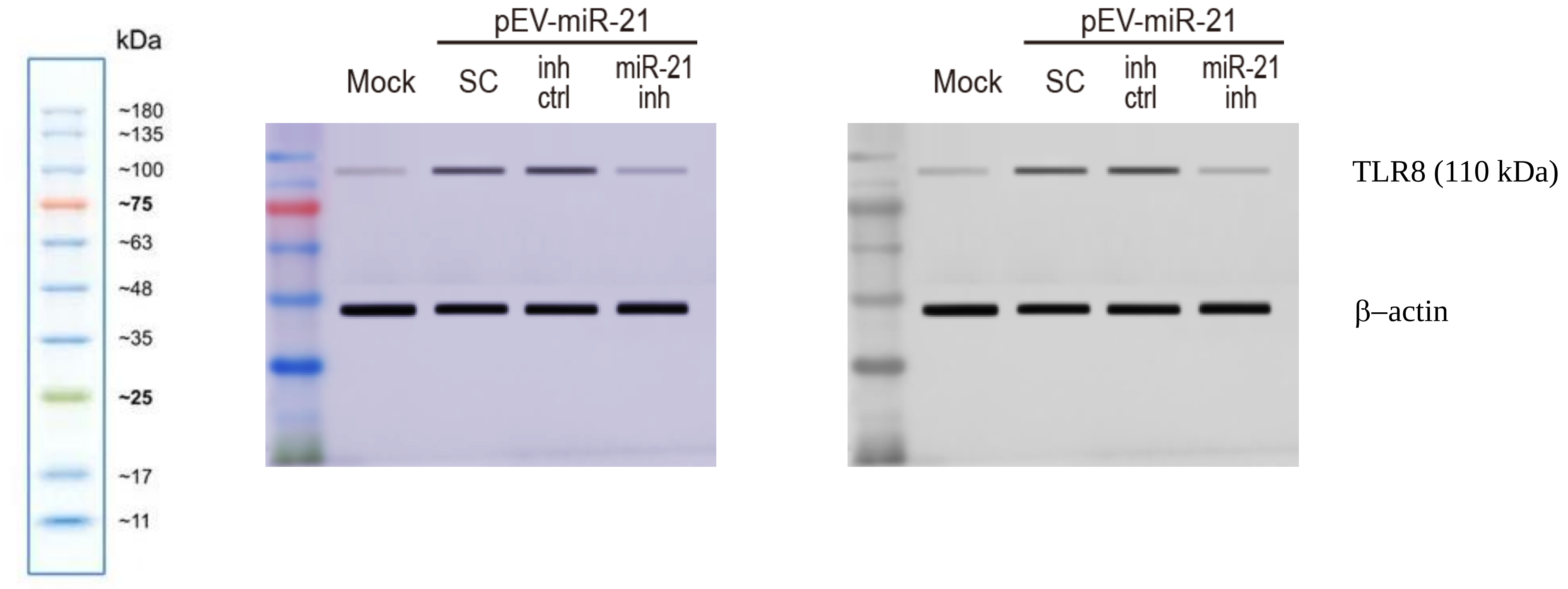
kDa
~180
~135
~100
~75
~63
~48
~35
~25
~17
~11
pEV-miR-21
Mock
SC
inh ctrl
miR-21 inh
pEV-miR-21
Mock
SC
inh ctrl
miR-21 inh
TLR8 (110 kDa)
β–actin

TLR9 (113 kDa)

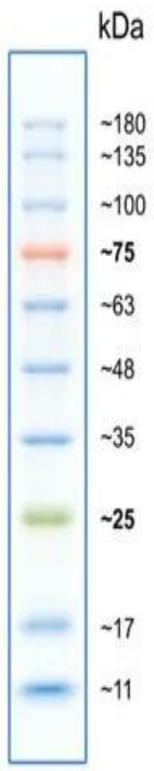

kDa
~180
~135
~100
~75
~63
~48
~35
~25
~17
~11

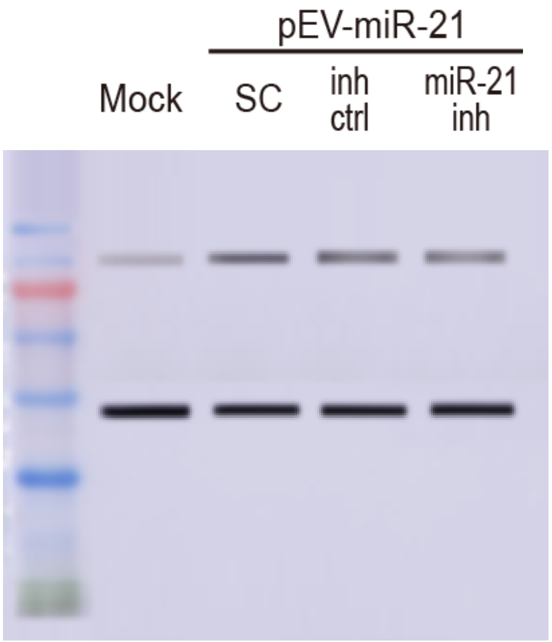

pEV-miR-21
Mock
SC
inh ctrl
miR-21 inh

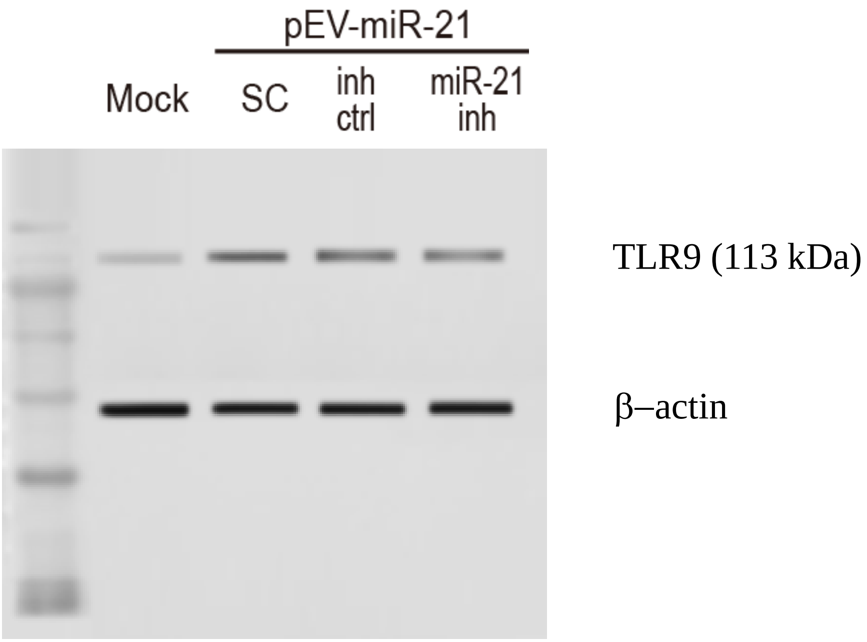

pEV-miR-21
Mock
SC
inh ctrl
miR-21 inh
TLR9 (113 kDa)
β–actin

MPO (72 kDa)

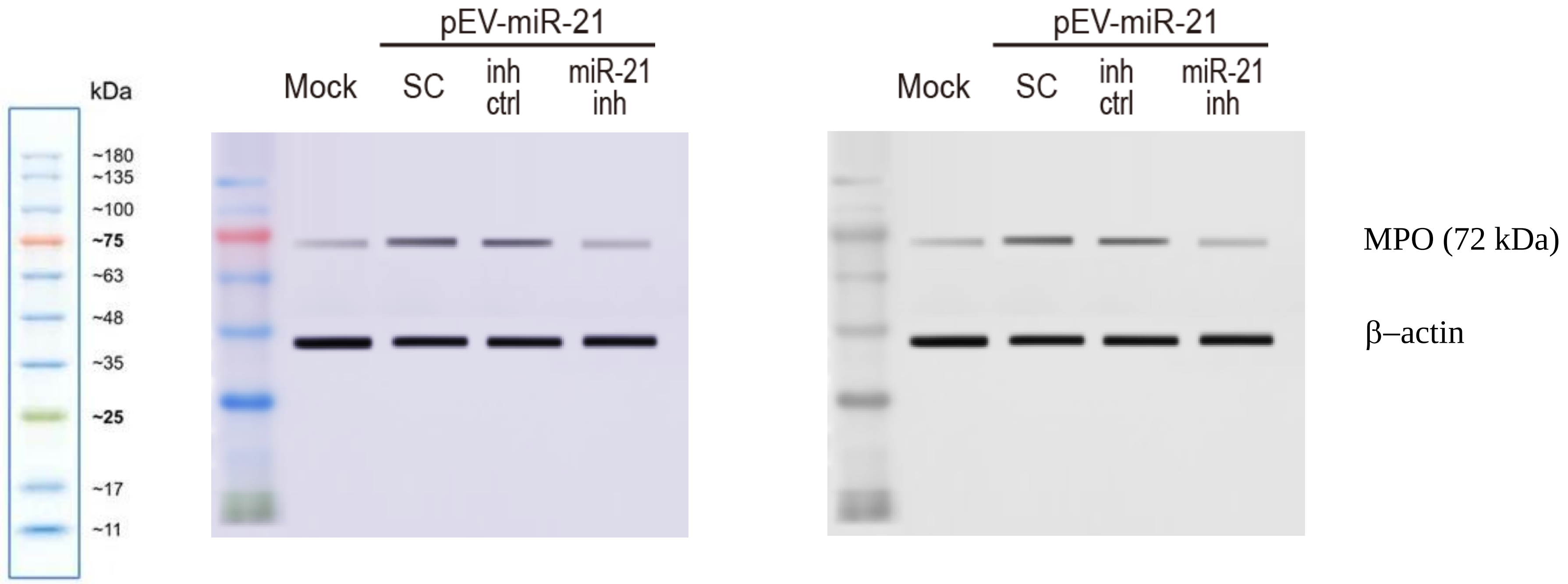
kDa
~180
~135
~100
~75
~63
~48
~35
~25
~17
~11
pEV-miR-21
Mock
SC
inh
ctrl
miR-21
inh
pEV-miR-21
Mock
SC
inh
ctrl
miR-21
inh
MPO (72 kDa)
β–actin

PAD4 (72 kDa)

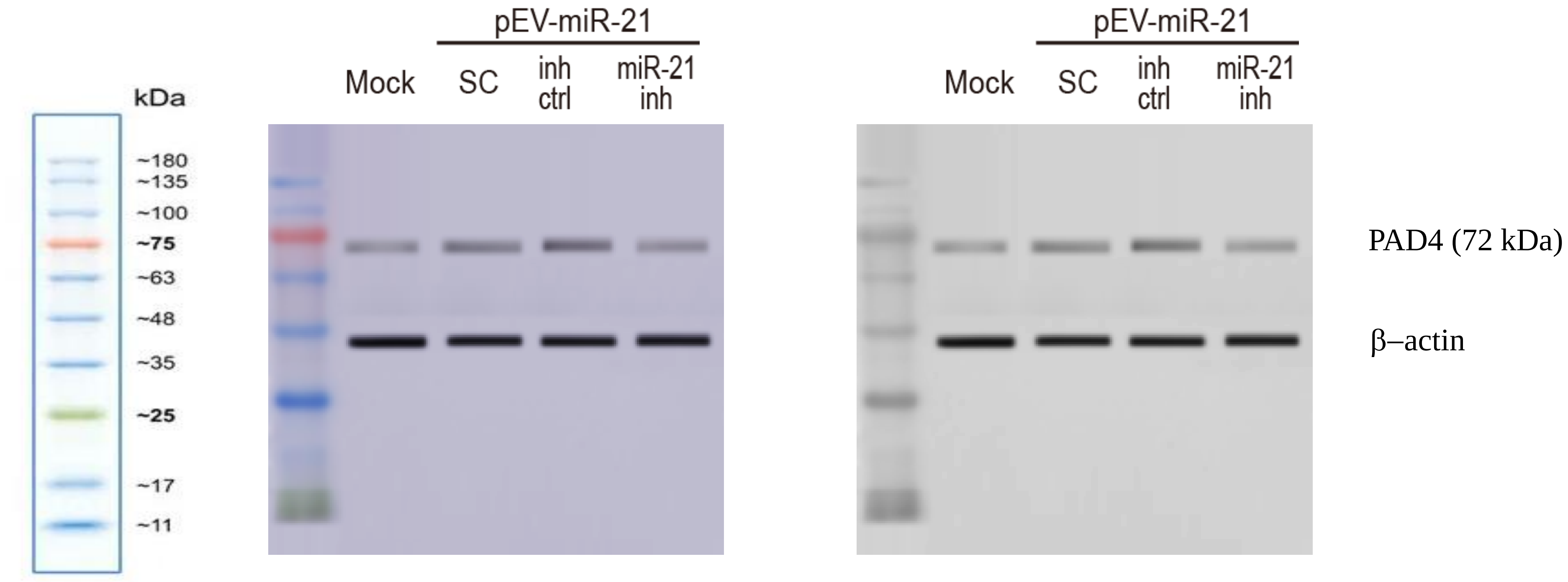
kDa
~180
~135
~100
~75
~63
~48
~35
~25
~17
~11
pEV-miR-21
Mock
SC
inh ctrl
miR-21 inh
pEV-miR-21
Mock
SC
inh ctrl
miR-21 inh
PAD4 (72 kDa)
β–actin

ciH3 (14 kDa)

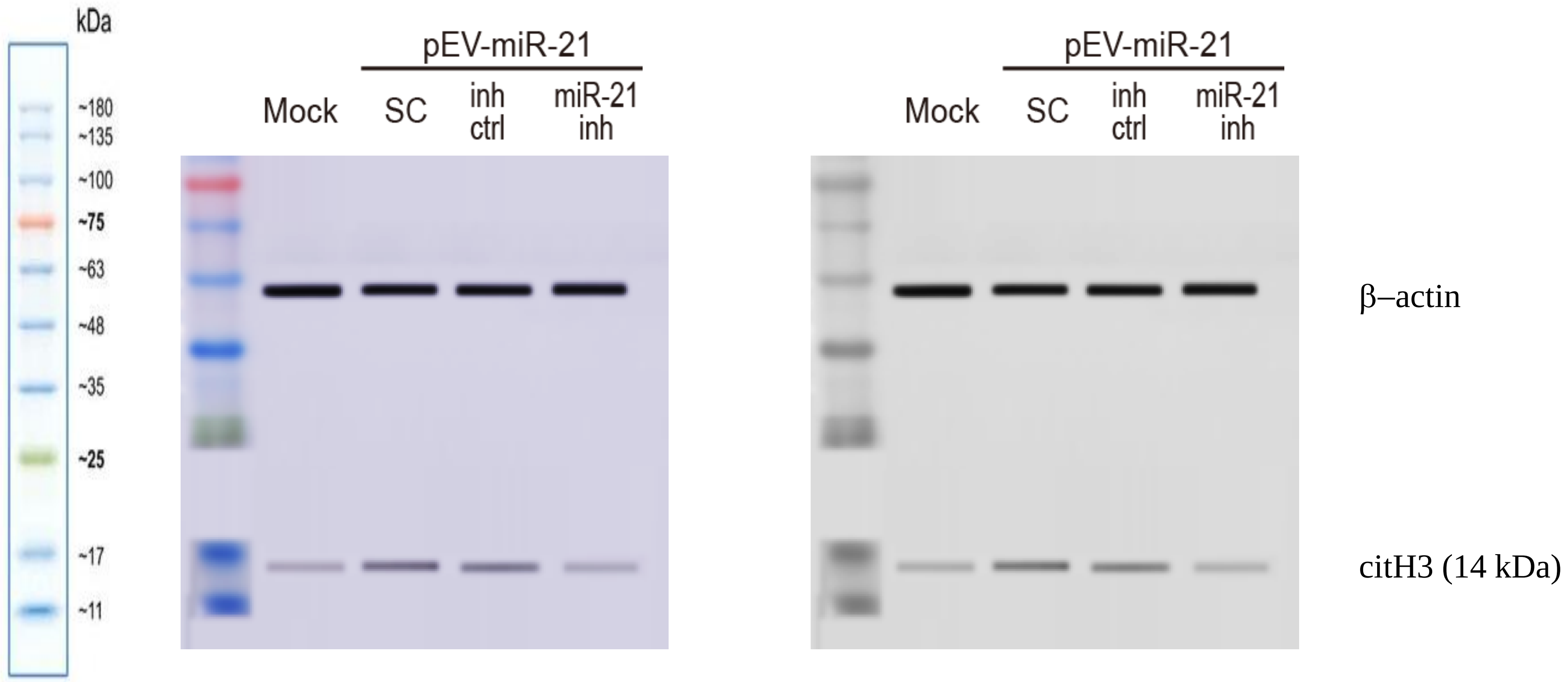

kDa
~180
~135
~100
~75
~63
~48
~35
~25
~17
~11
pEV-miR-21
Mock
SC
inh ctrl
miR-21 inh
pEV-miR-21
Mock
SC
inh ctrl
miR-21 inh
β–actin
citH3 (14 kDa)

Fig. S5A

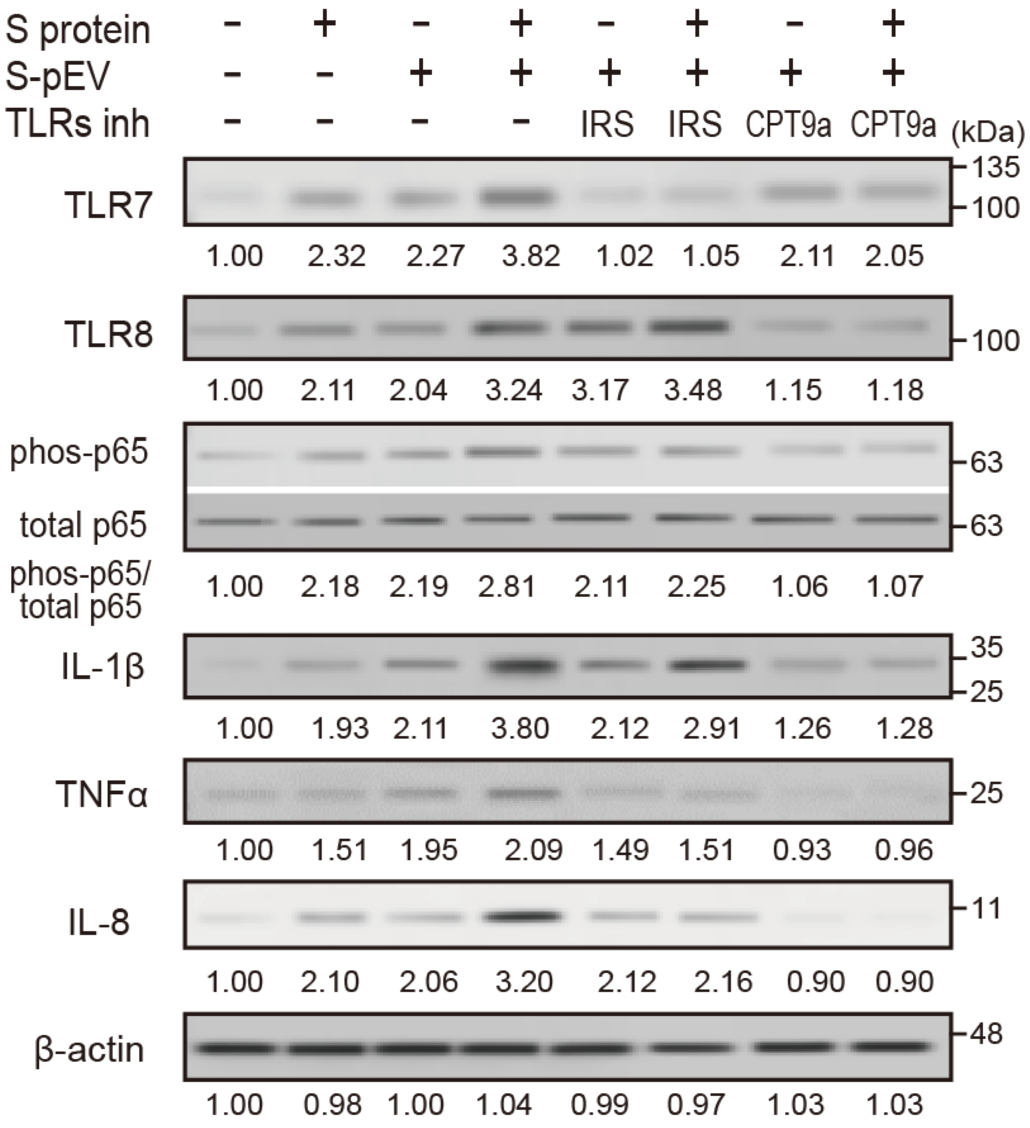

S protein - + - + - + - +
S-pEV - - + + + + + +
TLRs inh - - - - IRS IRS CPT9a CPT9a
(kDa)
TLR7
135
100
1.00 2.32 2.27 3.82 1.02 1.05 2.11 2.05
TLR8
100
1.00 2.11 2.04 3.24 3.17 3.48 1.15 1.18
phos-p65
63
total p65
63
phos-p65/ total p65
1.00 2.18 2.19 2.81 2.11 2.25 1.06 1.07
IL-1β
35
25
1.00 1.93 2.11 3.80 2.12 2.91 1.26 1.28
TNFα
25
1.00 1.51 1.95 2.09 1.49 1.51 0.93 0.96
IL-8
11
1.00 2.10 2.06 3.20 2.12 2.16 0.90 0.90
β-actin
48
1.00 0.98 1.00 1.04 0.99 0.97 1.03 1.03

TLR7 (121 kDa)

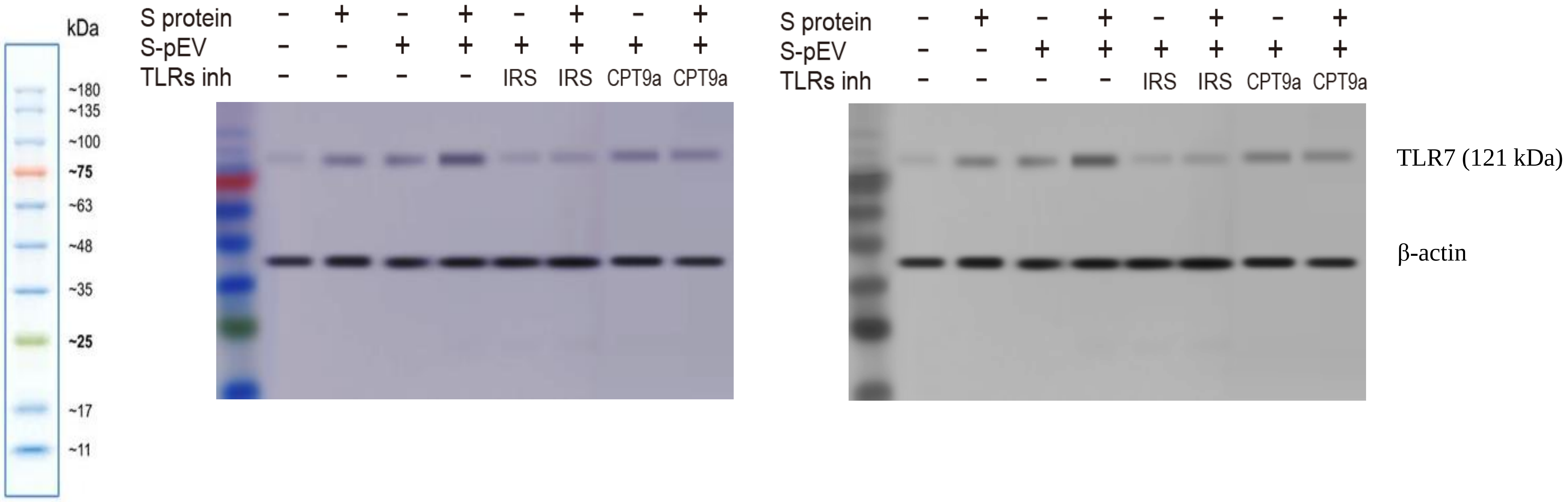
kDa
~180
~135
~100
~75
~63
~48
~35
~25
~17
~11
S protein - + - + - + - +
S-pEV - - + + + + + +
TLRs inh - - - - IRS IRS CPT9a CPT9a
S protein - + - + - + - +
S-pEV - - + + + + + +
TLRs inh - - - - IRS IRS CPT9a CPT9a
TLR7 (121 kDa)
β-actin

TLR8 (110 kD)

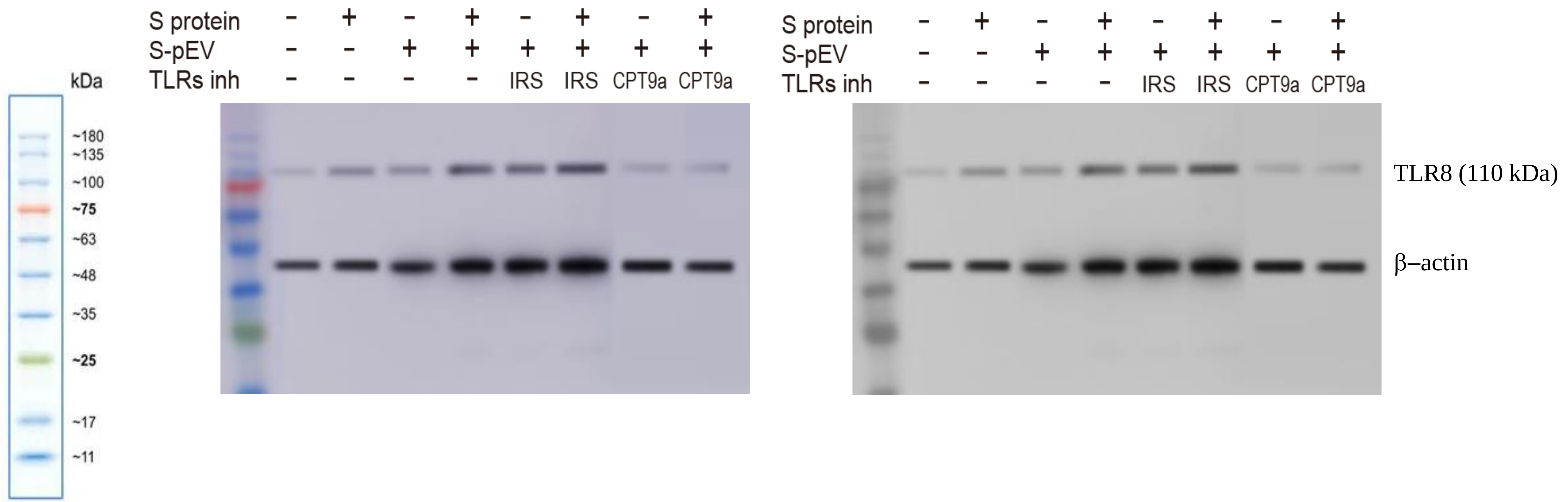

kDa
~180
~135
~100
~75
~63
~48
~35
~25
~17
~11
S protein - + - + - + - +
S-pEV - - + + + + + +
TLRs inh - - - - IRS IRS CPT9a CPT9a
S protein - + - + - + - +
S-pEV - - + + + + + +
TLRs inh - - - - IRS IRS CPT9a CPT9a
TLR8 (110 kDa)
β–actin

Phos-65 (65 kDa)

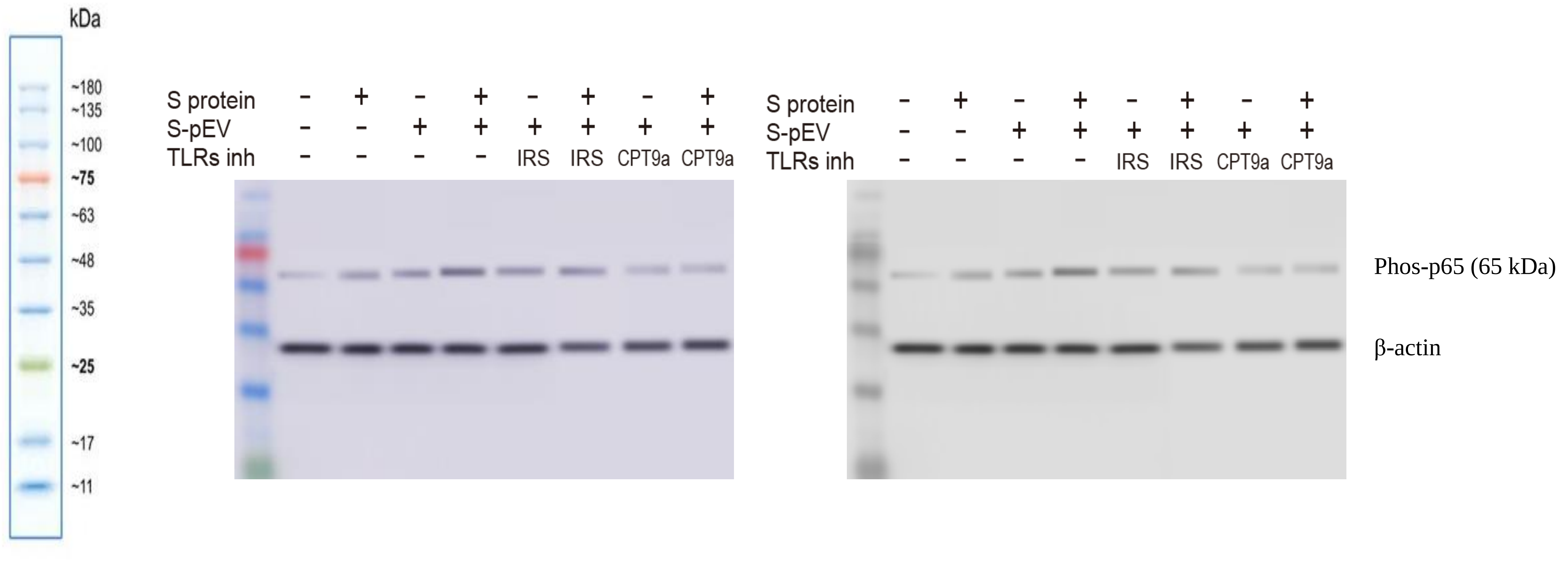

Total p65 (65 kDa)

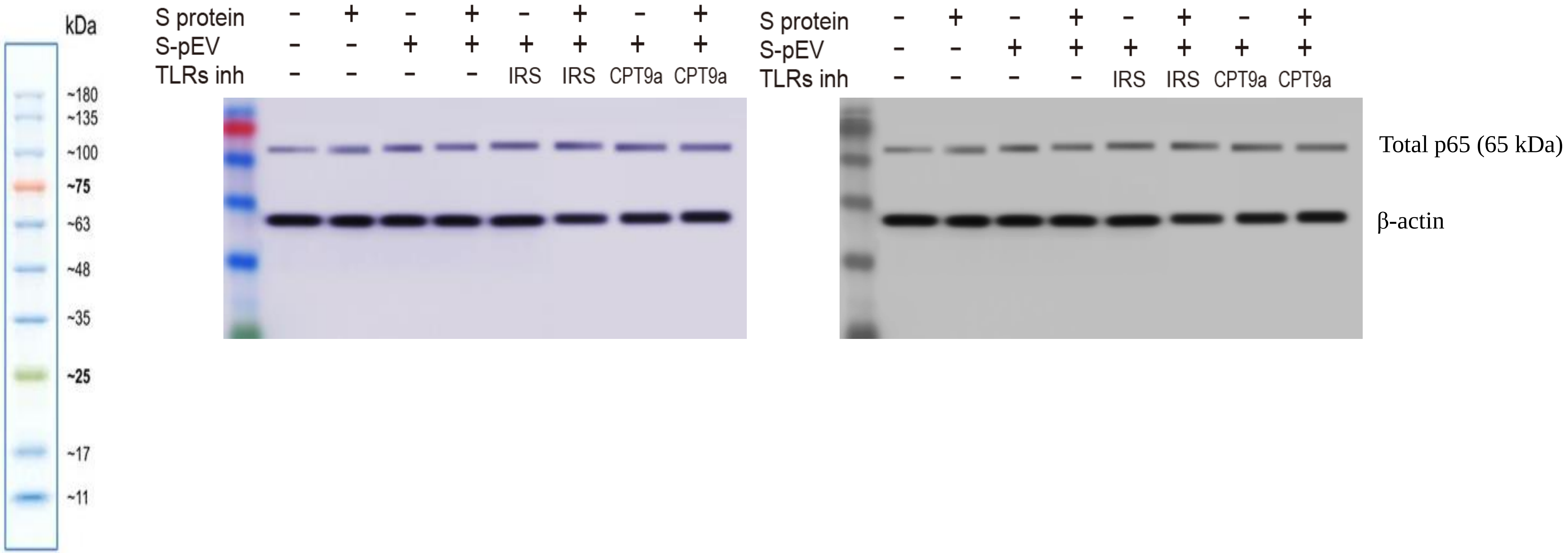

IL-1β (31 kDa)

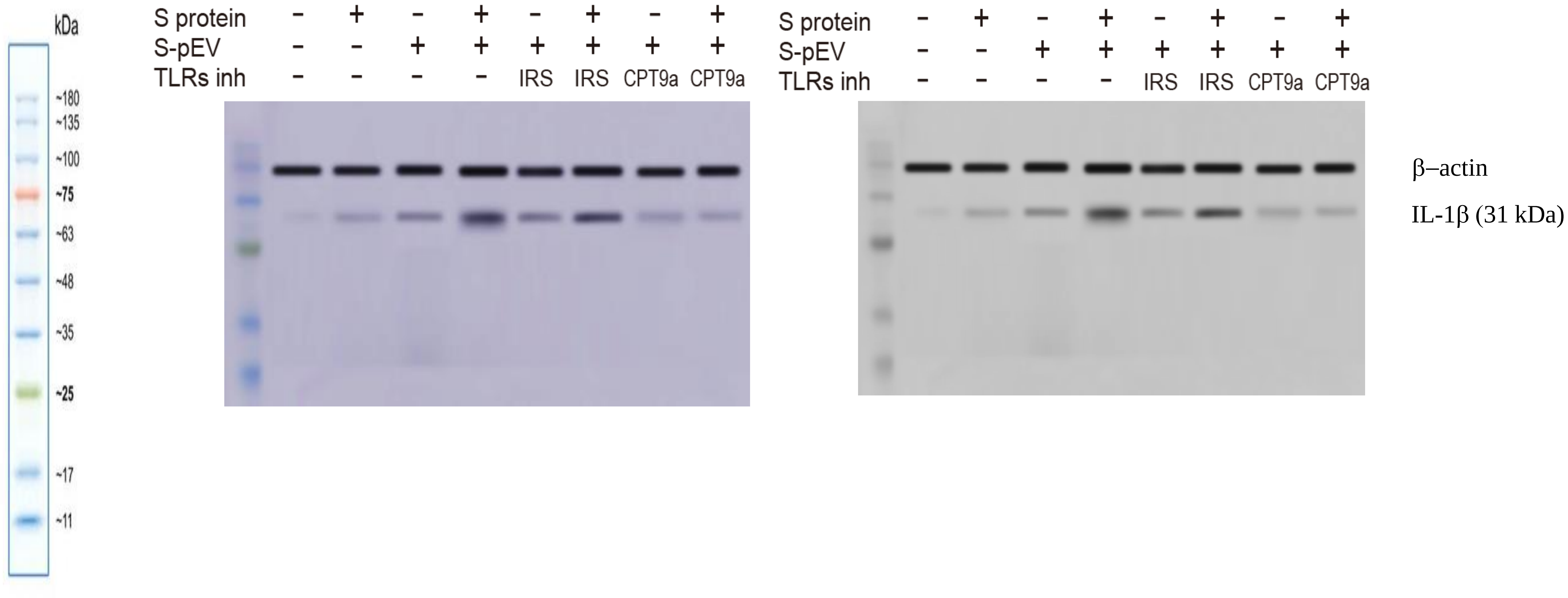
kDa
~180
~135
~100
~75
~63
~48
~35
~25
~17
~11
S protein - + - + - + - +
S-pEV - - + + + + + +
TLRs inh - - - - IRS IRS CPT9a CPT9a
S protein - + - + - + - +
S-pEV - - + + + + + +
TLRs inh - - - - IRS IRS CPT9a CPT9a
β–actin
IL-1β (31 kDa)

TNF-α (26 kDa)

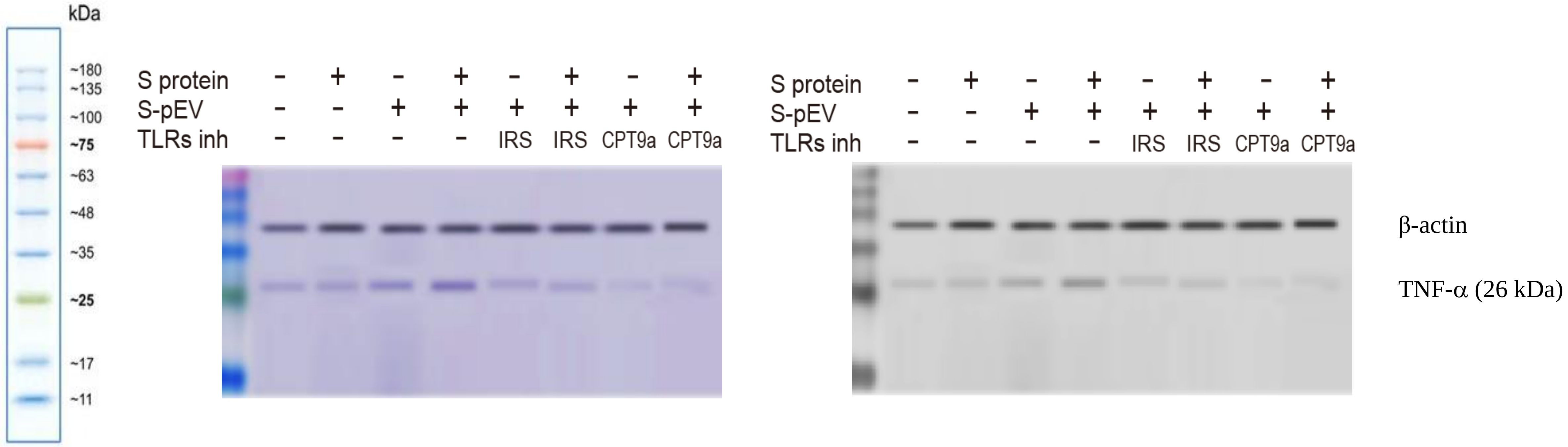
kDa
~180
~135
~100
~75
~63
~48
~35
~25
~17
~11
S protein - + - + - + - +
S-pEV - - + + + + + +
TLRs inh - - - - IRS IRS CPT9a CPT9a
S protein - + - + - + - +
S-pEV - - + + + + + +
TLRs inh - - - - IRS IRS CPT9a CPT9a
β-actin
TNF-α (26 kDa)

IL-8 (8 kDa)

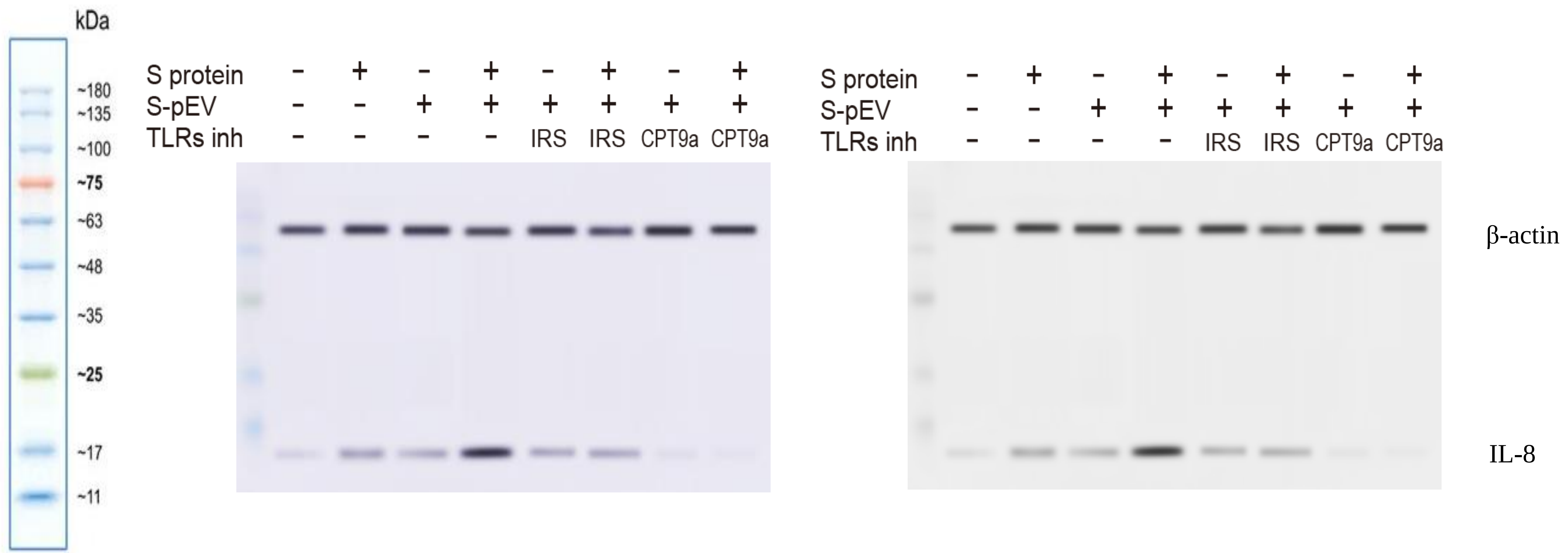
kDa
~180
~135
~100
~75
~63
~48
~35
~25
~17
~11
S protein - + - + - + - +
S-pEV - - + + + + + +
TLRs inh - - - - IRS IRS CPT9a CPT9a
S protein - + - + - + - +
S-pEV - - + + + + + +
TLRs inh - - - - IRS IRS CPT9a CPT9a
β-actin
IL-8
